# COVID-19 Vaccine Effectiveness Against Mortality in the Omicron Period: Evidence from Linked Mortality and Vaccination Records

**DOI:** 10.3390/vaccines13121235

**Published:** 2025-12-11

**Authors:** Sadia Farzana, Francesco Maria Rossi, Katie S. Allen, Qian Luo, Jeff Whittle, Kevin McGurk, Benjamin W. Weston, Andy Ye Yuan, Ali Moghtaderi, Vladimir Atanasov, Bernard Black

**Affiliations:** 1 Pritzker School of Law, Northwestern University, Chicago, IL 60611, USA; sadia.farzana@law.northwestern.edu (S.F.); francesco.rossi@law.northwestern.edu (F.M.R.); 2 Center for Biomedical Informatics, Regenstrief Institute, Indianapolis, IN 46202, USA; allenkat@regenstrief.org; 3 Department of Health Policy and Management, Milken Institute School of Public Health, George Washington University, Washington, DC 20053, USA; qluo@gwu.edu (Q.L.); moghtaderi@email.gwu.edu (A.M.); 4 Department of Medicine, Medical College of Wisconsin, Milwaukee, WI 53226, USA; jeffrey.whittle@va.gov; 5 Department of Emergency Medicine, Medical College of Wisconsin, Milwaukee, WI 53226, USA; kjmcgurk@mcw.edu (K.M.); beweston@mcw.edu (B.W.W.); 6 Levin College of Law, University of Florida, Gainesville, FL 32611, USA; yuan@law.ufl.edu; 7 Raymond A. Mason School of Business, William & Mary, Williamsburg, VA 23185, USA; vladimir.atanasov@mason.wm.edu

**Keywords:** COVID-19, COVID-19 mortality, cause of death, COVID Excess Mortality Percentage, vaccine effectiveness, healthy vaccinee bias, waning vaccine effectiveness, hybrid immunity

## Abstract

Objective: This study aimed to assess COVID-19 vaccine effectiveness against death (VE), controlling for healthy vaccinee bias. Methods: We link all adult deaths through year-end 2022 in the State of Indiana, U.S.A., to vaccination records and identify which deceased received primary vaccination (measured as either one or two initial doses) and which received one or two booster doses. We measure COVID-19 mortality with the COVID Excess Mortality Percentage (CEMP). CEMP is calculated, for a group defined by various characteristics (age, sex, time period), as COVID-19 deaths divided by non-COVID natural deaths. The CEMP outcome measure accounts for healthy vaccinee bias by using non-COVID natural mortality to control for differences in population health. Results: We find a large healthy vaccinee bias. Controlling for this bias, we find substantial VE for primary vaccination and the first booster dose during the first five vaccine-available calendar quarters, from 1Q2021 through 1Q2022 (end of Omicron infection wave). However, over 2Q–4Q2022, we find no evidence for primary-vaccination VE, and find moderate but statistically insignificant booster VE, which largely wanes by 4Q2022. Conclusions: It is known that by 2Q2022, most people had natural immunity from prior COVID-19 infection. Thus, our results for 2Q–4Q2022 largely reflect comparing hybrid (infection plus vaccination) immunity to infection-only immunity. In this period, we find negligible mortality benefit from primary vaccination, and moderate but waning benefit from a booster dose. Policy Implications: Controlling for healthy vaccinee bias is crucial when estimating VE. We found limited VE against COVID-19 mortality over 2Q–4Q2022, but lacked data for more recent periods.

## 1. Introduction

COVID-19 vaccination has markedly reduced COVID-19 mortality and prevented millions of deaths worldwide [1,2,3]. However, after the Omicron variant infection wave in the first quarter (1Q) of 2022, many people, especially unvaccinated persons, have been infected, often multiple times. Prior infection can produce a durable immune response against severe outcomes (hospitalization or death) [4,5]. This raises the question—how effective are primary vaccination (which we measure as either one or two initial doses) and booster doses against mortality in the post-Omicron-wave environment? We address this question using linked vaccination and mortality data from Indiana during the calendar years 2021 and 2022.

Numerous studies have examined COVID-19 vaccine effectiveness (*VE*) [6,7]. However, most vaccinated persons chose to be vaccinated, so these estimates are subject to selection bias. A key concern that is either not addressed at all or not well-addressed in most studies is healthy vaccinee bias (*HVB*)—the tendency for healthier individuals to be more likely to be vaccinated [8,9,10,11]. If *HVB* is not addressed, *VE* against serious disease and death will be overestimated because better outcomes among vaccinees will partly reflect their better underlying health [9,12].

Among prior studies of *VE* against mortality, many include only limited adjustment for individual characteristics, often only age and sex [13,14,15,16], or cover relatively short time periods [13,17,18,19]. Most U.S.-based analyses do not have access to population-level data [15,20,21,22,23], and studies with population data often have limited information on underlying health [16,24]. Many studies rely on a test-negative design [17,21,22]. However, the test-negative design relies on strong assumptions and can be vulnerable to selection bias when these assumptions are not satisfied [25,26].

In this study, we focus on *VE* against mortality. We use a novel method for evaluating *VE* against mortality that controls for *HVB*. This approach was used and validated in our prior work [27,28,29]. We use non-COVID natural mortality rates for groups defined by age and vaccination status to proxy for the underlying health of those groups. We use as an outcome measure, the COVID-19 Excess Mortality Percentage (*CEMP*), defined as COVID-19 deaths divided by non-COVID natural deaths, converted to a percentage. The idea behind this measure is that the *CEMP* denominator controls for health differences between two groups, such as vaccinated versus unvaccinated. We study primary vaccination (one or two doses) and the first and second booster doses.

We provide evidence on HVB, measure its magnitude, and validate the *CEMP* measure as a means of controlling for this bias. We then use *CEMP* as an outcome and measure the relative mortality risk of vaccinated versus unvaccinated persons (*RMR*) for Indiana over January 2021 through December 2022.

## 2. Data, Methods, and Sources of Potential Bias

### 2.1. Data and Basic Assumptions

We used vaccination data from the Indiana Health Information Exchange (IHIE) for all Indiana residents aged 15 years and older (N = 5,460,000), of whom 3,826,000 (70.1%) received at least one COVID-19 vaccine dose. The vaccination data were linked to death certificate data, which contain information on age at death, the decedent’s five-digit home ZIP code, manner of death, both ICD-10 codes and text fields for underlying cause of death and contributing conditions, sex, race/ethnicity, education, income, and marital and veteran status. Further sample details are in Supplementary Figure S1. 

We categorized race/ethnicity into four groups: non-Hispanic White (“White”), Black, non-Black Hispanic (“Hispanic”), and Other (including Asian, Native American, and mixed-race people). Area socioeconomic status (“SES”) was measured using the Graham Social Deprivation Index [30,31]. We used U.S. Census population estimates for Indiana as of 1 July 2020 to obtain counts of vaccinated persons in groups defined by age, sex, race/ethnicity, and SES. For each group, we estimated the number of unvaccinated persons as the Census population estimate minus the number of vaccinated persons. COVID-19 deaths were identified using the text fields for cause of death; counts were very similar when using ICD-10 codes derived from these text fields (Supplementary Table S1).

In estimating *VE*, we moved vaccination dates forward by 30 days. So, if a person received a first vaccine dose on day t, we treated this person as vaccinated beginning on day t + 30, and the same applied to additional doses. This 30-day lag was used to allow time (roughly 1–2 weeks) for a vaccine dose to become fully effective, plus a typical 2–3 week lag between infection and death; the 30-day lag also controls for the tendency for very sick people and people near the end of life to not become vaccinated (Supplementary Figure S13). We counted persons vaccinated with a given number of doses as of the 1st of each month, allowing for this lag. Our results are not sensitive to the lag period. See Supplementary Part VIII. We excluded immunocompromised decedents.

### 2.2. Variables and Regression Methods

We define *CEMP* for persons with x doses (*CEMP*_x_), the relative mortality risk of vaccinees versus unvaccinated persons (“*RMR*”), and vaccine effectiveness (*VE*) as

$$CEMP_{x}=100\;*\frac{COVID\;deaths_{x}}{non-COVID\;natural\;deaths_{x}}$$

$$RMR_{x,unvax}^{CEMP}=\frac{CEMP_{x}\;}{CEMP_{unvax}}\;VE_{x,unvax}^{CEMP}=1-RMR_{x,unvax}^{CEMP}$$

*RMR* of 0 implies perfect vaccine protection; *RMR* of 1.0 implies no protection. *RMR* greater than one would imply that vaccination causes harm (i.e., higher mortality among the vaccinated than among the unvaccinated).” $RMR_{x,unvax}^{CEMP}$ can be obtained by comparing *CEMP* ratios for two groups, or equivalently, as an odds ratio from logistic regression for a sample of decedents containing both groups. Note that these measures rely on absolute counts of deaths from COVID-19 versus other natural causes, within population groups defined by vaccination status. They do not depend on the accuracy of population estimates and thus do not depend on the precision of estimated mortality *rates*.

It is also useful to define a measure of healthy vaccinee bias. Let *NCNMR*_x_ denote the non-COVID natural mortality rate for the group with x vaccine doses:

$$NCNMR_{x}=\frac{non-COVID\;natural\;deaths_{x}}{Pop_{x}}$$

Then, define a healthy vaccinee bias correction factor (“*HVBCF*”) such that if healthier people are more likely to be vaccinated, 0 ≤ *HVBCF* < 1. Conversely, if less healthy people are more likely to be vaccinated, then *HVBCF* > 1. A value for the correction factor of 1 indicates no bias in either direction. Lower values of *HVBCF*, approaching zero, indicate progressively greater degrees of bias. If vaccinated people are healthier than unvaccinated people, they will have lower *NCNMR* rates:

$$HVBCF_{x,unvax}=\frac{NCNMR_{x}}{NCNMR_{unvax}}$$

This measure is valid under the assumption that COVID-19 vaccination does not substantially change non-COVID natural mortality.

One can also define an *RMR*^raw^ measure, based on raw COVID-19 mortality rates (*COVIDMR*), which is not adjusted for healthy vaccinee bias.

$$RMR_{x,unvax}^{raw}=\frac{COVIDMR_{x}}{COVIDMR_{unvax}}$$

*RMR*^CEMP^, *RMR*^raw^, and *HVBCF* are related; see Supplementary Material for derivation:

$$RMR_{x,unvax}^{CEMP}=\frac{RMR_{x,unvax}^{raw}}{HVBCF_{x,unvax}}$$

### 2.3. Confidence Intervals for RMR and Multivariate Logistic Regression

In a sample of persons who died of natural causes, *CEMP* equals the odds that the cause of death is COVID-19 versus another natural cause. $RMR_{x,unvax}^{CEMP}$ compares vaccinated to unvaccinated persons by taking the ratio of their *CEMP*s. It is thus an odds ratio. *RMR* can be obtained algebraically as above and can also be obtained by logistic regression applied to a sample of decedents. We obtained 95% confidence intervals (CIs) for *RMR*^CEMP^ using logistic regression, on groups of decedents defined by age and vaccination status. See Supplementary Material for computation of CIs for HVBCF.

We also conducted a multivariate logistic estimation of $RMR_{x,unvax}^{CEMP}$, in which we adjusted for other predictors of mortality risk, available from death certificates: age, age^2^, sex, race/ethnicity, education level, marital status, and area-SES. See Supplementary Part IX for details.

### 2.4. Validating the CEMP Approach

*CEMP* uses *NCNMR* as a proxy for the underlying health of a group and, hence, for its baseline mortality risk if unvaccinated. We assessed the validity of this approach by measuring the correlation of natural mortality in April–December 2019 (pre-COVID) and COVID-19 mortality during the same months in 2020—during the COVID-19 period, but before COVID-19 vaccines were available in the United States. We compared COVID-19 mortality in the 2020 period to natural mortality in the pre-COVID 2019 period for population groups defined by age, sex, race/ethnicity, and socioeconomic status.

### 2.5. Counting Vaccine Doses

IHIE matches vaccinees across different numbers of doses and matches vaccinees to death records. We do not have access to their matching algorithm. We see some evidence in the data that, for about 1–2% of reported vaccinees, IHIE treats two vaccinated people as a single “person.” In Supplementary Part III, we develop rules to estimate the number of doses received by an actual person.

### 2.6. Possible Sources of Bias

National studies indicate that deaths directly or indirectly due to COVID-19 are somewhat under-recorded in death records [32,33]. In Indiana, we also see indications of undercounting (Supplementary Figure S5). Undercounting of COVID-19 deaths will lead *CEMP* values to be understated. However, *RMR^CEMP^* estimates will be biased only if the degree of undercounting differs between vaccinated and unvaccinated people. We cannot test this possibility with our data, but we have no reason to expect differential undercounting.

Second, bias will arise if IHIE undercounts vaccinee deaths by not fully matching them to mortality records. We observed apparent undercounting over August–October 2021 (Supplementary Part IV). If IHIE undercounts vaccinee deaths, it must overcount deaths of unvaccinated persons. If vaccination reduces COVID-19 mortality, then the overcounted deaths of unvaccinated persons will disproportionately involve non-COVID causes. This will cause us to underestimate *CEMP*_unvax_ and therefore overestimate $RMR_{vax,unvax}^{CEMP}$ = $\frac{CEMP_{vax}\;}{CEMP_{unvax}}$.

We address this issue as follows. To estimate monthly death counts for August through October 2021, we interpolated death counts for vaccinated decedents between July and November 2021. We assume, as seems plausible, that the percentage undercount was similar for COVID-19 and other natural deaths; if so, the undercounting will not affect *CEMP*_vax._ We also know *CEMP* for the full population (vaccinated and unvaccinated), from mortality records; overall, *CEMP* is a mortality-weighted average of *CEMP*_vax_ and *CEMP*_unvax_. This is sufficient to let us algebraically compute *CEMP*_unvax_ and thus $RMR_{vax,unvax}^{CEMP}$. See Supplementary Material for details. The adjustments are small but visible in graphs for 3Q2021, and nearly invisible for 4Q2021. Our reported results use adjusted *RMR*^CEMP^ for these two quarters; Supplementary Figure S7 shows both raw and adjusted values.

## 3. Results

### 3.1. Validating the CEMP Measure

**Figure 1:**
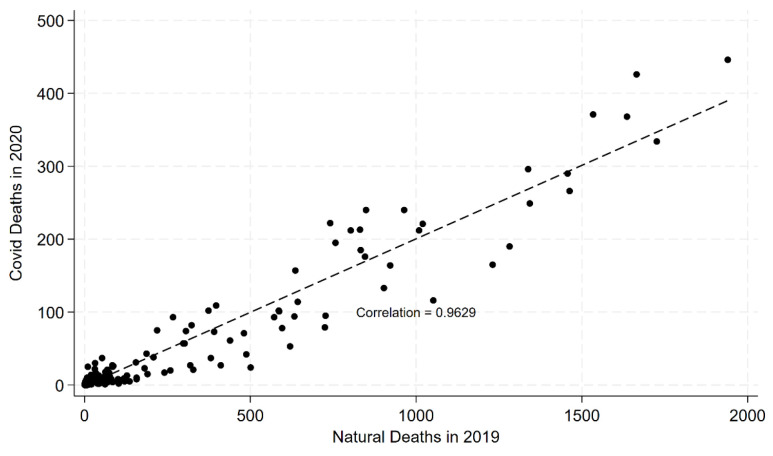
**Correlation between Natural Mortality in 2019 and COVID-19 Mortality in 2020.** Figure shows scatterplot of natural mortality in Indiana over April–December 2019 (x-axis) against COVID-19 mortality over April–December 2020 (y-axis), for groups defined by age (15–39, 40–49, 50–59, 60–69, 70–79, 80–89, 90+), sex, race/ethnicity, and area SES quintile. The figure also shows a best-fit regression line and a Pearson correlation coefficient.

The *CEMP* measure uses group *NCNMR* as a proxy for underlying group health and, therefore, for group risk of COVID-19 death. This proxy requires empirical validation. Figure 1 plots, for Indiana, the number of natural deaths over April–December 2019 (pre-COVID) (x-axis) against COVID-19 mortality counts over the same months in April–December 2020 (COVID period, pre-vaccine) (y-axis), for groups defined by age (15–39, 40–49, 50–59, 60–69, 70–79, 80–89, 90+), sex, race/ethnicity, and area-SES quintile. The Pearson correlation coefficient is 0.963, indicating that non-COVID-19 natural mortality is a strong predictor of COVID-19 mortality among unvaccinated persons. Supplementary Figure S2 shows a similar correlation between natural mortality rates (instead of counts) over April–December 2019 and COVID-19 mortality rates over April–December 2020. In Supplementary Figure S3, we confirm that all-cause natural mortality in Indiana in 2019 is highly correlated with *NCNMR* in 2020 (r = 0.999).

Additional validation is provided by the multivariate logistic regression analysis discussed below. Within groups defined by age and time period, *RMR*^CEMP^ estimates are very similar to the corresponding multivariate estimates. This similarity suggests that the simple *CEMP* measure adjusts effectively for underlying differences in population health.

### 3.2. Summary Statistics

Table 1 provides summary statistics by period over 2021–2022. Starting in 2Q 2022, COVID-19 deaths, and hence *CEMP*, declined sharply, whereas $RMR_{vax,unvax}^{CEMP}$ rose.

Supplementary Figure S4 plots vaccination rates over time. Overall, by year-end 2022, around 70% of Indiana residents aged 15+ received at least one dose (Supplementary Table S3), with higher rates for older persons. By year-end 2022, over 90% of people age 60+ were vaccinated, with most receiving a booster (Table 1).

### 3.3. Evidence for Healthy Vaccinee Bias

**Figure 2:**
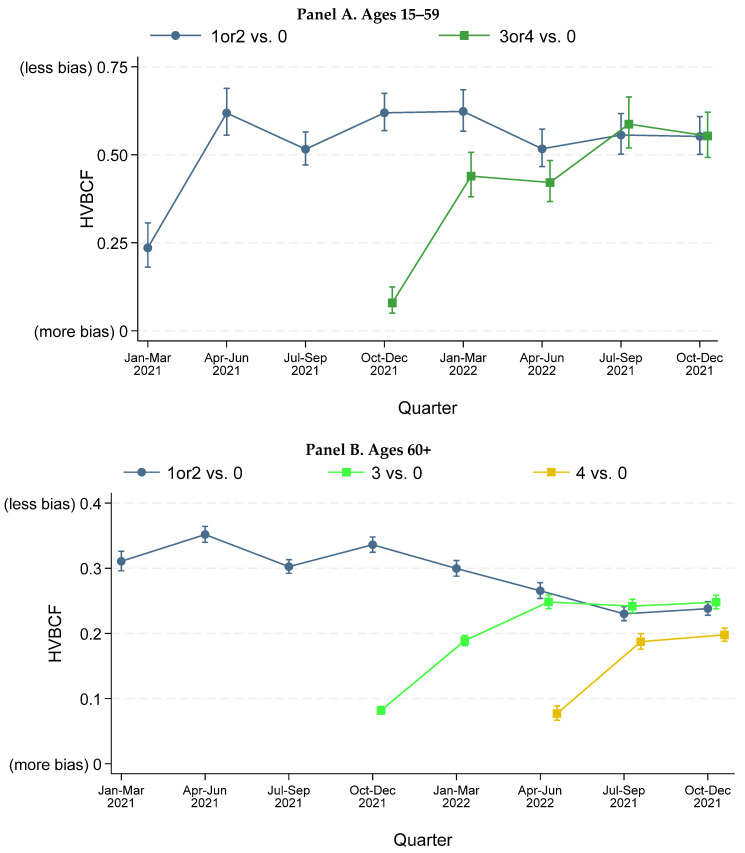
**Healthy vaccinee bias correction factor by age range and number of doses.** The figure shows the healthy vaccinee bias correction factor (*HVBCF*) by calendar quarter over 1Q2021–4Q2022, for vaccinees aged 15+ with the indicated number of vaccine doses. “1 or 2” means primary vaccination; “3” doses means 1 booster dose; “4” doses means 2 booster doses (**A**). Ages 15–59. The results for 3 and 4 doses (1 or 2 booster doses) are combined because very few people in this age range received a fourth dose during the study period (**A**,**B**). *HVBCF* is the ratio of *non-COVID NMR* with indicated numbers of vaccine doses for vaccinated to *non-COVID NMR* for unvaccinated. Estimates for 3Q–4Q2021 are adjusted for the IHIE undercount of vaccinated decedents during August–October 2021. See Supplementary Table S4 for underlying data. The sample excludes immunocompromised persons. Short vertical lines show 95% confidence intervals. Higher *HVBCF* indicates less bias. Note that Panels A and B use different y-axis scales.

Figure 2 provides $HVBCF_{x,unvax}$ values for ages 15–59 and 60+. See Supplementary Figure S8 for finer age groups. Healthy vaccinee bias was substantial at all ages, with tight CIs for *HVBCF*. For ages 15–59, the *HVB* correction factor for primary vaccination versus unvaccinated ($HVBCF_{x,unvax}$) was around 0.6 through 1Q2022, and declined slightly after that. These levels indicate that vaccinees had much better background health (lower risk of non-COVID natural mortality) than the unvaccinated. Healthy vaccinee bias is higher (HCBCF is lower) in the quarter when vaccination first becomes available, for both primary vaccination and booster doses.

Healthy vaccinee bias was more pronounced (*HVBCF* was lower) among adults aged 60+, who account for most COVID-19 deaths. $HVBCF_{x,unvax}$ for primary vaccination was around 0.3 for 1Q2021–1Q2022 and declined slightly thereafter. This decline is driven by ages 80+ (Supplementary Table S4). For ages 60+, the additional tendency for healthier people to get vaccinated in the first quarter when a vaccine dose becomes available is seen for booster doses, but not for primary vaccination. The magnitude of healthy vaccinee bias for persons receiving only primary vaccination versus those also receiving booster doses tends to converge over time.

Given healthy vaccinee bias at these levels, *RMR*^raw^ will be well below *RMR*^CEMP^. *RMR*^raw^ levels will suggest that vaccination is more effective against mortality than it actually is. This is especially true for booster doses, soon after they become available. Indeed, relying on *RMR*^raw^ can imply that vaccination is effective against mortality, even if it is not. We observe this pattern of apparent but not actual effectiveness for primary vaccination for 2Q2022–4Q2022 (see Supplementary Figure S12).

### 3.4. RMR^CEMP^ for Primary Vaccination

**Figure 3:**
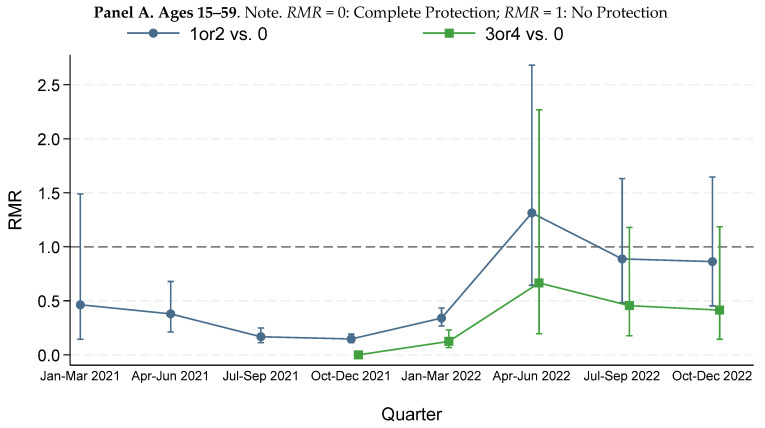

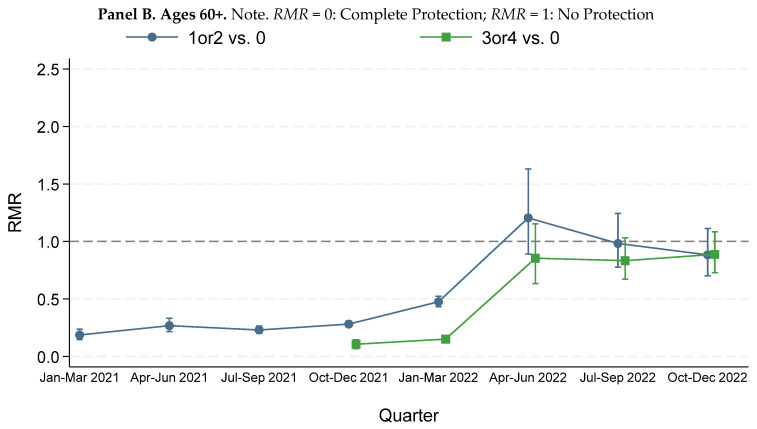
**Relative mortality risk by age range and number of doses.** The figure shows relative mortality risk (*RMR*), based on *CEMP*, by calendar quarter for vaccinees aged 15+ with the indicated number of vaccine doses, for 1Q2021–4Q2022. “1 or 2” means primary vaccination. “3 or 4” means 1 or 2 booster doses. (**A**). Ages 15–59. The results for 3 and 4 doses (1 or 2 booster doses) are combined because very few people in this age range received a fourth dose during the study period. (**B**). Age 60+. Both panels. Estimates for 3Q–4Q2021 are adjusted for the IHIE undercount of vaccinated decedents during August–October 2021. Vertical lines show 95% confidence intervals.

Figure 3 reports quarterly $RMR_{vax,unvax}^{CEMP}$ for persons with different numbers of vaccine doses for ages 15–59 and 60+. See Supplementary Figure S9 for results for finer age groups.

The time patterns in Figure 3 reflect several important factors. One is the evolution of virus variants. The Alpha variant was dominant in 2Q2021, Delta in 3Q–4Q2021, and Omicron beginning in 1Q2022. Higher *RMR* in 1Q2022 could reflect the shift to Omicron dominance.

A second is the number of already infected people, which grew rapidly in 1Q2022 with the huge Omicron infection wave. By the end of 1Q2022, roughly half of vaccinated persons had been infected, and thus benefited from hybrid immunity, as had a large percentage of unvaccinated persons [34]. Thus, $RMR_{vax,unvax}^{CEMP}$ increasingly measured a blended average of the benefit of hybrid immunity (for many vaccinees) or vaccination alone (for some vaccinees) versus infection-based immunity (for most unvaccinated persons).

The third was waning *VE* for the mRNA vaccines, beginning roughly 5–6 months after primary vaccination. Evidence of waning led to booster dose approval in late September 2021, initially for older and other high-risk persons.

Against that background, consider first *RMR*^CEMP^ for primary vaccination. Primary vaccination has a substantial protective effect through 1Q2022. Protection weakens in 1Q2022 and essentially disappears after that for both age groups. Point estimates are close to 1.0 (no protective effect), and indeed above 1.0 in 2Q2022, although not significantly so. CIs are wide, especially for younger persons, reflecting low COVID-19 mortality rates. However, the lower end of the CIs for 2Q2022 is well above estimates for earlier periods.

### 3.5. Booster Dose RMR^CEMP^

There is strong evidence for both age groups that a booster dose offered additional protection through 1Q2022. After that, *RMR*^CEMP^ point estimates for the booster dose are generally below those for primary vaccination, but CIs overlap, so booster benefit must be viewed as suggestive but not definitive. By 4Q2022, *RMR*^CEMP^ estimates for primary vaccination versus booster converged for ages 60+.

In Supplementary Figure S9, we provide separate estimates for ages 60–79 and 80+, and for first and second booster doses. There is limited evidence for additional protection from a second booster dose.

### 3.6. Multivariate Estimates

Supplementary Table S7 reports estimates of ${RMR}_{\mathrm{vax},\;\mathrm{unvax}}^{CEMP}$ from a multivariate logistic model for vaccinees receiving different numbers of doses. Adding additional predictors has no practical effect on *RMR*^CEMP^ estimates. All multivariate point estimates are within the univariate CIs and vice versa.

### 3.7. Additional Analyses

In Supplementary Table S8 and Figure S10, we report *RMR*^CEMP^ separately for men and women. Levels are similar for both groups, and CIs overlap.

We report *RMR*^raw^ values in Supplementary Table S6 and compare *RMR*^CEMP^ and *RMR*^raw^ in Supplementary Figure S11. *RMR*^raw^ is substantially lower than *RMR*^CEMP^, consistent with substantial *HVB*.

In Supplementary Figure S12, we compare our Indiana results to those for Milwaukee County, Wisconsin [27]. Estimates are similar, with overlapping CIs.

In Supplementary Figure S13, we report all-cause natural mortality based on days since vaccination (without the 30-day lag). These graphs confirm the intuition that very ill people are less likely to become vaccinated. Thus, *HVBCF* would be lower (larger bias) without the 30-day lag. In unreported results, we found slightly higher *RMR*^CEMP^ values using a 14-day lag.

## 4. Discussion

### 4.1. HVB and Need for a Credible Counterfactual

*VE* and *RMR* estimates depend on having a credible counterfactual: what COVID-19 mortality among vaccinees would have been without vaccination. Our results show large differences in background health, proxied by non-COVID-19 natural mortality: vaccinated people are substantially healthier than unvaccinated people. We provide evidence that healthy vaccinee bias is large, especially for older people. Healthy vaccinee bias is especially strong soon after vaccine doses become available for initial vaccination for ages 15–59, and for booster doses for all ages. This is evidence that healthier individuals tend to be the first to be vaccinated. Unless this selection is controlled for (using our *CEMP* measure or other methods), estimates of *VE* and *RMR* will be heavily biased.

*CEMP* has several advantages as a measure of COVID-19 mortality when studying vaccine effectiveness. It relies solely on death certificates, which are available for all decedents; it does not require population estimates; and it can address healthy vaccinee bias by using *NCNMR* as a proxy for underlying population health. An alternative approach, controlling for health history, including hospitalizations and comorbidities, would require population-level health history data, which are not available in U.S. datasets. Moreover, a study in Qatar, where complete health records are available, suggests that adjusting for health history is insufficient to address healthy vaccinee bias [11], as does an Israeli study of non-COVID-19 mortality [12].

The large magnitudes of healthy vaccinee bias that we found are consistent with evidence of similar levels of healthy vaccinee bias in Czechia [9]. They are also consistent with U.S. evidence showing much lower non-COVID mortality among vaccine recipients across seven integrated health systems over December 2020–July 2021 [35], which persisted after controlling for individual health [36]. *HVB* can also explain differences in healthcare spending for vaccinated versus unvaccinated persons [37].

Higher *HVB* when a vaccine dose is first available places stress on both controlling for *HVB* and measuring *RMR* over a period long enough so that *HVB* has stabilized. This problem was especially acute for COVID-19 because of intense public interest in obtaining evidence on *VE*, both for initial vaccination and booster doses. This led to a race to publish based on data for short time periods. Use of short time periods inadvertently magnified the effect of healthy vaccinee bias. Note too that a decline in healthy vaccinee bias over time and waning *VE* will both tend to produce rising apparent *RMR*. Thus, one cannot assess the extent of *VE* waning without also addressing *HVB*.

*NCNMR* predicts COVID-19 mortality well in the pre-vaccine period (Figure 1). This suggests that *CEMP* effectively controls for *HVB* and that *RMR*^CEMP^ provides a good estimate of *VE* relative to the counterfactual mortality of the same people had they remained unvaccinated. We note that a study in Czechia used a similar approach to measure healthy vaccinee bias and found similar magnitudes of bias [9].

### 4.2. Reduced VE in 2022

We find substantially higher $RMR_{vax,unvax}^{CEMP}$ in 2022. From 2Q2022 on, primary vaccination no longer provided meaningful protection for an average vaccinee. Point estimates for boosters were clinically significant but not statistically significant, and for ages 60+, they shrank over 2Q–4Q2022. Reduced protection likely reflects both vaccine waning and the changing nature of the comparison between vaccinated and unvaccinated. Immune imprinting from primary vaccination could also play a role [38,39,40,41].

*RMR*^CEMP^ values above 1.0 for primary vaccination in 2Q2022 could reflect already frail people dying of COVID-19 during the 1Q2022 Omicron wave, with a stronger pull-forward effect for the unvaccinated.

Our results reflect a blended average across vaccinees, including some who were already infected and others not, as well as individuals vaccinated some time ago and others more recently. Our results do not rule out some benefit for the never-infected, nor some benefit from new vaccination, for the period before waning takes place.

### 4.3. Comparison to Other COVID-19 Vaccine Effectiveness Studies

We summarize selected studies here.

#### 4.3.1. Vaccine Effectiveness in 2021 and 1Q2022

Even in 2021, we find *RMR* for vaccinees to be substantially higher (lower *VE*) than most other studies, both here and in prior work [27]. By comparison, two systematic reviews find two-dose *RMR*s during 2021 in the range of 6–17% [6,7]. Our higher *RMR*s reflect the use of *CEMP* as an outcome in order to address *HVB*. Two studies that control for health report *RMR*s closer to those reported here [21,34].

Other studies, although not controlling for healthy vaccinee bias, also report rising *RMR*s over time. A Norwegian study estimated two-dose *RMR* against death in the second half of 2021 at 6.6% after 10–17 weeks, rising to 31.4% after 33+ weeks [24]. A Hungarian study with follow-up through February 2022 found that two-dose *RMR* increased with age, reaching 43% among individuals aged 85 and older [16].

#### 4.3.2. Vaccine Effectiveness over 2Q–4Q2022

We discuss here studies that cover periods through at least 2Q2022 and include at least 4 months after vaccination, to allow for waning. Johnson et al. (2023) report a large drop in an age-standardized relative risk ratio (roughly, 1/RMR) for vaccinated versus unvaccinated from 16.2 in the Delta period to 5.3–5.4 after the Omicron wave [42]. This fall in relative risk is directionally consistent with the rise in *RMR*^CEMP^ that we find after 1Q2022. Atkeson and Kissler (2024) also report a large rise in *RMR*^raw^ in 2Q2022 [43].

Lin et al. (2022) report booster versus primary vaccination in North Carolina through 3 June 2022 [4]. They report incremental *VE* around 50% and dropping, as booster effectiveness waned. Their and our results are consistent, given both studies’ CIs.

Cai et al. (2025) studied U.S. veterans who received a booster in late 2024 [23]. They address *HVB* by comparing combined flu-COVID vaccine recipients with flu-vaccine-only recipients among veterans who received COVID vaccination in 2023. They use Veterans Administration data to control for patient health and demographics. They report a 64% reduction in all-cause mortality within 30 days of COVID-19 infection. This outcome includes deaths from causes unrelated to COVID-19, and is thus vulnerable to residual healthy vaccinee bias, not captured by their control group. In any case, our booster point estimates are within their CIs.

## 5. Limitations

We had data only for one state, Indiana, only through 2022, and studied only mortality. Indiana would not be seen in the U.S. as an outlier among states. Moreover, there is evidence, discussed above, that healthy vaccinee bias exists more broadly in the U.S. and in other countries.

Low COVID-19 mortality rates in 2022 cause limited statistical power.

We studied *VE* only against mortality, not against infection or hospitalization. The *CEMP* approach to controlling for healthy vaccinee bias may not apply to less severe outcomes such as hospitalization.

We do not observe prior COVID-19 infection in our data.

By 2Q2022, many people already had COVID [44,45]. Thus, *VE* during this period largely reflects the added protection from hybrid immunity (prior infection plus vaccination) relative to natural immunity alone.

We did not observe individual health, except through the limited lens of death certificates. *CEMP* may be an imperfect proxy for background health and may not fully capture behavioral differences between vaccinated and unvaccinated [27].

Since *CEMP* is measured at the population level, applying our *RMR* estimates to individuals is possibly an ecological fallacy (i.e., what applies to the group is not necessarily applicable to individuals). However, controlling for non-COVID mortality risk at the individual level is infeasible; it would require a randomized trial on a huge population, whereas the *CEMP* method is suitable for population-based observational studies using mortality datasets linked to vaccination records.

Some underreporting of COVID-19 deaths is likely, but this will bias *RMR*^CEMP^ estimates only if the degree of underreporting differs between vaccinated and unvaccinated people.

We assumed that vaccination does not materially change non-COVID mortality [46,47,48,49,50] and that COVID-19 infection itself does not alter non-COVID mortality. Higher near-term mortality from other causes [51,52] will bias *CEMP* downward, but will not affect *RMR*^CEMP^ as long as this bias is similar for vaccinated and unvaccinated groups.

## 6. Conclusions

We used a novel outcome measure, *CEMP*, to study how vaccination affects COVID-19 mortality risk over 2021–2022. We found substantial healthy vaccinee bias. Using the *CEMP* measure to control for this bias, we found substantial vaccine protection through 1Q2022, but no significant protection from primary vaccination after that. We found suggestive evidence of additional protection from a booster dose, but by 4Q2022, *RMR*^CEMP^ point estimates for ages 60+ were close to 1.0 (no protection). These findings suggest that protection from COVID-19 booster doses against mortality may be limited, and perhaps short-lived, in the post-Omicron environment in which most people have been infected, sometimes several times. There is a need for similar studies that control for healthy vaccinee bias and cover more recent periods.

## Figures and Tables

**Table 1:** **Summary statistics.** The table provides selected summary statistics for Indiana vaccinees and unvaccinated persons, age 15+, as of the indicated dates or periods. Counts of vaccinated persons are as of the end of the indicated period; deaths and ratios are averages for the period. See Supplementary Table S4 for additional breakdown by age group, time period, and number of vaccine doses. For 4Q2021–4Q2022, the total population slightly exceeds the number unvaccinated or with 1–2 or 3+ doses due to a small number of persons with unusual vaccination patterns.

	1Q 2021	2Q 2021	3Q 2021	4Q 2021	1Q 2022	2Q 2022	3Q 2022	4Q 2022
**Ages 15–59**								
Unvaccinated	3,174,369	2,182,349	1,814,061	1,616,807	1,526,851	1,508,934	1,495,444	1,487,532
1 or 2 doses	740,030	1,732,050	2,077,863	1,671,388	1,534,216	1,523,817	1,518,507	1,503,048
3+ doses	0	0	12,328	613,971	839,397	868,300	886,482	908,820
Total population	3,914,399	3,914,399	3,914,399	3,914,399	3,914,399	3,914,399	3,914,399	3,914,399
COVID-19 deaths	236	133	474	741	540	35	53	47
Non-COVID natural deaths	2106	1936	2093	2227	2054	1860	1895	2165
*COVID-MR*	0.006%	0.003%	0.012%	0.019%	0.014%	0.001%	0.001%	0.001%
*Non-COVID NMR*	0.054%	0.049%	0.053%	0.057%	0.052%	0.048%	0.048%	0.055%
*CEMP*	11.21%	6.87%	22.65%	33.27%	26.29%	1.88%	2.80%	2.17%
$RMR_{vax,unvax}^{CEMP}$	0.463	0.379	0.168	0.143	0.286	1.102	0.722	0.693
*HVBCF*_vax,unvax_	0.236	0.619	0.515	0.542	0.566	0.484	0.566	0.551
**Ages 60+**								
Unvaccinated	468,891	301,865	234,663	172,633	159,946	154,055	149,899	146,076
1 or 2 doses	1,076,189	1,243,215	1,223,629	583,884	507,223	496,990	489,429	473,820
3+ doses	0	0	58,314	755,604	837,028	860,397	871,663	890,326
Total population	1,545,080	1,545,080	1,545,080	1,545,080	1,545,080	1,545,080	1,545,080	1,545,080
COVID-19 deaths	2424	488	1355	2624	2578	262	475	532
Non-COVID natural deaths	13,162	12,560	13,070	14,074	13,751	12,377	12,779	14,252
*COVID-MR*	0.157%	0.032%	0.088%	0.170%	0.167%	0.017%	0.031%	0.034%
*Non-COVID NMR*	0.852%	0.813%	0.846%	0.911%	0.890%	0.801%	0.827%	0.922%
*CEMP*	18.42%	3.89%	10.37%	18.64%	18.75%	2.12%	3.72%	3.73%
$RMR_{vax,unvax}^{CEMP}$	0.188	0.269	0.231	0.263	0.321	0.997	0.883	0.885
*HVBCF*_vax,unvax_	0.311	0.352	0.297	0.255	0.229	0.238	0.224	0.228

## Data Availability

The data for this study was provided by the Wisconsin Department of Health Services and the Regenstrief Institute at Indiana University, is subject to data use agreements, and cannot be publicly shared.

## Supplementary Materials

The following supporting information can be downloaded at: https://www.mdpi.com/article/10.3390/vaccines13121235/s1. Refs. [53,54,55,56,57,58,59,60,61] are cited in Supplementary Material.
