# Supplementary material for: COVID-19 Vaccine Effectiveness Against Mortality in the Omicron Period: Evidence from Linked Mortality and Vaccination Records
Source: Vaccines (Basel). 2025 Dec 11;13(12):1235. doi: 10.3390/vaccines13121235 (PMC12737531; doi:10.3390/vaccines13121235)

Supplemental Material for

COVID-19 Vaccine Effectiveness against Mortality in the Omicron Period: Evidence from Linked Mortality and Vaccination Records

Sadia Farzana
Northwestern University

Francesco Maria Rossi
Northwestern University

Katie S. Allen
Regenstrief Institute

Qian (Eric) Luo
George Washington University

Jeff Whittle
Medical College of Wisconsin

Kevin McGurk
Medical College of Wisconsin

Benjamin W. Weston
Medical College of Wisconsin

Andy Ye Yuan
University of Florida

Ali Moghtaderi
George Washington University

Vladimir Atanasov
William & Mary, Mason School of Business

Bernard Black*
Northwestern University, Pritzker School of Law and Kellogg School of Management

This Supplemental Material can be downloaded from SSRN at:
http://ssrn.com/abstract=5641833

The paper can be downloaded from SSRN at:
http://ssrn.com/abstract=5641750

Supplemental Material for

COVID-19 Vaccine Effectiveness against Mortality in the Omicron Period: Evidence from Linked Mortality and Vaccination Records

Abstract

This Supplemental Material provides dataset and methods details, as well as additional results and robustness checks, for Farzana et. al, *COVID-19 Vaccine Effectiveness against Mortality in the Omicron Period: Evidence from Linked Mortality and Vaccination Records,* Vaccines *(2025)*.

Contents

II. Indiana Population Estimates 2
III. Details on Counting Vaccinated Persons and Number of Vaccine Doses 2
IV. Adjusting for Undercounting of Vaccinated Decedents 5
  A. Adjustment for IHIE Undercount of Vaccinated Decedents 5
  B. Estimating Confidence Intervals for Different Numbers of Vaccine Doses 7
V. Identifying COVID-19 Decedents from Cause of Death Fields 8
VI. Evidence on Excess Natural Non-COVID Deaths 9
VII. Virus Variants and Periods of Dominance 10
VIII. Measuring Population by Number of Vaccine Doses 10
IX. Time Period Studied, Variable Definitions, and Other Methods Details 11
  A. *CEMP*, *VE*, and *RMR* 11
  B. Univariate Logistic Regression and Confidence Intervals 13
  C. Multivariate Logistic Regression 14
  D. Confidence Intervals for HVBCF 14
X. Validating the *CEMP* Measure 15
XI. Additional Results for *RMR*, *HVBCF*, and *VE* 15
XII. Effects of Vaccination-Avoidance by the Very Ill 15
XIII. Defining the Immune-Compromised 16
XIV. Raw *RMR* (Using COVID-19 Mortality Rates Rather than *CEMP*) 16
XV. *RMR* in Indiana versus Milwaukee County, Wisconsin 16
XVI. Mortality-Weighted versus Population-Weighted Results 16
Additional References for supplementary materials (not cited in the text) 1
Table S-1. Comparison of COVID-19 deaths per text analysis, to ICD-10 codes 2
Table S-2. Vaccine Approval Dates 2
Table S-3. Summary Statistics on Vaccination Status 3
Table S-4. Healthy Vaccinee Bias Correction Factor (HVBCF)". 4
  Panel A. Healthy Vaccinee Bias Correction Factor for Ages 15-59 and 60+ 4
  Panel B. Healthy Vaccinee Bias for Ages 15-39 and 40-59 5
  Panel C. Healthy Vaccinee Bias for Ages 60-79 and 80+ 7
Table S-5. Relative Mortality Risk (Based on *CEMP*) 9
  Panel A. Relative Mortality Risk (*RMR*) for Broad Age Groups: Ages 15-59 and 60+ 9
  Panel B. Relative Mortality Risk (*RMR*) for Ages 15-39 and 40-59 10
  Panel C. Relative Mortality Risk (*RMR*) for Ages 60-79 and 80+ 11
Table S-6. RMR^{raw} Estimates 13

Table S-7. Multivariate Logit Results for *RMR* 14
Table S-8. Separate *RMR* Results for Men and Women 16
Figure S-1. Sample selection flowchart 17
Figure S-2. Correlation between Natural Mortality Rates in 2019 and COVID-19 Mortality Rates in 2020 18
Figure S-3. Indiana Natural Deaths in 2019 vs. Non-COVID Natural Deaths in 2020 19
Figure S-4. Vaccination Rates for Adults by Age Group in Indiana 20
Panel A. Full Vaccination Rates 20
Panel B. At least Three Dose Vaccination Rates 20
Panel C. At least Four Dose Vaccination Rates 21
Figure S-5. Actual versus Predicted NC-NMR During Pandemic Period 22
Panel A. Actual versus Predicted Non-COVID Natural Mortality 22
Panel B. Measured and Predicted Non-COVID Natural Deaths, with Confidence Intervals .. 22
Figure S-6. Raw vs. Interpolated Deaths for Vaccinated Persons 23
Panel A. Ages 15-59 23
Figure S-7. Raw versus Adjusted *RMR* 24
Panel A. Ages 15-59, Note. *RMR*=0: Complete Protection; *RMR*=1: No Protection. 24
Panel B. Ages 60+, Note. *RMR*=0: Complete Protection; *RMR*=1: No Protection. 24
Figure S-8. Healthy Vaccinee Bias for Finer Age Groups 25
Panel A. Ages 15-39 25
Panel B. Ages 40-59 25
Panel C. Ages 60-79 26
Panel D. Ages 80+ 26
Figure S-9. Relative Mortality Risk for Finer Age Groups 27
Panel A. Ages 15-39, Note. *RMR*=0: Complete Protection; *RMR*=1: No Protection. 27
Panel B. Ages 40-59, Note. *RMR*=0: Complete Protection; *RMR*=1: No Protection. 27
Panel C. Ages 60-79, Note. *RMR*=0: Complete Protection; *RMR*=1: No Protection. 28
Panel D. Ages 80+, Note. *RMR*=0: Complete Protection; *RMR*=1: No Protection. 28
Figure S-10. Plot of Vaccine Effectiveness Instead of *RMR* 29
Figure S-11. Relative Mortality Risk for Men vs. Women 30
Panel A. Ages 15-59 30
Panel B. Ages 60+ 30
Figure S-12. RMR^{raw} versus RMR^{CEMP} 31
Panel A. 1 or 2 doses 31
Panel B. 3 or 4 doses 31
Figure S-13. All-Cause Natural Mortality Relative to Vaccination Date 32
Figure S-14. *RMR* in Indiana vs. Milwaukee County 33
Panel A. *RMR* vaccinated vs unvaccinated: Ages 15-59 (IN) or 18-59 (Milwaukee) 33
Panel B. *RMR* 1 or 2 doses vs. 0: Ages 60+ 33

I. Additional Literature Discussion

There are many studies of vaccine effectiveness, some of which include death as an outcome. However, a number of factors, taken together, sharply limit the universe of studies that we viewed as sufficiently comparable to our own to be worth citing. We applied the following exclusion criteria: First, the US used Pfizer and Moderna almost exclusively, with a bit of J&J (see counts by vaccine type for our sample in Table S-2). In contrast, some European countries (including the UK) used Astra-Zeneca extensively; most did not use the J&J single-dose vaccine. We therefore consider only studies that provide either vaccine-specific results, or combined results for the two mRNA vaccines (Pfizer and Moderna).

Second, the core new results in this study concern 2Q-4Q 2022, after the peak of the Delta-Omicron wave of late 2021 to early 2022. When discussing results for 2022, we excluded studies with study periods that end before or during 1Q2022.

Third, some studies do not discuss results in calendar time, or report results using vaccine variant periods which map poorly onto calendar periods. For example, Fu et al. (2023) report results for an "Omicron period" from 24 December 2021 through June 30, 2022. These results will be numerically dominated by deaths in 1Q2022, and will not reflect the substantially lower *VE* we find beginning in 2Q2022.[1]

Fourth, some studies of booster doses cover only very short time periods after booster administration. Yet, we know that *VE* can wane, including against hospitalization and death, especially for the mRNA vaccines. The waning is progressive and becomes substantial after about 6 months (e.g., the review by Black and Thaw, 2024).

Fifth, some studies cover limited populations (e.g., persons in care homes; healthcare workers; college students, etc.). We consider only studies of the general population, plus studies of U.S. veterans, which have greater ability than other studies to control for individual health.

Sixth, some studies cover only persons tested for COVID-19 infection, persons already infected, or persons already hospitalized. Results from these studies cannot be compared to a population-based study such as ours because one effect of vaccination is to reduce the risk of infection or hospitalization. Seventh, some studies rely on a combined outcome of hospitalization or death. These do not provide good comparisons for our study, which is only of mortality. Eighth, given the existence of large-sample, country-level studies, studying populations in the millions, we excluded studies that used similar methods but had much smaller samples. Finally, we excluded studies from Hong Kong, Japan, South Korea, Australia, New Zealand, and other countries which suppressed COVID infections to near zero prior to the emergence of the Omicron variant, because their experience may not be comparable to the rest of the world.

As noted in the text, we found one study, of veterans during the Delta period, that found two-dose *RMR*s similar to those we report, for veterans seen at the VA, during 3Q-2021 (Delta period) (Cohn et al., 2022). This study controlled for age, sex, race, ethnicity, and comorbidities (Charlson comorbidity score, overweight, type II diabetes, chronic obstructive pulmonary disease, bronchitis, acute respiratory failure, and chronic lung disease). These controls should reduce

[1] We put aside Fu et al. (2023) for the further reason that their sample population is not clearly defined, and they report bizarre results, for example mostly negative *VE* against infection during the Omicron period, and negative J&J *VE* against death in all time periods.

selection bias, and may on the whole, provide an adjustment for selection effects comparable to our control for natural non-COVID deaths.

The other study we found which controls for health is Xu et al. (Sweden), which also found substantial waning against death. However, this study censors persons who received a booster dose. This can introduce selection bias, with regard to which people receive a booster dose. Selection bias likely explains why this study reports lowest two-dose *VE* (highest *RMR*) 38- 45 weeks after second dose, with *VE* then oddly rising after that.

Our effort to control for background mortality risk is similar in spirit to a UK study of how *VE* varies based on body-mass-index (BMI) (Piernas et al., 2022).

Cai et al. (2025), study U.S. veterans who received a booster in late 2024. They address healthy vacinee bias by comparing combined flu-COVID vaccine recipients with flu-vaccine-only recipients; both groups received a COVID booster in 2023. They report 28% *VE* over 6 months after vaccination for a composite outcome of COVID-19 infection, COVID-19-associated hospitalization, and all-cause mortality within 30 days of COVID infection. All-cause mortality fell 64% (95% CI 23%, 86%), but the mortality measure was not limited to persons who died of COVID-19 or were hospitalized for COVID-19. Our booster point estimates are within their CIs.

II. Indiana Population Estimates

We download mid-year, state-level population estimates from the U.S. Census Bureau, disaggregated by sex, year of age, race, and ethnicity.[2] We use the 2020 decennial Census to obtain population estimates as of July 1, 2020. The Census reports population counts for each single year of age from 0 to 84, with an aggregate category for individuals aged 85 and older. We aggregate the data to construct population counts by sex (male or female) and age (0–84 by year of age, and 85+). Total estimated population aged 15+ is 5,460,000 persons.

The accuracy of population estimates affect the accuracy of our estimates of HVBCF and RMR^{raw}, but do not affect $RMR^{CEMP.}$ The number of vaccinees is measured directly from vaccination records. However, the number of unvaccinated persons in age group a is derived as:

$$Unvax_a = Population_a - Vax_a$$

For older ages, most people are vaccinated, so a small percentage error in measuring population implies a larger percentage error in estimating the unvaccinated population, thus in measuring COVIDMR for unvaccinated persons, and thus in estimating $RMR^{raw}_{vax,unvax}$. Using RMR^{CEMP} as the outcome avoids this issue.

III. Details on Counting Vaccinated Persons and Number of Vaccine Doses

We obtained data on vaccinated individuals in Indiana, including date of each vaccine dose, and vaccine type for each dose from IHIE. The original IHIE dataset, before cleaning, includes 10,626,687 vaccine doses for 4,126,277 distinct individuals). We remove:

[2] Source: https://www2.census.gov/programs-surveys/popest/. Population estimates are available for six race categories, with an indicator for Hispanic origin. The race categories are: White Alone, Black or African American Alone, American Indian or Alaska Native Alone, Asian Alone, Native Hawaiian and Other Pacific Islander Alone, Two or more races.

(i) 780 duplicates, identified based on same IHIE identification number, same vaccination dates, and same vaccine manufacturers;
(ii) Persons aged < 15 at the date of first vaccination
(iii) Persons with first dose after Dec. 31, 2022.

Primary vaccination with the mRNA vaccines (from Moderna, mRNA1273; and Pfizer-BioNTech, BNT162b2) used two initial doses; the Johnson & Johnson (J&J) vaccine used one initial dose; the Novavax vaccine (approved mid-2022, minimally used in our sample) also used two initial doses. Standard U.S. two-dose timing was 28 days between doses for Moderna and 21 days for Pfizer. Mixing of vaccine types was uncommon (Table S-3).

We observe many cases where one person has an implausible number of vaccinations, often close together in time. Many of these appear to involve cases where one SID reflects the vaccinations for two separate people. We also see cases where two IHIE records have identical or very similar vaccination records, and are probably duplicate records. We adopt the following cleaning rules.

1. We drop one vaccination date before 14 December 2020, as recorded in error. This person (see details below) provides an example of common errors:

> The vax date of 18 Jan 2020 is obviously incorrect. The correct date appears to be 18 January 2021, which would be 3 weeks after the Pfizer dose on 28 December 2020. A vaccine dose is also recorded on 18 January 2021.
>
> This record could be for one person, who was vaccinated on 23 or 28 December 2020 and wrongly recorded as vaccinated twice 5 days apart, who received a second dose on 18 January 2021 (also recorded twice, once with a wrong date), and then a booster dose in November 2021.

IHIE ID	Vaccine date	Type	Dose number	Note
390815	18-Jan-20	Pfizer	1	Incorrect vax date
390815	23-Dec-20	Pfizer	2	
390815	28-Dec-20	Pfizer	3	
390815	18-Jan-21	Pfizer	4	
390815	5-Nov-21	Pfizer	5	

2. We drop 201 J&J vaccinations before the FDA's authorization of J&J on 26 February 2021.

3. We drop 88 Novavax vaccinations before the FDA's authorization of Novavax on 13 July 2022.

4. There is a small gap between the first two doses for many people, relative to the Pfizer minimum of 21 days and the Moderna minimum of 28 days. The number of second doses starts to climb for Pfizer shortly before a 21-day gap, and for Moderna shortly before a 28-day gap. The table below shows the gap between first and second doses in days. Cells with the minimum gap are in **boldface**.

First and second doses separated by 35 days or less

Days between doses	Pfizer	Moderna-	J&J	Novavax	Pfizer-Moderna or reverse	J&J-P/M
0					78	9
1	35	23	5		6	1
2	185	100	17		34	5
3	146	106	12		37	3
4	1363	855	169		246	50
5	1340	882	180		274	45
6	1440	680	146		256	56
7	2185	1119	208		431	96
8	1302	667	133		226	8
9	1134	509	110		220	44
10	1817	812	138		261	43
11	1161	494	79		162	346
12	1260	388	92		150	364
13	1039	391	56		180	368
14	1808	567	59		195	593
15	1158	396	52		149	322
16	1416	348	59		157	304
17	3899	424	47		128	369
18	4681	524	41	1	97	248
19	10,575	474	56		118	222
20	28,394	707	70	4	154	242
21	**1,197,534**	1606	60	48	**863**	295
22	204,584	759	49	15	232	229
23	119,523	1013	53	4	168	195
24	80,080	2341	45	3	163	182
25	65,273	3339	46		106	161
26	53,952	6671	40	1	116	194
27	46,603	17,194	31	3	133	234
28	121,772	**681,906**	47	11	**837**	1201
29	36,310	114,121	41	1	233	346
30	25340	69,267	41		166	252
Pfizer 0-17 days	22,688					
Moderna 1-24 days		16,185				
Mixed 1-17 days					3,112	

After reviewing this pattern, we adopt the following rules for the first two doses

(a) Drop the second dose if the gap is 0-17 days for Pfizer, J&J and mixed vaccine patterns;

(b) Drop the second dose if the gap is 0-24 days for Moderna.

5. *Third dose*. To count a dose as a third dose, we require it to be at least 120 days after the second dose and after the first date when the FDA allowed a third dose for the immunocompromised (Aug. 12, 2021) (this date restriction affects 1,336 people). Doses received earlier than this are dropped. The minimum allowed timing for a Pfizer or Moderna booster dose was 6 months, except for the immunocompromised. The minimum allowed timing for a second dose after a first J&J dose was 2 months, but that rule should not be relevant for the third dose.

6. *Third dose for immune compromised.* We keep the third dose for 43,358 people vaccinated between August 12, 2021, and FDA approval of the third dose for non-immunocompromised persons on Sept. 25, 2021, but treat receipt of a third dose during this period as evidence of immunocompromised status, and remove immunocompromised persons from the sample for our main analyses.

7. *Fourth dose:* To count a dose as a fourth dose, we require it to be at least 120 days after the third dose; and at least 120 days after the first approved date for the third dose (Aug. 12, 2021), thus after Dec. 10, 2021. Doses received earlier than this are dropped.

J&J Vaccinees. We measure *RMR* and *VE* for primary vaccination (either one or two doses). Mano of the one-dose recipients received J&J, for which one dose was considered to be full primary vaccination, but some received only one Pfizer or one Moderna dose (Table S-3). We treat J&J followed by an mRNA dose as 3 doses, thus treating the mRNA dose as a booster dose. We similarly treat J&J followed by two mRNA doses as 4 doses (thus, two booster doses).

Table S-3 provides summary data on vaccinated persons in our sample, how many doses they received, and which vaccine they received, after these cleaning steps, and after dropping the immune-compromised. Overall, through year-end 2022, around 70% of the sample received at least one dose.

Vaccine uptake was faster and more complete among those aged 60+, highest for ages 60-79, and slower and less complete at younger ages (Figure S-4). Three-dose percentages (conditional on receiving two doses) rise with age, but are similar for ages 60-79 and 80+.

The tendency for IHIE to sometimes combine two vaccinees into one record creates a small risk of bias, of uncertain sign. If two actual vaccinees are combined, we might either under- or over-estimate vaccinee mortality rates for vaccinated persons. If we underestimate vaccinee mortality rates, the bias will be similar to the bias, discussed in text and the next section, from undercounting vaccinated decedents during August-October 2021. If we overestimate vaccinee mortality rates, the bias will have the opposite sign. Fortunately, the percentage of combined vaccination records appears to be is small, around 1-2%, so any bias should also be small.

IV. Adjusting for Undercounting of Vaccinated Decedents

We define natural deaths as those with manner of death recorded as natural on the death certificate; the vast majority of decedents (91.39%) have natural manner of death. We exclude the remaining manner-of-death categories (accident, homicide, and suicide; together 8.04% of all deaths), and pending and undetermined (together, 0.57%).

We obtain IHIE data on vaccinees, number of vaccine doses, which vaccinees have died, and cause of death for the decedents. We clean the number of vaccine doses, as described above. We also have Indiana Vital Statistics (VS) records on all decedents.

A. Adjustment for IHIE Undercount of Vaccinated Decedents

We observe failure of IHIE to fully match VS death records to vaccination records in August-October 2021. Figure S-6 shows vaccinated decedents by month (from all natural causes) in the IHIE linked vaccination-mortality records, separately for ages 15-59 and 60+. We expect the number of vaccinated decedents to rise rapidly during 2021,once vaccines are available, as more people become vaccinated, and thus can potentially die while vaccinated. This is observed in general, but with a visually obvious drop in August-October 2021 in the number of vaccinated

decedents. We asked IHIE personnel about the source of this dip. They recognized the issue, but had no solution to offer. The undercount of vaccinated decedents implies a corresponding overcount of unvaccinated decedents during 3Q2021 and 4Q2021. This can produce downward bias in RMR^{CEMP} estimates.

We address the undercount, and thus the bias in RMR^{CEMP}, as follows. First, we interpolate from July 2021 to November 2021, to obtain adjusted counts of vaccinated decedents. We then increase the monthly numbers of *adjusted* vaccinated deaths so that it follows the interpolation line, and correspondingly decrease the numbers of *adjusted* unvaccinated deaths.

We assume that the missing deaths of vaccinated persons reflect random matching error, and is unrelated to whether the death was due to COVID or not. With this assumption, which we view as reasonable, the adjustment to vaccinated deaths will not affect $CEMP_{vax}$.

To compute *adjusted* $CEMP_{unvax}$, we proceed as follows. We first used the measured $CEMP_{vax}$ to attribute the extra interpolated deaths among vaccinated persons to COVID versus other natural causes. We then remove the same numbers of COVID and natural, non-COVID deaths from unvaccinated persons and recompute $CEMP_{unvax}$.

To use a numerical example, assume that interpolation increases the number of vaccinated deaths, for a given age group in a given quarter, by 100, and that $CEMP_{vax}$ is 25%. Then, of the 100 additional vaccinated deaths, 20 should be COVID and 80 should be non-COVID (20/80 = 0.25). We therefore remove 20 COVID deaths and 80 non-COVID deaths from the unvaccinated population, and compute *adjusted* $CEMP_{unvax}$. This will be *higher* than unadjusted raw $CEMP_{unvax}$ because the removed deaths are disproportionately non-COVID deaths. This, in turn, will *reduce* $RMR_{x,unvax}^{CEMP} = \frac{CEMP_{vax}}{CEMP_{unvax}}$. Unadjusted $RMR_{x,unvax}^{CEMP}$ was biased upwards.

We compute adjusted $CEMP_{unvax,a,t}$ separately by vaccination status, age group, and quarter, for 3Q and 4Q 2021, separately for the age ranges used in the text (15-59 and 60+) and also for the finer age ranges used in Tables App-4 and App-5 (15-39, 40-59, 60-79, 80+. The examples below use age ranges 15-59 and 60+.

The table below provides the steps in our calculation of adjusted counts of vaccinated and unvaccinated decedents. We compute missing vaccinated decedents by month, and roll up to quarter.

Table Interp-1: Original and Interpolated Counts of COVID and other Natural Deaths

Table shows original IHIE counts of vaccinated ("vax") decedents from natural causes, who received 1+ vaccine doses, and interpolated counts for 3Q and 4Q2021. Deaths are counted from 30 days after receipt of first vaccine dose. Interpolated amounts are rounded to 2 decimal places. *CEMP* is shown as a fraction rather than a percentage. Sample excludes immune-compromised persons.

**Age**	**Formulas**	**15-59**	**15-59**	**60+**	**60+**
Quarter		**3Q2021**	**4Q2021**	**3Q2021**	**4Q2021**
Observed vax deaths	(1)	562	949	6101	9079
Interpolated vax deaths	(2)	728.25	980.25	7991.25	9632.25
Observed vax COVID deaths	(3)	28	65	255	759
Observed vax non-COVID deaths	(4)	534	884	5846	8320
Observed $CEMP_{vax}$	$(5) = \frac{(3)}{(4)}$	0.052	0.074	0.044	0.091
Interpolated COVID deaths for vaccinated	$(6) = (2) * [\frac{(5)}{1+(5)}]$	36.28	67.14	334.01	805.25
Interpolated non-COVID deaths for vaccinated	(7) = (2) – (6)	691.97	913.11	7,657.24	8,827

Extra observed COVID deaths for **unvaccinated**	(8) = (6)-(3)	8.28	2.14	79.01	46.25
Extra observed non-COVID deaths for **unvaccinated**	(9) = (7) – (4)	157.97	29.11	1811.24	507.00
Observed unvax COVID deaths	(10)	446	676	1100	1865
Adjusted unvax COVID deaths	(11) = (10) – (8)	437.72	673.86	1020.99	1818.75
Observed unvax non-COVID deaths	(12)	1559	1343	7224	5754
Adjusted unvax non-COVID deaths	(13) = (12) – (9)	1401.03	1313.89	5412.76	5247.00
Observed $CEMP_{\text{unvax}}$	(14) = $\frac{(10)}{(12)}$	0.29	0.50	0.15	0.32
Adjusted $CEMP_{\text{unvax}}$	(14) = $\frac{(11)}{(13)}$	0.31	0.51	0.19	0.35
Observed $RMR_{vax,unvax}^{CEMP}$		0.1833	0.1461	0.2865	0.2815
Adjusted $RMR_{vax,unvax}^{CEMP}$		0.1678	0.1434	0.2312	0.2632
Weights for vax for COVID deaths		1.2958	1.0329	1.3098	1.0609
Weights for vax for non-COVID deaths		1.2958	1.0329	1.3098	1.0609
Weights for unvax for COVID deaths		0.9814	0.9968	0.9282	0.9752
Weights for unvax for non-COVID deaths		0.8987	0.9783	0.7493	0.9119

The last step is to compute adjusted *RMR* for different numbers of doses x, based on observed $CEMP_x$ and adjusted $CEMP_{\text{unvax}}$.

$$RMR_{x,unvax}^{CEMP,adj} = \frac{CEMP_x}{CEMP_{unvax}^{adj}}$$

B. Estimating Confidence Intervals for Different Numbers of Vaccine Doses

To estimate confidence intervals for $RMR_{x,unvax}^{CEMP,adj}$, we apply weighted logistic regression within each broad age group. We calculate weights as the ratio of interpolated to observed counts for both COVID and non-COVID deaths. The weights depend on the numbers of doses x. The weight construction is shown below, using the 1or2 dose category as an example.

Table Interp-2: Original and Interpolated Counts: 1 or 2 Dose Vaccinees

Table shows original IHIE counts of decedents from natural causes, who received 1 or 2 vaccine doses, and interpolated counts for 3Q and 4Q2021. Deaths are counted from 30 days after receipt of first vaccine dose. Interpolated amounts are rounded to 2 decimal places. *CEMP* is shown as a fraction rather than a percentage. Sample excludes immune-compromised persons.

**Age**	**Formulas**	**15-59**	**15-59**	**60+**	**60+**
Quarter		**3Q2021**	**4Q2021**	**3Q2021**	**4Q2021**
Observed deaths, 1or 2 doses	(1)	562	928	6101	8167
Interpolated deaths, 1or 2 doses	(2)	725.25	956.25	7857	8592
Observed COVID deaths, 1or 2 doses	(3)	28	65	255	728
Observed non-COVID deaths, 1or 2 doses	(4)	534	863	5846	7439
Observed $CEMP_{1or2}$	$(5) = \frac{(3)}{(4)}$	0.052	0.075	0.044	0.098
Interpolated COVID deaths for vaccinated	$(6) = (2) * [\frac{(5)}{1+(5)}]$	36.13	66.98	328.39	765.88
Interpolated non-COVID deaths for vaccinated	(7) = (2) – (6)	689.12	889.27	7528.61	7826.12
Weights for 1-2 doses for COVID deaths		1.2905	1.0304	1.2878	1.0520
Weights for 1-2 doses for non-COVID deaths		1.2905	1.0304	1.2878	1.0520
Observed $RMR^{CEMP}_{1or2,unvax}$		0.1833	0.1496	0.2865	0.3019
Adjusted $RMR^{CEMP}_{1or2,unvax}$		0.1678	0.1469	0.2312	0.2823

C. No Adjustment for Booster Recipients

For persons receiving booster doses, we do not compute adjusted deaths, adjusted $CEMP_{unvax}$ or adjusted *RMR*. Recall that booster approval came only on Sept. 24, 2021, approval was limited at that time to persons aged 65+ or who fell within defined high-COVID-risk categories, we count deaths as attributable to a vaccine group starting 30 days after vaccination, and that the need for interpolation exists only for August-October 2021. There is no need for interpolation for 3Q2021, and minimal need for October 2021 and thus for 4Q2021. There are very few booster recipients who are eligible to be counted as booster recipient decedents by the end of October. There are zero observed COVID-19 deaths among 3-dose recipients aged 15-59 in 4Q2021. Thus, adjusted *RMR* for this age group is meaningless; unadjusted *RMR* is already 0. For 3-dose recipients aged 60+, we observe 30 COVID deaths over 4Q 2021.

V. Identifying COVID-19 Decedents from Cause of Death Fields

The death certificates that we rely on include text fields for primary cause of death, other conditions in the causal chain, and other significant conditions. We conduct text analysis of these fields to determine which deaths are likely to be caused by COVID-19. Table S-1 compares the COVID-19 counts we determine using text analysis to those from the ICD-10 codes included in death records, which are generated by personnel at the National Center for Health Statistics (NCHS) based on the text fields. Our text analysis algorithm was developed and substantially completed before we had access to the ICD-10 codes. Our goal was to develop a reasonable method for estimating COVID-19 deaths, based on the text fields in death certificates. The principal decisionmakers for this approach were Drs. John Meurer and Jeff Whittle. The total counts of COVID-19 deaths are very similar using both approaches.

In Figure S-3, we report evidence for Indiana on the correlation between natural deaths in 2019 (pre-COVID period) and measured non-COVID natural deaths in 2020, using the text-based measure. The Pearson correlation coefficient is 0.999. These high correlations provide further evidence that the text-based measure does a good job of capturing COVID-19 deaths.

Another possibility, on which Figure S-3 also provides evidence, is that prior COVID-19 infection will lead to higher future deaths from natural causes, not directly linkable to the prior infection. Higher post-infection mortality would predict positive actual-minus-predicted non-COVID-19 natural mortality generally, not limited to periods of high COVID-19 mortality. There is evidence from other research of excess cardiovascular risk for a limited period of time following infection.[3] However, the extents of excess deaths is not known. Any excess deaths appear, from our Indiana data, not to be at levels apparent from the figure.

The detailed coding we used to identify deaths as due to or probably due to COVID-19 is available from the authors on request. In brief, we counted as COVID-19 deaths those for which:

(i) COVID-19 or variants (such as SARS-2, SARS-COV-2, coronavirus) was listed as the principal cause of death; or

(ii) COVID-19 or variants were listed in the causal chain and the principal cause of death was likely to be caused by COVID-19 (for example, pneumonia, respiratory failure, hypoxia); or

(iii) COVID-19 or variants were listed in the causal chain and the principal cause of death was a known potential outcome of COVID-19 infection (for example, heart attack, stroke, sepsis) or involved underlying disease that was plausibly exacerbated by COVID-19 infection (for example, heart failure, COPD, dementia); or

(iv) COVID-19 was included in the "other significant conditions" field, but not in the causal chain fields, and (a) the principal cause of death was likely to be caused by COVID (group (ii) above); and (b) there was no entry in the causal chain fields indicating another likely cause of death.

We view reliability as high for categories (i) and (ii), and moderate for categories (iii) and (iv). The false positives (COVID-19 as principal cause of death based on ICD-10 codes, but not on our text-based coding) involve deaths for which the text fields do not fit in any of these four categories.

VI. Evidence on Excess Natural Non-COVID Deaths

We discuss in this section evidence on the accuracy of the COVID-19 death counts. To the extent that our counts of COVID-19 deaths undercount actual COVID-19 deaths, this should show up in measures of excess non-COVID natural deaths, defined as non-COVID-19 natural deaths, minus predicted levels based on extrapolation of mortality trends from the pre-pandemic period. Undercounting COVID-19 deaths would lead to excess deaths being positive during the pandemic periods, and spiking during the periods when measured COVID-19 mortality spikes. In Figure S-5, Panel A, we show monthly measured natural deaths (including COVID-19 deaths), measured natural non-COVID-19 deaths , and a prediction line based on the predicted values from separate regressions for each calendar month (to allow for seasonality in natural deaths) of an indicator for the year on the number of deaths from the pre-COVID-19 period of 2017-2019. Natural non-COVID-19 deaths had much smaller spikes, with magnitudes consistent with the usual tendency for mortality to rise in the winter. Predicted natural non-COVID natural deaths (dashed line) were close to measured non-COVID natural deaths, but often somewhat higher, suggesting a moderate number of excess non-COVID-19 natural deaths.

[3] Rezel-Potts et. al (2022).

Panel B zooms in on the pandemic period, drops the line for all natural deaths, and shows the 95% confidence interval (CI) around predicted deaths. The measured count is above the top of the CI for the predicted count in the second half of 2020 and the second half of 2021. However, the measured count is below the bottom of the CI for the predicted count for some months during the first half of 2021 and 2022.

Nationally, there is evidence for undercounting of COVID-19 deaths, including from our own work.[4] We find evidence of some undercounting in Indiana, but not at particularly severe levels.

The measured count of natural non-COVID deaths falls below the bottom of the CI following the large COVID death spike in late-2020 and again following the late-2021-to-early-2022 COVID-19 spike. Recent COVID-19 infection is known to predict somewhat higher risk for some causes of death in the next several months.[5] The comparison of actual to predicted non-COVID natural deaths suggests that a more important effect may be for periods with high COVID-19 deaths to accelerate death for some people who would have died in the near future anyway.

VII. Virus Variants and Periods of Dominance

Accounting for the two-week lag we impose between dose administration and assumed effectiveness, the Alpha strain was dominant in the U.S. starting the week of March 14, 2021; the Delta variant was dominant beginning the week of July 4, 2021, and the Omicron variant has been dominant starting the week of Jan. 2, 2022.[6] These periods correspond reasonably closely to the calendar quarters we use in text.

VIII. Measuring Population by Number of Vaccine Doses

We construct a synthetic Indiana population by age-group and month as follows. We begin with the U.S. Census Bureau, disaggregated by sex, year of age, race, and ethnicity, for 2020, 2021 and 2022 which we assume apply to January of each year. State-level population is available at single year of age from 0 to 84, and then a single count for persons 85+.

To get monthly counts of vaccinated and unvaccinated persons, we measure the number of people vaccinated with a given number of doses x by first moving the vaccination date forward 30 days (to allow for lags from vaccination to vaccine effectiveness and from infection to death). Vaccine doses are generally understood to become fully effective over a 7-14 day period after vaccination. Other studies suggest a mean of 18 days from symptom onset (itself a few days after infection) to death for COVID-19 decedents.[7] The 30-day lag captures both periods.

We then count the number of vaccinated persons as of the first day of each month (counting persons as vaccinated in month m if they were vaccinated during the first 15 days of that month, and as vaccinated in month m+1 if vaccinated later in month m. To obtain monthly counts of the

---

[4] Rossi et al. (2025).

[5] Xie et. al (2022).

[6] Source: https://www.gisaid.org/; see also https://covid.cdc.gov/covid-data-tracker/?utm_source=STAT+Newsletters&utm_campaign=059492f101-MR_COPY_01&utm_medium=email&utm_term=0_8cab1d7961-059492f101-153972538#variant-proportions

[7] Yang et. al (2020), Marschner (2021).

unvaccinated, we subtract the counts of vaccinated persons from the Census population for the same age group.

Failure to allow a sufficient period between vaccine administration and death can lead to misleading results. An extreme example is a U.K. study which reported 98.7% *VE* for a Pfizer booster against death [CI 97.4, 99.4].[8] However, this study measured death only over 14-34 days after booster receipt.

In unreported results we find slightly higher *RMR* if we treat vaccination as effective against vaccination after 14 days instead of 30 days.

IX. Time Period Studied, Variable Definitions, and Other Methods Details

COVID-19 vaccines because available in the U.S. in early 2021, but initially with limited supply, generally the elderly, other persons at high risk for severe COVID-19, healthcare workers, and other persons in high-exposure occupations. Availability expanded greatly in April 2021 and vaccines became available to all who wanted them by May 2021.

Table S-2 summarizes vaccine approval dates. Boosters were first authorized by the Centers for Disease Control and Prevention (CDC) on September 24, 2021, initially for ages 60+ and healthcare workers and others in high -risk occupations. The CDC authorized boosters for all adults on November 19, 2021, but recommended them only for ages 50+.[9] On November 29, 2021, the CDC recommended boosters for all adults.[10]

A. *CEMP*, *VE*, and *RMR*

The formulas in this section generalize those in the text to any comparison of people with x versus y vaccine doses (y = 0 for the unvaccinated). We define the COVID-19 Excess Mortality Percentage (*CEMP*), for persons with x vaccine doses, vaccine effectiveness for x doses versus a base number of y doses ($VE_{x,y}$), and relative mortality risk after vaccination with x versus y doses ($RMR_{x,y}$) in time period *t*, within age group *a*, as:

$$CEMP_x = 100 * \frac{COVID\ deaths_x}{non\text{-}COVID\ natural\ deaths_x} \quad (1a)$$

$$RMR_{x,unvax}^{CEMP} = \frac{CEMP_x}{CEMP_{unvax}} \quad (1b)$$

$$VE_{x,unvax}^{CEMP} = 1 - RMR_{x,unvax}^{CEMP} \quad (1c)$$

We study primary vaccination (one or two doses) in each time period, and three-dose and four-dose vaccinees for the periods when a booster dose was available. By using non-COVID natural deaths in the *CEMP* denominator, we treat the non-COVID natural mortality rate as a proxy for the overall health of a given group, which can control for selection effects, including who gets vaccinated, when, with which vaccine, and with how many doses. Because the sample is

8 Andrews et. al (2022).

9 CDC Press Release (Nov. 19, 2021); Mandavilli (2021).

10 CDC Press Release (Nov. 29, 2021).

decedents, these estimates are, in effect, mortality-weighted. Thus, results for a broad age group give primary weight to older persons within that group.

We count a person as vaccinated with x doses beginning 30 days after the x'th dose.

$RMR_{x,y,a,t}$ can be obtained by comparing mortality rates for two groups, as in eqn.. (1c), or as an odds ratio from logistic regression for a population containing both groups.

CEMP, *VE*, and *RMR* involve ratios, so will be undefined if the denominator is zero. We found zero values for $CEMP_{y,a,t}$ in some situations for later time periods, generally for larger base number of doses y, and younger population group, and thus have some undefined *VE* and *RMR* values.

It is also useful to define a measure of healthy vaccinee bias (*HVB*), for persons with x vaccine doses relative to a base number of y doses, in time period t for age group a. Let Non-COVID-*NMR* (or *NCNMR*) be the non-COVID natural mortality rate:

$$NCNMR_{x,a,t} = \frac{non - COVID\ natural\ deaths_{x,a,t}}{Pop_{x,a,t}}$$

We measure the denominator for quarter t as the average of the population at the beginning of quarter t and quarter t+1.

We can then define a healthy vaccinee bias correction factor:

$$HCBCF_{x,y,a,t} = \frac{NCNMR_{x,a,t}}{NCNMR_{y,a,t}}$$

Healthy vaccinee bias, in which people with more vaccine doses are healthier than people with fewer doses, and have thus have lower *NCNMR* rates, implies HVBCF values less than one. The central assumption for this measure of healthy vaccinee bias is that COVID-19 vaccination does not affect non-COVID natural mortality.

Now also define an RMR^{raw} measure, which is based on raw COVID-19 mortality rates (*COVIDMR*), rather than on *CEMP*, and thus does not use *NCNMR* rates to address healthy vaccinee bias.

$$RMR^{raw}_{x,y,a,t} = \frac{COVIDMR_{x,a,t}}{COVIDMR_{y,a,t}}$$

There is an interesting relationship between the HVBCF, *RMR* (based on *CEMP*) and RMR^{raw}. Multiplying $RMR_{x,y,a,t}$ by $HVBCF_{x,y}$ we get:

$$RMR_{x,y,a,t} * HVBCF_{x,y,a,t} = \left(\frac{\dfrac{COVID\ deaths_{x,a,t}}{non - COVID\ natural\ deaths_{x,a,t}}}{\dfrac{COVID\ deaths_{y,a,t}}{non - COVID\ natural\ deaths_{y,a,t}}} \right) \left(\frac{\dfrac{non - COVID\ natural\ deaths_{x,a,t}}{Pop_{x,a,t}}}{\dfrac{non - COVID\ natural\ deaths_{y,a,t}}{Pop_{y,a,t}}} \right)$$

Simplifying:

$$RMR_{x,y,a,t} * HVBCF_{x,y,a,t} = \frac{\frac{COVID\ deaths_{x,a,t}}{Pop_{x,a,t}}}{\frac{COVID\ deaths_{y,a,t}}{Pop_{y,a,t}}} = RMR^{raw}_{x,y,a,t},$$

Rearranging:

$$RMR_{x,y,a,t}(based\ on\ CEMP) = \frac{RMR^{raw}_{x,y,a,t}}{HVBCF_{x,y,a,t}}$$

Thus, for example, if RMR^{raw} = 0.10 (90% VE^{raw}) and HVBCF is 0.5 (vaccinees have half the background mortality risk of the unvaccinated), then RMR^{CEMP} will be $RMR^{CEMP} = \frac{0.1}{0.5} = 0.20$.

B. Univariate Logistic Regression and Confidence Intervals

CEMP represents the odds, for a sample consisting of natural cause decedents, of dying from COVID-19 versus dying other natural causes, converted to a percentage.

$$CEMP = 100* \frac{\sum COV_i^{mort}=1}{\sum COV_i^{mort}=0}$$

Here COV_i^{mort} equals 1 for COVID-19 decedents, 0 for decedents from other natural causes.

This means that *CEMP* can be measured either directly, as in eqn. (1a), or through logistic regression. The logistic regression lets us obtain confidence intervals around *CEMP* point estimates. We run separate logistic regressions by calendar quarter and age group using individual-level death records from Indiana Vital Statistics. The sample is limited to decedents from natural causes, excluding immune-compromised persons. The outcome variable is an indicator equal to one for COVID-19 death; zero if it was a non-COVID natural death. The main regressor of interest is vaccination status at time of death, represented by mutually exclusive indicator variables for each dose group (I_g). Unvaccinated persons is the omitted reference category.

For each quarter *q* and age bin *a*, we estimate the following logistic regression:

$$\log(\frac{\Pr[COVID_i = 1 \mid dose_i = g]}{\Pr[COVID_i = 0 \mid dose_i = g]}) = \alpha + \beta_g * I_g + \varepsilon_i$$

for each vaccination group $g \neq 0$,, where $dose_i = g$ indicates individual *i*'s vaccination status, and group $g = 0$ (unvaccinated) is the reference category. Here:

- The left-hand side is the log-odds of a COVID death relative to a non-COVID natural death.
- β_g is the log of the odds ratio for group g relative to the unvaccinated group.
- Exponentiating this coefficient gives the odds ratio:

$$RMR^{CEMP}_{g,0} = \exp(\beta_g)$$

In our context, this odds ratio corresponds to *RMR* defined as the ratio of the COVID-to-non-COVID death ratio (i.e., *CEMP*) in group g relative to the same ratio in the unvaccinated group:

$$RMR_{g,0}^{CEMP} = \frac{CEMP_g}{CEMP_0} = \frac{\frac{COVID\ deaths_g}{nonCOVID\ deaths_g}}{\frac{COVID\ deaths_0}{nonCOVID\ deaths_0}}$$

This equivalence between the exponentiated regression coefficient and the algebraic expression for *RMR* arises because the regression is estimated only among decedents, and the model compares the odds of COVID versus non-COVID natural death within each vaccination group. We verify that the numerical values for *CEMP* and RMR^{CEMP}, obtained from the logistic regression above, exactly match values computed algebraically using death counts.

Confidence Intervals for RMR^{CEMP}. To construct 95% confidence intervals for RMR^{CEMP}, we exponentiate the lower and upper bounds of the confidence interval for the log-odds coefficient $\widehat{\beta}_g$, using robust standard errors.

$$CI_{RMR_g} = [\exp\left(\hat{\beta}_g - 1.96 * SE(\hat{\beta}_g)\right), \exp\left(\hat{\beta}_g + 1.96 * SE(\hat{\beta}_g)\right)]$$

The confidence interval is asymmetric around the *RMR* point estimate because the exponential function is nonlinear.

C. Multivariate Logistic Regression

As a robustness check, we estimate a fully adjusted multivariate logistic regression model that controls for a broad set of individual-level covariates. We estimate the following regressions separately for each calendar quarter and age bin.

$$\log(\frac{\Pr[COVID_i = 1 \mid dose_i = g]}{\Pr[COVID_i = 0 \mid dose_i = g]}) = \alpha + \beta_g * I_g + f(age_i) + \boldsymbol{\gamma}'\boldsymbol{X_i} + \varepsilon_i$$

where $f(age_i)$ is a quadratic in age, and $\boldsymbol{X_i}$ includes categorical indicators for sex, race, education, marital status, and quintiles of the Graham Social Deprivation Index, as a measure of area-SES. As in the univariate specification, the exponentiated coefficients on the dose indicators yield estimates of *RMR* by vaccination status, and we construct CIs by exponentiating the log-odds confidence bounds. See Table S-7 for the multivariate estimates. These estimates are consistent with the univariate estimates in text and confirm that the observed univariate RMR^{CEMP} estimates are robust to controlling for additional individual characteristics.

D. Confidence Intervals for HVBCF

To obtain 95% confidence intervals for HVBCF for persons with x versus y vaccine doses, we use the log-transformation method, and use the delta method for standard errors (Kirkwood and Sterne, 2003, p. 253):

$$SE(\log(HVBCF_{x,y,a,t})) = \sqrt{(\frac{1}{D_{xat}} + \frac{1}{D_{yat}})}$$

where $D_{x,a,t}$ and $D_{y,a,t}$ are the number of natural non-COVID deaths among persons with x and y vaccine doses, in age group a and time period t. To obtain 95% confidence intervals, we use the following formula:

$$CI_{HVB_{x,y,a,t}} = \left[\frac{HVBCF_{x,y,a,t}}{M}, HVBCF_{x,y,a,t} \times M\right], where\ M = \exp\left[1.96 * SE\left(log(HVBCF_{x,y,a,t})\right)\right]$$

X. Validating the *CEMP* Measure

CEMP uses *NCNMR* to proxy for overall health of a given group, and thus the likelihood of mortality if not vaccinated. We discuss validation in text, and provide additional results in Figure S-2. This figure compares natural mortality *rates* in April–December 2019 (pre-COVID) to COVID-19 mortality rates over April–December 2020. In contrast, text Figure 1 compares *counts*.

Using 2019 natural mortality (rather than 2020 non-COVID natural mortality) to predict 2020 COVID mortality avoids the mechanical correlation which could arise if COVID deaths are undercounted or concurrent or prior COVID infection leads to higher non-COVID natural mortality.

In Figure S-3 we confirm that all-cause natural mortality in Indiana in 2019 is highly correlated with *NCNMR* in 2020 (r = 0.999). We find similarly strong correlations using Wisconsin data in our prior work (Atanasov et al., 2023a).

XI. Additional Results for *RMR*, *HVBCF*, and *VE*

Table S-4, Panel A, provides numerical values underlying the HVBCF graphs in text Figure 2. Panels B and C provide numerical values for finer age groups: 15-39; 40-59; 60-79; and 80+. Figure S-8 provides graphs for the finer age groups.

Table S-5, Panel A, provides numerical values underlying the HVBCF graphs in text Figure 3. Panels B and C provide numerical values for finer age groups: 15-39; 40-59; 60-79; and 80+. Figure S-9 provides graphs for the finer age groups.

Much of the published research on COVID-19 reports vaccine effectiveness (*VE*) rather than *RMR*. In Figure S-10, we convert the results for RMR^{CEMP} in text Figure 3 to results for VE^{CEMP}, for easier comparison to other vaccine effectiveness studies.

XII. Effects of Vaccination-Avoidance by the Very Ill

One concern with our measure of healthy vaccinee bias is the tendency for very ill people, including those with terminal illness, not to get vaccinated. This tendency implies that: (i) $NCNMR_{vax}$ measured soon after vaccination could be higher than if measured some time after vaccination; (ii) $CEMP_{vax}$ measured soon after vaccination could be higher than if measured some time after vaccination; and (iii) healthy vaccinee bias measured soon after vaccination could be lower than if measured some time after vaccination. This could lead to near-term $RMR^{CEMP}_{x,unvax}$ estimates being lower than if measured some time after vaccination.

In Figure S-13, we assess for how long this effect lasts, following the most recent vaccination date. We plot daily, all-cause natural mortality as a function of days since vaccination, for the 1st, 2nd, 3rd, and 4th dose. In all four graphs there is an upward trend soon after vaccination, which lasts for around 30 days after the vaccination date.

Recall, however, that we count vaccination as effective against mortality beginning 30 days after vaccination. Figure S-13 suggests that this delay period is sufficient to address this potential source of bias in soon-after-vaccination estimates of RMR^{CEMP}.

XIII. Defining the Immune-Compromised

In the results in text, we exclude persons known to be immune-compromised, defined as: (i) decedents whose death records indicate a solid organ transplant; (ii) decedents whose death records state that they were immune-compromised; and (iii) persons who received a third vaccine dose on or before September 24, 2022, the first date when a third dose was authorized for the non-immune-compromised. The standard CDC recommendation for full vaccination included a third "primary" dose; for these persons, the fourth dose was the first dose considered to be a booster dose. The booster studies by Arbel et al. (2021) and Bar-On et al. (2021) also exclude early third-dose recipients.

XIV. Raw *RMR* (Using COVID-19 Mortality Rates Rather than *CEMP*)

Figure S-11 compares our results for *RMR* to provides results for RMR^{raw}, measured using the COVID-19 Mortality Rate (CovidMR) as the outcome instead of *CEMP*, and thus measuring *RMR* without controlling for selection effects. The RMR^{raw} levels s are comparable to *RMR* values from other studies, which do not control for these selection effects. As expected, given the selection effects shown in text table 3, RMR^{raw} values are substantially lower than those reported in the text and continue to show substantial vaccine protection over 2Q-4Q2022.

XV. *RMR* in Indiana versus Milwaukee County, Wisconsin

As a robustness check on our *RMR* results for Indiana, we also computed *RMR* using the Milwaukee County, Wisconsin (MKE) dataset that we used in prior work.[11] We extended the sample period to include 1Q2021-4Q2022. Iin our prior work, we had studied 2Q2-21-2Q2022, but combined data for 1Q and 2Q2022. We compare *RMR* for each sample in Figure S-12.

For ages 15-59, putting aside quarters with 0 *RMR* in MKE (due to no COVID-19 deaths), *RMR* point estimates are similar in both samples. For ages 60+, where most COVID-19 deaths occur and estimates are more precise, CIs from the two areas overlap and point estimates are quite close through 1Q2022. After that the MKE point estimates are lower, but the MKE CIs include the Indiana point estimates, indicating that the differences between the two samples are not statistically significant.

For younger persons, the CIs again overlap, and point estimates are similar through 1Q2022. After that, COVID-19 deaths too uncommon in MKE, with its smaller population, to say much. There are no COVID-19 deaths in 2Q2022 or 4Q2022, and only one death in 3Q2022.

RMR point estimates for a combined sample would be dominated by the much larger Indiana sample and would be close to the Indiana estimates.

XVI. Mortality-Weighted versus Population-Weighted Results

The overall *CEMP* and RMR^{CEMP} estimates for broad age groups, presented in the text and this Supplemental Material, are effectively weighted by the number of natural, non-COVID

[11] Atanasov et al. (2023).

decedents in each cell. This weighting feels appropriate to us, because it weights RMR^{CEMP} toward the people who are most affected by COVID-19. However, one could also weight RMR^{CEMP} estimates by population. This approach is typical for other studies. However, the RMR^{CEMP} estimates we report do not depend strongly on age, so population weighting would not significantly change those estimates; which we confirm in unreported results.

Additional References for supplementary materials (not cited in the text)

Fu, Yuanyuan, Kaipeng Wu, Zhanwei Wang, Hua Yang, Yu Chen, Lang Wu, Richard Yanagihara, Jerris R Hedges, Hongwei Wang, Youping Deng, and N3C consortium (2023), Effectiveness of various COVID-19 vaccine regimens among 10.4 million patients from the National COVID Cohort Collaborative during Pre-Delta to Omicron periods – United States, 11 December 2020 to 30 June 2022, *Vaccine*41(42): 6339-6349.

Griffin, Jennifer B, Meredith Haddix, Phoebe Danza, Rebecca Fisher, Tae Hee Koo, Elizabeth Traub, Prabhu Gounder, Claire Jarashow, and Sharon Balter (2021), SARS-CoV-2 Infections and Hospitalizations Among Persons Aged ≥16 Years, by Vaccination Status — Los Angeles County, California, May 1–July 25, 2021, *Morbidity and Mortality Weekly Report* 70(34), 1170-1176.

Kirkwood, Betty R., and Jonathan A.C. Sterne, *Essential Medical Statistics* (2003).

Mandavilli, Apoorva (2021), C.D.C. Endorses Covid Vaccine Booster Shots for Al Adults, *New York Times* (Nov. 19).

Marschner, Ian C. (2021). Estimating age-specific COVID-19 fatality risk and time to death by comparing population diagnosis and death patterns: Australian data, *BMC Medical Research Methodology* 21: 126.

Piernas, Carmen, Martina Patone, Nerys M Astbury, Min Gao, Aziz Sheikh, Kamlesh Khunti, Manu Shankar-Hari, Sharon Dixon, Carol Coupland, Paul Aveyard, Julia Hippisley-Cox*, and Susan A Jebb (2022), Associations of BMI with COVID-19 vaccine uptake, vaccine effectiveness, and risk of severe COVID-19 outcomes after vaccination in England: a population-based cohort study, *Lancet Diabetes and Endocrinology* at https://doi.org/10.1016/ S2213-8587(22)00158-9.

Rezel-Potts, Emma, Abdel Douiri, Xiaohui Sun, Phillip J. Chowienczyk, Ajay M. Shah, and Martin C. Gulliford (2022), Cardiometabolic outcomes up to 12 months after COVID-19 infection. A matched cohort study in the UK, *PLoS*, https://doi.org/10.1371/journal.pmed.1004052.

Rossi, Francesco, Lorenzo Franchi, Vladimir Atanasov, Natalia Barreto, Beatrice Nardone, Jeff Whittle, John Meurer, Qian (Eric) Luo, Andy (Ye) Yuan, and Bernard Black, *Life-Expectancy Loss During the COVID-19 Pandemic: Decomposition Using Individual-Level Mortality Data* (working paper 2025), at http://ssrn.com/abstract=3795801.

Yang, Xiaobo, Yuan Yu, Jioqian Xu, Huaqing Shu, Jia'an Xia*, Hong Liu, Yongran Wu, Lu Zhang, Zhui Yu, Minghao Fang, Ting Yu, Yaxin Wang, Shangwen Pan, Xiaojing Zou, Shiying Yuan, and You Shang (2020), Clinical course and outcomes of Critically Ill patients with SARS-CoV-2 pneumonia in Wuhan, China: a single-centered, retrospective, observational study. *Lancet Respiratory Medicine* 8: 475–481.

Table S-1. Comparison of COVID-19 deaths per text analysis, to ICD-10 codes

Table shows counts, for January 1, 2020, through December 31, 2022, of COVID-19 and other natural-cause deaths for persons aged 15+ determined from our text analysis to COVID-19 deaths, determined using ICD-10 codes from NCHS (the code for COVID-19 death is U07.1). ICD-10 codes are generated by NCHS, based on the text fields in death certificates. Deaths from natural causes are all deaths except those due to accident, homicide, or suicide.

**Natural cause deaths**	**COVID-19 by Text Analysis**	**non-COVID-19 by Text Analysis**	**Total**
COVID-19 by ICD-10 Code	21,294	676	**21,970**
non-COVID-19 by ICD-10 Code	771	189,929	**190,700**
**Total**	**22,065**	**190,605**	**212,670**

Table S-2. Vaccine Approval Dates

Table shows CDC approval dates for indicated vaccine types and number of doses. CDC approval is soon after FDA approval. Some approvals were for limited age groups or populations (e.g., high-risk workers).

Vaccine	Primary vaccination	1st booster dose	2nd booster dose
Pfizer	2020.12.14	2021.09.24	2022.04.30
Moderna	2020.12.18	2021.10.21	2022.04.30
J&J	2021.02.26		
Novavax	2022.07.13		

Table S-3. Summary Statistics on Vaccination Status

Table provides summary information on vaccine doses for persons aged 15+ vaccinated in Indiana as of December 31, 2021, and December 31, 2022. Vaccine type for fully vaccinated persons is based on first two doses or, if different, first dose. We report results for 1 or 2 doses based on number of doses, thus treating one J&J dose as equivalent to one mRNA dose; but treat J&J followed by an mRNA dose as 3 doses, thus treating the mRNA dose as a booster dose, and similarly treat J&J followed by two mRNA doses as 4 doses (thus, as receiving two booster doses). More formally, we define persons as receiving a booster dose if they: (1) Received primary 2-dose mRNA vaccination, and then received a third mRNA dose after booster doses for the mRNA vaccines were approved by FDA and CDC (Sept. 24, 2021, for Pfizer; Oct. 21, 2021, for Moderna); or (2) Received an initial J&J dose, and then received an mRNA dose after booster doses for J&J recipients were approved by FDA and CDC (Oct. 21, 2021). Excludes immune-compromised persons. Estimated Indiana population aged 15+ was 5,459,479 people.

		Dec. 31, 2021		Dec. 31, 2022	
**No. of Doses**	**Vaccine Type**	**People**	**% of pop.**	**People**	**% of pop.**
1 dose	J&J	196,307	3.60%	173,870	3.18%
	Moderna	117,001	2.14%	119,270	2.18%
	Pfizer	170,915	3.13%	179,149	3.28%
	Novavax	0	0.00%	59	0.00%
	All 1 dose	**484,223**	**8.87%**	**472,348**	**8.65%**
2 doses (excl. J&J plus mRNA booster)	Moderna Only	627,566	11.49%	523,860	9.60%
	Pfizer Only	1,117,719	20.47%	951,174	17.42%
	J&J Only	18,461	0.34%	18,586	0.34%
	Novavax only	0	0.00%	87	0.00%
	Mixed mRNA	5,046	0.09%	9,117	0.17%
	mRNA, then J&J	856	0.02%	849	0.02%
	Other patterns	1,401	0.03%	847	0.02%
	All primary vax	**1,771,049**	**32.44%**	**1,504,520**	**27.56%**
Booster dose	Moderna Only	480,591	8.80%	373,815	6.85%
	Pfizer Only	719,648	13.18%	637,943	11.69%
	Mixed mRNA	114,385	2.10%	126,277	2.31%
	J&J, then mRNA	54,951	1.01%	66,196	1.21%
	Mixed J&J and mRNA	0	0.00%	0	0.00%
	Other patterns	0	0.00%	0	0.00%
	All first booster	**1,369,575**	**25.09%**	**1,204,231**	**22.06%**
Second booster dose or more	Moderna Only	0	0.00%	182,931	3.35%
	Pfizer Only	0	0.00%	275,895	5.05%
	Mixed mRNA	0	0.00%	119,767	2.19%
	J&J and then 2 mRNA	0	0.00%	16,276	0.30%
	Other patterns	0	0.00%	46	0.00%
	All second booster		**0.00%**	**594,915**	**10.90%**
Other		45,192	0.83%	49,857	0.91%
**All vaccinated**		3,670,039	67.22%	3,825,871	70.08%
**Unvaccinated**		1,789,440	32.78%	1,633,608	29.92%
**Total population**		5,459,479	100.00%	5,459,479	100.00%

Table S-4. Healthy Vaccinee Bias Correction Factor (HVBCF)".

Sample is same as text Figure 2. Table shows non-COVID natural deaths, non-COVID Natural Mortality Rate (NCNMR) and healthy vaccinee bias correction factor (relative NCNMR for vaccinated (with indicated number of doses) versus unvaccinated), by calendar quarter over 1Q2021-4Q2022. **Panel A**. Broad age groups (ages 15-59 and 60+), underlying Figure 2. **Panel B**. Age groups 15-39 and 40-59. **Panel C**. Age groups; 60-79; 80+. **All panels**. NCNMR is number of Non-COVID-19 natural deaths among persons within indicated age group and vaccination status over indicated period, divided by estimated population in the same age group, vaccination status, and time period. Population with given number of vaccine doses is measured monthly and averaged over each quarter. Counts for 3Q-4Q 2021 are adjusted for IHIE undercount of vaccinated decedents during August-October 2021.

For each calendar quarter and age group, we assign the sample population to a vaccine dose category based on the number of people in each category (measured at the beginning of each month) averaged over the three months in the quarter. For each category in the table (by quarter, age group, and number of vaccine doses) we measure the number of non-COVID-19 natural deaths. We then measure HVBCF for each vaccinated group relative to the unvaccinated.

Panel A. Healthy Vaccinee Bias Correction Factor for Ages 15-59 and 60+

		Age 15-59					Age 60+				
**Period**	**Vaccine doses**	**Population at start of period**	**Population at end of period**	**Non-COVID nat. deaths**	**NCNMR**	**HVBCF vs. unvaxed**	**Population at start of period**	**Population at end of period**	**Non-COVID nat. deaths**	**NCNMR**	**HVBCF vs. unvaxed**
**1Q 2021**	0	3,827,287	3,174,369	2,049	0.06%	n.m.	1,519,603	468,891	11,229	1.13%	n.m.
	1	87,112	419,149	43	0.02%	0.290	25,477	265,928	1,374	0.94%	0.835
	2	0	320,881	14	0.01%	0.149	0	810,261	559	0.14%	0.122
	1 or 2	87,112	740,030	57	0.01%	0.236	25,477	1,076,189	1,933	0.35%	0.311
**2Q2021**	0	3,174,369	2,182,349	1,506	0.06%	n.m.	468,891	301,865	6,101	1.58%	n.m.
	1	419,149	300,651	182	0.05%	0.899	265,928	86,995	1,050	0.60%	0.376
	2	320,881	1,431,399	248	0.03%	0.503	810,261	1,156,220	5,409	0.55%	0.348
	1 or 2	740,030	1,732,050	430	0.03%	0.619	1,076,189	1,243,215	6,459	0.56%	0.352
**3Q2021**	0	2,182,349	1,814,061	1,401	0.07%	n.m.	301,865	234,663	5,413	2.02%	n.m.
	1	300,651	369,733	158	0.05%	0.672	86,995	102,185	673	0.71%	0.353
	2	1,431,399	1,708,130	531	0.03%	0.482	1,156,220	1,121,444	6,855	0.60%	0.298
	1 or 2	1,732,050	2,077,863	689	0.04%	0.516	1,243,215	1,223,629	7,529	0.61%	0.303
	3	0	12,328	0	0.00%	0.000	0	58,314	0	0.00%	0.000
**4Q2021**	0	1,814,061	1,616,807	1,314	0.08%	n.m.	234,663	172,633	5,247	2.58%	n.m.
	1	369,733	365,508	210	0.06%	0.744	102,185	118,715	773	0.70%	0.272
	2	1,708,130	1,305,880	679	0.05%	0.589	1,121,444	465,169	7,054	0.89%	0.345
	1 or 2	2,077,863	1,671,388	889	0.05%	0.619	1,223,629	583,884	7,826	0.87%	0.336
	3	12,328	613,971	19	0.01%	0.079	58,314	755,604	857	0.21%	0.082
**1Q2022**	0	1,616,807	1,526,851	1,117	0.07%	n.m.	172,633	159,946	4,762	2.86%	n.m.
	1	365,508	364,292	160	0.04%	0.617	118,715	115,813	668	0.57%	0.199
	2	1,305,880	1,169,924	550	0.04%	0.625	465,169	391,410	4,014	0.94%	0.327
	1 or 2	1,671,388	1,534,216	710	0.04%	0.623	583,884	507,223	4,682	0.86%	0.300

Period	Vaccine doses	Age 15-59 Population at start of period	Population at end of period	Non-COVID nat. deaths	NCNMR	HVBCF vs. unvaxed	Age 60+ Population at start of period	Population at end of period	Non-COVID nat. deaths	NCNMR	HVBCF vs. unvaxed
	3	613,971	839,397	227	0.03%	0.440	755,604	837,028	4,307	0.54%	0.189
**2Q2022**	0	1,526,851	1,508,934	1,058	0.07%	n.m.	159,946	154,055	4,008	2.55%	n.m.
	1	364,292	364,461	130	0.04%	0.512	115,813	110,962	559	0.49%	0.193
	2	1,169,924	1,159,356	421	0.04%	0.519	391,410	386,028	2,844	0.73%	0.287
	1 or 2	1,534,216	1,523,817	551	0.04%	0.517	507,223	496,990	3,403	0.68%	0.266
	3	839,397	833,455	246	0.03%	0.422	837,028	667,883	4,769	0.63%	0.248
	4	1,204	34,845	5	0.03%	0.398	7,777	192,514	197	0.20%	0.077
**3Q2022**	0	1,508,934	1,495,444	994	0.07%	n.m.	154,055	149,899	4,205	2.77%	n.m.
	1	364,461	365,921	119	0.03%	0.493	110,962	109,302	508	0.46%	0.167
	2	1,159,356	1,152,586	441	0.04%	0.577	386,028	380,127	2,631	0.69%	0.248
	1 or 2	1,523,817	1,518,507	560	0.04%	0.556	496,990	489,429	3,139	0.64%	0.230
	3	835,911	805,320	313	0.04%	0.576	669,471	584,757	4,196	0.67%	0.242
	4	32,389	81,162	28	0.05%	0.745	190,926	286,906	1,239	0.52%	0.187
**4Q2022**	0	1,495,444	1,487,532	1,144	0.08%	n.m.	149,899	146,076	4,549	3.07%	n.m.
	1	365,921	365,076	161	0.04%	0.574	109,302	107,272	601	0.56%	0.181
	2	1,152,586	1,137,972	479	0.04%	0.545	380,127	366,548	2,924	0.78%	0.255
	1 or 2	1,518,507	1,503,048	640	0.04%	0.552	489,429	473,820	3,525	0.73%	0.238
	3	805,320	725,227	314	0.04%	0.535	584,757	479,004	4,055	0.76%	0.248
	4	81,162	183,593	67	0.05%	0.660	286,906	411,322	2,123	0.61%	0.198

Panel B. Healthy Vaccinee Bias for Ages 15-39 and 40-59

Period	Vaccine doses	Age 15-39 Population at start of period	Population at end of period	Non-COVID nat. deaths	NCNMR	HVBCF vs. unvaxed	Age 40-59 Population at start of period	Population at end of period	Non-COVID nat. deaths	NCNMR	HVBCF vs. unvaxed
**1Q 2021**	0	2,212,177	2,033,303	281	0.01%	n.m..	1,615,110	1,141,066	1,768	0.13%	n.m.
	1	37,980	95,855	5	0.01%	0.564	49,132	323,294	38	0.02%	0.159
	2	0	120,999	0	0.00%	0.000	0	199,882	14	0.01%	0.109
	1 or 2	37,980	216,854	5	0.00%	0.296	49,132	523,176	52	0.02%	0.142
**2Q2021**	0	2,033,303	1,417,928	221	0.01%	n.m..	1,141,066	764,421	1,285	0.13%	n.m.
	1	95,855	153,696	14	0.01%	0.876	323,294	146,955	168	0.07%	0.530
	2	120,999	678,533	17	0.00%	0.332	199,882	752,866	231	0.05%	0.360
	1 or 2	216,854	832,229	31	0.01%	0.462	523,176	899,821	399	0.06%	0.416

Period	Vaccine doses	Age 15-39					Age 40-59				
		Population at start of period	Population at end of period	Non-COVID nat. deaths	NCNMR	HVBCF vs. unvaxed	Population at start of period	Population at end of period	Non-COVID nat. deaths	NCNMR	HVBCF vs. unvaxed
**3Q2021**	0	1,417,928	1,193,321	217	0.02%	n.m..	764,421	620,740	1,185	0.17%	n.m.
	1	153,696	191,058	14	0.01%	0.471	146,955	178,675	144	0.09%	0.519
	2	678,533	858,968	46	0.01%	0.357	752,866	849,162	485	0.06%	0.354
	1 or 2	832,229	1,050,026	59	0.01%	0.377	899,821	1,027,837	630	0.07%	0.382
	3	0	4,039	0	0.00%	0.000	0	8,289	0	0.00%	0.000
**4Q2021**	0	1,193,321	1,074,165	213	0.02%	n.m..	620,740	542,642	1,103	0.19%	n.m.
	1	191,058	202,968	24	0.01%	0.634	178,675	162,540	186	0.11%	0.575
	2	858,968	731,093	63	0.01%	0.421	849,162	574,787	616	0.09%	0.456
	1 or 2	1,050,026	934,061	87	0.01%	0.463	1,027,837	737,327	802	0.09%	0.479
	3	4,039	238,549	4	0.00%	0.175	8,289	375,422	15	0.01%	0.041
**1Q2022**	0	1,074,165	1,012,360	192	0.02%	n.m..	542,642	514,491	925	0.18%	n.m.
	1	202,968	208,136	22	0.01%	0.582	162,540	156,156	138	0.09%	0.495
	2	731,093	668,204	79	0.01%	0.614	574,787	501,720	471	0.09%	0.500
	1 or 2	934,061	876,340	101	0.01%	0.606	737,327	657,876	609	0.09%	0.499
	3	238,549	357,808	16	0.01%	0.292	375,422	481,589	211	0.05%	0.281
**2Q2022**	0	1,012,360	999,307	162	0.02%	n.m..	514,491	509,627	896	0.17%	n.m.
	1	208,136	209,162	17	0.01%	0.506	156,156	155,299	113	0.07%	0.415
	2	668,204	664,555	40	0.01%	0.373	501,720	494,801	381	0.08%	0.437
	1 or 2	876,340	873,717	57	0.01%	0.404	657,876	650,100	494	0.08%	0.432
	3	357,808	371,041	30	0.01%	0.511	481,589	462,414	216	0.05%	0.262
	4	96	2,432	0	0.00%	0.000	1,108	32,413	5	0.03%	0.171
**3Q2022**	0	999,307	989,046	159	0.02%	n.m..	509,627	506,398	835	0.16%	n.m.
	1	209,162	210,877	16	0.01%	0.476	155,299	155,044	103	0.07%	0.404
	2	664,555	662,414	41	0.01%	0.386	494,801	490,172	400	0.08%	0.494
	1 or 2	873,717	873,291	57	0.01%	0.408	650,100	645,216	503	0.08%	0.473
	3	371,041	367,876	11	0.00%	0.186	464,870	437,444	302	0.07%	0.407
	4	2,432	16,152	1	0.01%	0.673	29,957	65,010	27	0.06%	0.346
**4Q2022**	0	989,046	983,281	172	0.02%	n.m..	506,398	504,251	972	0.19%	n.m.
	1	210,877	210,975	19	0.01%	0.517	155,044	154,101	142	0.09%	0.478
	2	662,414	656,056	51	0.01%	0.444	490,172	481,916	428	0.09%	0.458
	1 or 2	873,291	867,031	70	0.01%	0.461	645,216	636,017	570	0.09%	0.463
	3	367,876	337,237	24	0.01%	0.390	437,444	387,990	290	0.07%	0.365
	4	16,152	58,555	6	0.02%	0.921	65,010	125,038	61	0.06%	0.334

Panel C. Healthy Vaccinee Bias for Ages 60-79 and 80+

		Age 60-79					Age 80+				
Period	Vaccine doses	Population at start of period	Population at end of period	Non-COVID nat. deaths	NCNMR	HVBCF vs. unvaxed	Population at start of period	Population at end of period	Non-COVID nat. deaths	NCNMR	HVBCF vs. unvaxed
**1Q 2021**	0	1,262,449	420,757	6,066	0.72%	n.m..	257,154	48,134	5,163	3.38%	n.m.
	1	22,958	244,311	496	0.37%	0.515	2,519	21,617	878	7.28%	2.151
	2	0	620,339	170	0.05%	0.076	**0**	189,922	389	0.41%	0.121
	1 or 2	22,958	864,650	666	0.15%	0.208	2,519	211,539	1,267	1.18%	0.350
**2Q2021**	0	420,757	271,068	3,592	1.04%	n.m.	48,134	30,797	2,509	6.36%	n.m.
	1	244,311	74,634	650	0.41%	0.393	21,617	12,361	400	2.35%	0.370
	2	620,339	939,705	2,332	0.30%	0.288	189,922	216,515	3,077	1.51%	0.238
	1 or 2	864,650	1,014,339	2,982	0.32%	0.306	211,539	228,876	3,477	1.58%	0.248
**3Q2021**	0	271,068	210,860	3,151	1.31%	n.m.	30,797	23,803	2,265	8.30%	n.m.
	1	74,634	88,118	346	0.43%	0.325	12,361	14,067	329	2.49%	0.300
	2	939,705	917,708	3,100	0.33%	0.255	216,515	203,736	3,755	1.79%	0.215
	1 or 2	1,014,339	1,005,826	3,446	0.34%	0.261	228,876	217,803	4,083	1.83%	0.220
	3	0	46,042	0	0.00%	0.000	0	12,272	0	0.00%	0.000
**4Q2021**	0	210,860	159,752	3,039	1.64%	n.m.	23,803	12,881	2,234	12.18%	n.m.
	1	88,118	98,856	441	0.47%	0.288	14,067	19,859	333	1.96%	0.161
	2	917,708	387,301	3,257	0.50%	0.304	203,736	77,868	3,797	2.70%	0.221
	1 or 2	1,005,826	486,157	3,696	0.50%	0.302	217,803	97,727	4,130	2.62%	0.215
	3	46,042	613,431	339	0.10%	0.063	12,272	142,173	518	0.67%	0.055
**1Q2022**	0	159,752	148,690	2,726	1.77%	n.m.	12,881	11,256	2,036	16.87%	n.m.
	1	98,856	95,946	375	0.39%	0.218	19,859	19,867	293	1.48%	0.087
	2	387,301	323,684	2,069	0.58%	0.329	77,868	67,726	1,945	2.67%	0.158
	1 or 2	486,157	419,630	2,444	0.54%	0.305	97,727	87,593	2,238	2.42%	0.143
	3	613,431	684,277	1,830	0.28%	0.160	142,173	152,751	2,477	1.68%	0.100
**2Q2022**	0	148,690	144,227	2,351	1.61%	n.m.	11,256	9,828	1,657	15.72%	n.m.
	1	95,946	91,994	331	0.35%	0.219	19,867	18,968	228	1.17%	0.075
	2	323,684	318,967	1,474	0.46%	0.286	67,726	67,061	1,370	2.03%	0.129
	1 or 2	419,630	410,961	1,805	0.43%	0.271	87,593	86,029	1,598	1.84%	0.117
	3	684,277	551,656	2,166	0.35%	0.218	152,751	116,227	2,603	1.94%	0.123
	4	6,606	151,906	96	0.12%	0.076	1,171	40,608	101	0.48%	0.031
**3Q2022**	0	144,227	141,056	2,458	1.72%	n.m.	9,828	8,843	1,747	18.71%	n.m.
	1	91,994	90,557	287	0.31%	0.183	18,968	18,745	221	1.17%	0.063
	2	318,967	314,038	1,395	0.44%	0.256	67,061	66,089	1,236	1.86%	0.099
	1 or 2	410,961	404,595	1,682	0.41%	0.239	86,029	84,834	1,457	1.71%	0.091

		Age 60-79					Age 80+				
**Period**	**Vaccine doses**	**Population at start of period**	**Population at end of period**	**Non-COVID nat. deaths**	**NCNMR**	**HVBCF vs. unvaxed**	**Population at start of period**	**Population at end of period**	**Non-COVID nat. deaths**	**NCNMR**	**HVBCF vs. unvaxed**
	3	553,087	484,382	1,953	0.38%	0.219	116,384	100,375	2,243	2.07%	0.111
	4	150,475	228,321	442	0.23%	0.135	40,451	58,585	797	1.61%	0.086
**4Q2022**	0	141,056	138,154	2,646	1.90%	n.m.	8,843	7,922	1,903	22.70%	n.m.
	1	90,557	88,928	359	0.40%	0.211	18,745	18,344	242	1.30%	0.058
	2	314,038	303,092	1,572	0.51%	0.269	66,089	63,456	1,352	2.09%	0.092
	1 or 2	404,595	392,020	1,931	0.48%	0.256	84,834	81,800	1,594	1.91%	0.084
	3	484,382	397,663	1,919	0.44%	0.230	100,375	81,341	2,136	2.35%	0.104
	4	228,321	329,832	770	0.28%	0.146	58,585	81,490	1,353	1.93%	0.085

Table S-5. Relative Mortality Risk (Based on *CEMP*)

Sample is same as text Figure 2 and Table S-4. Table shows COVID-19 deaths, non-COVID natural deaths, *CEMP* (COVID Excess Mortality Percentage), and *RMR* (Relative Mortality Risk) for vaccinated (with indicated number of doses) versus unvaccinated, by calendar quarter over 1Q2021-4Q2022. **Panel A**. Ages 15-59 and 60+. **Panel B**. Ages 15-39 and 40-59. **Panel C**. Ages 60-79; 80+). **All panels**. Population with given number of vaccine doses is measured monthly and averaged over each quarter. Counts for 3Q-4Q 2021 are adjusted for IHIE undercount of vaccinated decedents during August-October 2021.

Panel A. Relative Mortality Risk (*RMR*) for Broad Age Groups: Ages 15-59 and 60+

	Age 15-59						Age 60+				
**Period**	**Vaccine doses**	**COVID deaths**	**Non-COVID nat. deaths**	***CEMP***	***RMR* vs. unvaxed**	**95% CI (%)**	**COVID deaths**	**Non-COVID nat. deaths**	***CEMP***	***RMR* vs. unvaxed**	**95% CI**
**1Q 2021**	0	233	2049	11.37%	n.a.	n.a.	2348	11229	20.91%	n.a.	n.a.
	1	3	43	6.98%	0.614	[0.189, 1.994]	65	1374	4.73%	0.226	[0.176, 0.291]
	2	0	14	0.00%	0.000		11	559	1.97%	0.094	[0.052, 0.171]
	1 or 2	3	57	5.26%	0.463	[0.144, 1.490]	76	1933	3.93%	0.188	[0.149, 0.238]
**2Q2021**	0	120	1,506	7.97%	n.a.	n.a.	380	6,101	6.23%	n.a.	n.a.
	1	6	182	3.30%	0.414	[0.180, 0.953]	49	1050	4.67%	0.749	[0.553, 1.016]
	2	7	248	2.82%	0.354	[0.163, 0.768]	59	5409	1.09%	0.175	[0.133, 0.231]
	1 or 2	13	430	3.02%	0.379	[0.212, 0.679]	108	6,459	1.67%	0.269	[0.216, 0.333]
**3Q2021 (adjusted)**	0	437.72	1401.03	31.24%	n.a.	n.a.	1020.99	5412.76	18.86%	n.a.	n.a.
	1	13.85	157.90	8.77%	0.281	[0.146, 0.541]	48.82	672.68	7.26%	0.385	[0.273, 0.543]
	2	22.75	530.75	4.29%	0.137	[0.085, 0.222]	280.60	6854.90	4.09%	0.217	[0.187, 0.252]
	1 or 2	36.13	689.12	5.24%	0.168	[0.113, 0.249]	328.39	7528.61	4.36%	0.231	[0.201, 0.266]
**4Q2021 (adjusted)**	0	673.86	1313.89	51.29%	n.a.	n.a.	1818.75	5247.00	34.66%	n.a.	n.a.
	1	28.15	209.60	13.43%	0.262	[0.173, 0.396]	109.16	773.34	14.12%	0.407	[0.330, 0.502]
	2	39.00	679.50	5.74%	0.112	[0.080, 0.157]	655.66	7053.84	9.30%	0.268	[0.243, 0.296]
	1 or 2	66.98	889.27	7.53%	0.147	[0.112, 0.192]	765.88	7826.12	9.79%	0.282	[0.257, 0.310]
	3	0	19	0.00%	0.000		30	857	3.50%	0.108	[0.075, 0.156]
**1Q2022**	0	435	1,117	38.94%	n.a.	n.a.	1605	4,762	33.70%	n.a.	n.a.
	1	25	160	15.63%	0.401	[0.260, 0.620]	129	668	19.31%	0.573	[0.471, 0.698]
	2	69	550	12.55%	0.322	[0.245, 0.424]	623	4014	15.52%	0.461	[0.416, 0.510]
	1 or 2	94	710	13.24%	0.340	[0.267, 0.433]	752	4,682	16.06%	0.477	[0.433, 0.524]
	3	11	227	4.85%	0.124	[0.067, 0.230]	221	4,307	5.13%	0.152	[0.132, 0.176]
**2Q2022**	0	19	1058	1.80%	n.a.	n.a.	85	4008	2.12%	n.a.	n.a.
	1	0	130	0.00%	0.000		16	559	2.86%	1.350	[0.785, 2.319]
	2	13	421	3.09%	1.720	[0.841, 3.514]	71	2844	2.50%	1.177	[0.856, 1.619]
	1 or 2	13	551	2.36%	1.314	[0.644, 2.681]	87	3403	2.56%	1.206	[0.891, 1.631]
	3	3	246	1.22%	0.679	[0.199, 2.314]	88	4769	1.85%	0.870	[0.644, 1.176]

		Age 15-59					Age 60+				
**Period**	**Vaccine doses**	**COVID deaths**	**Non-COVID nat. deaths**	***CEMP***	***RMR* vs. unvaxed**	**95% CI (%)**	**COVID deaths**	**Non-COVID nat. deaths**	***CEMP***	***RMR* vs. unvaxed**	**95% CI**
	4	0	5	0.00%	0.000		2	197	1.02%	0.479	[0.117, 1.960]
**3Q2022**	0	32	994	3.22%	n.a.	n.a.	169	4205	4.02%	n.a.	n.a.
	1	5	119	4.20%	1.305	[0.499, 3.415]	17	508	3.35%	0.833	[0.501, 1.383]
	2	11	441	2.49%	0.775	[0.387, 1.551]	107	2631	4.07%	1.012	[0.790, 1.295]
	1 or 2	16	560	2.86%	0.888	[0.483, 1.632]	124	3139	3.95%	0.983	[0.776, 1.245]
	3	4	313	1.28%	0.397	[0.139, 1.132]	147	4196	3.50%	0.872	[0.696, 1.092]
	4	1	28	3.57%	1.109	[0.146, 8.413]	35	1239	2.82%	0.703	[0.486, 1.017]
**4Q2022**	0	29	1144	2.53%	n.a.	n.a.	184	4549	4.04%	n.a.	n.a.
	1	2	161	1.24%	0.490	[0.116, 2.074]	16	601	2.66%	0.658	[0.392, 1.105]
	2	12	479	2.51%	0.988	[0.500, 1.953]	110	2924	3.76%	0.930	[0.731, 1.183]
	1 or 2	14	640	2.19%	0.863	[0.453, 1.645]	126	3525	3.57%	0.884	[0.702, 1.113]
	3	3	314	0.96%	0.377	[0.114, 1.246]	143	4055	3.53%	0.872	[0.698, 1.089]
	4	1	67	1.49%	0.589	[0.079, 4.390]	79	2123	3.72%	0.920	[0.703, 1.204]

Panel B. Relative Mortality Risk (*RMR*) for Ages 15-39 and 40-59

		Age 15-39					Age 40-59				
**Period**	**Vaccine doses**	**COVID deaths**	**Non-COVID nat. deaths**	***CEMP***	***RMR* vs. unvaxed**	**95% CI**	**COVID deaths**	**Non-COVID nat. deaths**	***CEMP***	***RMR* vs. unvaxed**	**95% CI**
**1Q 2021**	0	17	281	6.05%	n.a.	n.a.	216	1768	12.22%	n.a.	n.a.
	1	0	5	0.00%	0.000		3	38	7.89%	0.646	[0.198, 2.112]
	2	0	0				0	14	0.00%	0.000	
	1 or 2	0	5	0.00%	0.000		3	52	5.77%	0.472	[0.146, 1.526]
**2Q2021**	0	14	221	6.33%	n.a.	n.a.	106	1285	8.25%	n.a.	n.a.
	1	0	14	0.00%	0.000		6	168	3.57%	0.433	[0.187, 1.001]
	2	1	17	5.88%	0.929	[0.115, 7.522]	6	231	2.60%	0.315	[0.137, 0.725]
	1 or 2	1	31	3.23%	0.509	[0.064, 4.024]	12	399	3.01%	0.365	[0.199, 0.669]
**3Q2021**	0	9.0	217.0	27.18%	n.a.	n.a.	632.3	1184.8	53.37%	n.a.	n.a.
**(adjusted)**	1	0.0	13.5	0.00%			13.8	144.5	9.52%	0.304	[0.157, 0.587]
	2	2.4	45.6	5.26%	0.172	[0.040, 0.732]	20.3	485.2	4.19%	0.134	[0.080, 0.223]
	1 or 2	2.5	59.0	4.26%	0.139	[0.033, 0.588]	33.6	630.1	5.34%	0.170	[0.113, 0.257]
**4Q2021**	0	90.5	213.5	42.40%	n.a.	n.a.	819.8	1103.2	74.30%	n.a.	n.a.
**(adjusted)**	1	0.0	23.5	0.00%			28.1	186.2	15.08%	0.291	[0.191, 0.441]
	2	1.0	63.0	1.52%	0.032	[0.004, 0.235]	38.3	616.2	6.21%	0.120	[0.085, 0.169]
	1 or 2	1.0	86.5	1.14%	0.024	[0.003, 0.175]	66.3	802.5	8.26%	0.159	[0.121, 0.209]
	3	0	4	0.00%	0.000		0	15	0.00%	0.000	

		Age 15-39					Age 40-59				
**Period**	**Vaccine doses**	**COVID deaths**	**Non-COVID nat. deaths**	***CEMP***	***RMR* vs. unvaxed**	**95% CI**	**COVID deaths**	**Non-COVID nat. deaths**	***CEMP***	***RMR* vs. unvaxed**	**95% CI**
**1Q2022**	0	61	192	31.77%	n.a.	n.a.	374	925	40.43%	n.a.	n.a.
	1	3	22	13.64%	0.429	[0.124, 1.486]	22	138	15.94%	0.394	[0.248, 0.628]
	2	8	79	10.13%	0.319	[0.146, 0.698]	61	471	12.95%	0.320	[0.239, 0.429]
	1 or 2	11	101	10.89%	0.343	[0.173, 0.681]	83	609	13.63%	0.337	[0.260, 0.437]
	3	0	16	0.00%	0.000		11	211	5.21%	0.129	[0.070, 0.239]
**2Q2022**	0	4	162	2.47%	n.a.	n.a.	15	896	1.67%	n.a.	n.a.
	1	0	17	0.00%	0.000		0	113	0.00%	0.000	
	2	1	40	2.50%	1.013	[0.110, 9.358]	12	381	3.15%	1.881	[0.872, 4.058]
	1 or 2	1	57	1.75%	0.711	[0.077, 6.522]	12	494	2.43%	1.451	[0.674, 3.125]
	3	0	30	0.00%	0.000		3	216	1.39%	0.830	[0.238, 2.892]
	4	0	0				0	5	0.00%	0.000	
**3Q2022**	0	7	159	4.40%	n.a.	n.a.	25	835	2.99%	n.a.	n.a.
	1	1	16	6.25%	1.420	[0.163, 12.338]	4	103	3.88%	1.297	[0.443, 3.802]
	2	1	41	2.44%	0.554	[0.066, 4.652]	10	400	2.50%	0.835	[0.397, 1.756]
	1 or 2	2	57	3.51%	0.797	[0.160, 3.963]	14	503	2.78%	0.930	[0.479, 1.805]
	3	0	11	0.00%	0.000		4	302	1.32%	0.442	[0.153, 1.282]
	4	0	1	0.00%	0.000		1	27	3.70%	1.237	[0.162, 9.474]
**4Q2022**	0	5	172	2.91%	n.a.	n.a.	24	972	2.47%	n.a.	n.a.
	1	0	19	0.00%	0.000		2	142	1.41%	0.570	[0.133, 2.441]
	2	1	51	1.96%	0.675	[0.077, 5.934]	11	428	2.57%	1.041	[0.505, 2.145]
	1 or 2	1	70	1.43%	0.491	[0.056, 4.301]	13	570	2.28%	0.924	[0.467, 1.829]
	3	0	24	0.00%	0.000		3	290	1.03%	0.419	[0.125, 1.403]
	4	0	6	0.00%	0.000		1	61	1.64%	0.664	[0.088, 4.993]

Panel C. Relative Mortality Risk (*RMR*) for Ages 60-79 and 80+

		Age 60-79					Age 80+				
**Period**	**Vaccine doses**	**COVID deaths**	**Non-COVID nat. deaths**	***CEMP***	***RMR* vs. unvaxed**	**95% CI**	**COVID deaths**	**Non-COVID nat. deaths**	***CEMP***	***RMR* vs. unvaxed**	**95% CI**
**1Q 2021**	0	1190	6066	19.62%	n.a.	n.a.	1158	5163	22.43%	n.a.	n.a.
	1	21	496	4.23%	0.216	[0.139, 0.336]	44	878	5.01%	0.223	[0.164, 0.305]
	2	3	170	1.76%	0.090	[0.029, 0.282]	8	389	2.06%	0.092	[0.045, 0.185]
	1 or 2	24	666	3.60%	0.184	[0.122, 0.277]	52	1267	4.10%	0.183	[0.138, 0.243]
**2Q2021**	0	268	3592	7.46%	n.a.	n.a.	112	2509	4.46%	n.a.	n.a.
	1	34	650	5.23%	0.701	[0.486, 1.011]	15	400	3.75%	0.840	[0.485, 1.455]
	2	25	2332	1.07%	0.144	[0.095, 0.217]	34	3077	1.10%	0.248	[0.168, 0.365]

		Age 60-79					Age 80+				
**Period**	**Vaccine doses**	**COVID deaths**	**Non-COVID nat. deaths**	***CEMP***	***RMR* vs. unvaxed**	**95% CI**	**COVID deaths**	**Non-COVID nat. deaths**	***CEMP***	***RMR* vs. unvaxed**	**95% CI**
	1 or 2	59	2982	1.98%	0.265	[0.199, 0.353]	49	3477	1.41%	0.316	[0.225, 0.443]
**3Q2021 (adjusted)**	0	3501.78	3151.22	111.12%	n.a.	n.a.	4086.84	3039.16	134.47%	n.a.	n.a.
	1	35.09	345.91	10.14%	0.443	[0.298, 0.658]	66.13	440.87	15.00%	0.361	[0.278, 0.469]
	2	120.25	3099.50	3.88%	0.169	[0.136, 0.210]	289.04	3256.71	8.88%	0.214	[0.186, 0.245]
	1 or 2	154.95	3445.80	4.50%	0.196	[0.161, 0.238]	357.08	3695.67	9.66%	0.233	[0.205, 0.264]
**4Q2021 (adjusted)**	0	4086.84	3039.16	134.47%	n.a.	n.a.	4713.89	2234.11	211.00%	n.a.	n.a.
	1	66.13	440.87	15.00%	0.361	[0.278, 0.469]	42.47	333.03	12.75%	0.510	[0.360, 0.723]
	2	289.04	3256.71	8.88%	0.214	[0.186, 0.245]	366.89	3796.86	9.66%	0.386	[0.335, 0.446]
	1 or 2	357.08	3695.67	9.66%	0.233	[0.205, 0.264]	408.96	4130.29	9.90%	0.396	[0.345, 0.455]
	3	15	339	4.42%	0.111	[0.066, 0.187]	15	518	2.90%	0.126	[0.075, 0.212]
**1Q2022**	0	1053	2726	38.63%	n.a.	n.a.	552	2036	27.11%	n.a.	n.a.
	1	68	375	18.13%	0.469	[0.359, 0.614]	61	293	20.82%	0.768	[0.574, 1.028]
	2	320	2069	15.47%	0.400	[0.349, 0.459]	303	1945	15.58%	0.575	[0.493, 0.670]
	1 or 2	388	2444	15.88%	0.411	[0.361, 0.467]	364	2238	16.26%	0.600	[0.519, 0.694]
	3	93	1830	5.08%	0.132	[0.106, 0.164]	128	2477	5.17%	0.191	[0.156, 0.233]
**2Q2022**	0	44	2351	1.87%	n.a.	n.a.	41	1657	2.47%	n.a.	n.a.
	1	8	331	2.42%	1.291	[0.603, 2.767]	8	228	3.51%	1.418	[0.657, 3.063]
	2	34	1474	2.31%	1.233	[0.784, 1.937]	37	1370	2.70%	1.092	[0.696, 1.712]
	1 or 2	42	1805	2.33%	1.243	[0.811, 1.906]	45	1598	2.82%	1.138	[0.741, 1.747]
	3	32	2166	1.48%	0.789	[0.499, 1.249]	56	2603	2.15%	0.870	[0.578, 1.307]
	4	0	96	0.00%	0.000		2	101	1.98%	0.800	[0.191, 3.356]
**3Q2022**	0	72	2458	2.93%	n.a.	n.a.	97	1747	5.55%	n.a.	n.a.
	1	7	287	2.44%	0.833	[0.380, 1.827]	10	221	4.52%	0.815	[0.419, 1.586]
	2	37	1395	2.65%	0.906	[0.606, 1.353]	70	1236	5.66%	1.020	[0.744, 1.399]
	1 or 2	44	1682	2.62%	0.893	[0.611, 1.306]	80	1457	5.49%	0.989	[0.730, 1.340]
	3	67	1953	3.43%	1.171	[0.835, 1.642]	80	2243	3.57%	0.642	[0.475, 0.869]
	4	10	442	2.26%	0.772	[0.396, 1.508]	25	797	3.14%	0.565	[0.361, 0.884]
**4Q2022**	0	91	2646	3.44%	n.a.	n.a.	93	1903	4.89%	n.a.	n.a.
	1	7	359	1.95%	0.567	[0.261, 1.233]	9	242	3.72%	0.761	[0.379, 1.528]
	2	44	1572	2.80%	0.814	[0.565, 1.173]	66	1352	4.88%	0.999	[0.723, 1.380]
	1 or 2	51	1931	2.64%	0.768	[0.542, 1.087]	75	1594	4.71%	0.963	[0.705, 1.315]
	3	56	1919	2.92%	0.849	[0.605, 1.190]	87	2136	4.07%	0.833	[0.618, 1.124]
	4	17	770	2.21%	0.642	[0.380, 1.084]	62	1353	4.58%	0.938	[0.675, 1.303]

Table S-6. *RMR*^{raw} Estimates

Sample is same as text Figure 2 and Table S-5. Table shows COVID-19 mortality rate (COVID-MR), and *RMR*^{raw} = CovidMR for people vaccinated with indicated number of doses/(COVID-MR for unvaccinated). CovidMR = COVID-19 deaths occurring among persons within indicated age groups with indicated vaccination status over indicated periods, divided by the estimated population of people in the same age group, vaccination status, and time period. Sample is same as text Figure 2 and Table S-4. Population with given number of vaccine doses is measured monthly and averaged over each quarter. Values for 3Q-4Q 2021 are adjusted for IHIE undercount of vaccinated decedents during August-October 2021.

		**Ages 15-59**		**Ages 60+**	
**Period**	**Vaccine doses**	**COVID-MR**	***RMR*^{raw}**	**COVID- MR**	***RMR*^{raw}**
**1Q 2021**	0	0.007%	--	0.236%	--
	1	0.001%	0.178	0.045%	0.189
	2	0.000%	0.000	0.003%	0.011
	1 or 2	0.001%	0.109	0.014%	0.058
**2Q2021**	0	0.004%	--	0.099%	--
	1	0.002%	0.372	0.028%	0.282
	2	0.001%	0.178	0.006%	0.061
	1 or 2	0.001%	0.235	0.009%	0.094
**3Q2021**	0	0.022%	--	0.381%	--
	1	0.004%	0.189	0.052%	0.136
	2	0.001%	0.066	0.025%	0.065
	1 or 2	0.002%	0.087	0.027%	0.070
	3	0.000%		0.000%	
**4Q2021**	0	0.039%	--	0.893%	--
	1	0.008%	0.195	0.099%	0.111
	2	0.003%	0.066	0.083%	0.093
	1 or 2	0.004%	0.091	0.085%	0.095
	3	0.000%		0.007%	0.008
**1Q2022**	0	0.028%	--	0.965%	--
	1	0.007%	0.248	0.110%	0.114
	2	0.006%	0.201	0.145%	0.151
	1 or 2	0.006%	0.212	0.138%	0.143
	3	0.002%		0.028%	0.029
**2Q2022**	0	0.002%	--	0.054%	--
	1	0.000%	0.892	0.014%	0.261
	2	0.001%	0.679	0.018%	0.337
	1 or 2	0.001%	0.287	0.017%	0.320
	3	0.000%		0.012%	0.216
	4	0.000%	--	0.002%	0.037
**3Q2022**	0	0.002%	0.643	0.111%	--
	1	0.001%	0.447	0.015%	0.139
	2	0.001%	0.494	0.028%	0.251
	1 or 2	0.001%	0.229	0.025%	0.226
	3	0.000%	0.827	0.023%	0.211
	4	0.002%	--	0.015%	0.132
**4Q2022**	0	0.002%	0.281	0.124%	--
	1	0.001%	--	0.015%	0.119
	2	0.001%	0.178	0.029%	0.237
	1 or 2	0.001%	0.000	0.026%	0.210
	3	0.000%	0.109	0.027%	0.216
	4	0.001%	--	0.023%	0.182

Table S-7. Multivariate Logit Results for *RMR*

Sample is same as text Figure 2 and Table S-4. Table compares *RMR* (based on *CEMP*) from Table S-5 to *RMR* estimates from multivariate regression, for vaccinated (with indicated number of doses) versus unvaccinated persons, by calendar quarter over 1Q2021-4Q2022 for ages 15-59 and 60+. 95% CI is for multivariate estimate. Values for 3Q-4Q 2021 are adjusted for IHIE undercount of vaccinated decedents during August-October 2021.

				**Ages 15-59**				**Ages 60+**	
**Period**	**Vaccine doses**	***CEMP***	***RMR* vs. unvaxed**	***RMR* (multivariate)**	**95% CI (%)**	***CEMP***	***RMR* vs. unvaxed**	***RMR* (multivariate)**	**95% CI**
**1Q 2021**	0	0.1137				0.2091			
	1	0.0698	0.6135	0.7600	[0.2308, 2.5027]	0.0473	0.2262***	0.2195***	[0.1702, 0.2831]
	2	0.0000				0.0197	0.0941***	0.0919***	[0.0504, 0.1675]
	1 or 2	0.0526	0.4628	0.5488	[0.1675, 1.7982]	0.0393	0.1880***	0.1828***	[0.1445, 0.2314]
**2Q2021**	0	0.0797				0.0623			
	1	0.0330	0.4137**	0.3900*	[0.1691, 0.8991]	0.0467	0.7492*	0.7413	[0.5454, 1.0074]
	2	0.0282	0.3542***	0.3168**	[0.1414, 0.7096]	0.0109	0.1751***	0.1955***	[0.1479, 0.2586]
	1 or 2	0.0302	0.3794***	0.3471***	[0.1901, 0.6338]	0.0167	0.2685***	0.2969***	[0.2382, 0.3702]
**3Q2021**	0	0.2861				0.1523			
	1	0.0877	0.2808***	0.2544***	[0.1300, 0.4976]	0.0726	0.3848***	0.3842***	[0.2725, 0.5418]
	2	0.0429	0.1372***	0.1272***	[0.0775, 0.2088]	0.0409	0.2170***	0.2239***	[0.1927, 0.2601]
	1 or 2	0.0524	0.1678***	0.1547***	[0.1030, 0.2323]	0.0436	0.2312***	0.2382***	[0.2068, 0.2744]
	3								
**4Q2021**	0	0.5034				0.3241			
	1	0.1343	0.2619***	0.2524***	[0.1653, 0.3854]	0.1412	0.4072***	0.4036***	[0.3269, 0.4982]
	2	0.0574	0.1119***	0.1091***	[0.0772, 0.1541]	0.0930	0.2682***	0.2822***	[0.2559, 0.3112]
	1 or 2	0.0753	0.1469***	0.1427***	[0.1084, 0.1877]	0.0979	0.2823***	0.2958***	[0.2696, 0.3245]
	3	0.0000				0.0350	0.1125***	0.1085***	[0.0750, 0.1571]
**1Q2022**	0	0.3894				0.3370			
	1	0.1563	0.4012***	0.3994***	[0.2567, 0.6216]	0.1931	0.5730***	0.5758***	[0.4723, 0.7020]
	2	0.1255	0.3221***	0.3027***	[0.2275, 0.4028]	0.1552	0.4605***	0.4658***	[0.4205, 0.5161]
	1 or 2	0.1324	0.3400***	0.3256***	[0.2531, 0.4188]	0.1606	0.4765***	0.4827***	[0.4383, 0.5315]
	3	0.0485	0.1244***	0.1119***	[0.0603, 0.2077]	0.0513	0.1522***	0.1557***	[0.1343, 0.1804]
**2Q2022**	0	0.0180				0.0212			
	1	0.0000		1.0000		0.0286	1.3496	1.3572	[0.7834, 2.3515]
	2	0.0309	1.7195	2.0699	[0.9461, 4.5287]	0.0250	1.1772	1.1310	[0.8133, 1.5726]
	1 or 2	0.0236	1.3138	1.6062	[0.7237, 3.5647]	0.0256	1.2055	1.1401	[0.8314, 1.5635]
	3	0.0122	0.6791	0.6528	[0.1821, 2.3403]	0.0185	0.8701	0.7974	[0.5800, 1.0963]
	4	0.0000				0.0102	0.4787	0.4144	[0.1003, 1.7119]
**3Q2022**	0	0.0322				0.0402			
	1	0.0420	1.3051	1.4007	[0.5215, 3.7624]	0.0335	0.8327	0.7844	[0.4702, 1.3084]
	2	0.0249	0.7748	0.8066	[0.4011, 1.6223]	0.0407	1.0119	0.9509	[0.7394, 1.2229]
	1 or 2	0.0286	0.8875	0.9041	[0.4885, 1.6734]	0.0395	0.9829	0.9139	[0.7165, 1.1656]
	3	0.0128	0.3970*	0.4109	[0.1328, 1.2710]	0.0350	0.8717	0.7860*	[0.6235, 0.9909]
	4	0.0357	1.1094	1.2246	[0.1369, 10.9526]	0.0282	0.7029*	0.5964**	[0.4098, 0.8679]

				Ages 15-59				Ages 60+	
**Period**	**Vaccine doses**	***CEMP***	***RMR* vs. unvaxed**	***RMR* (multivariate)**	**95% CI (%)**	***CEMP***	***RMR* vs. unvaxed**	***RMR* (multivariate)**	**95% CI**
**4Q2022**	0	0.0253				0.0404			
	1	0.0124	0.4900	0.5096	[0.1172, 2.2150]	0.0266	0.6582	0.6537	[0.3899, 1.0958]
	2	0.0251	0.9883	0.9223	[0.4598, 1.8503]	0.0376	0.9301	0.9135	[0.7173, 1.1633]
	1 or 2	0.0219	0.8629	0.8185	[0.4183, 1.6017]	0.0357	0.8837	0.8660	[0.6865, 1.0924]
	3	0.0096	0.3769	0.4109	[0.1183, 1.4275]	0.0353	0.8719	0.8166	[0.6519, 1.0228]
	4	0.0149	0.5888	0.5985	[0.0756, 4.7350]	0.0372	0.9200	0.8074	[0.6164, 1.0577]

Table S-8. Separate *RMR* Results for Men and Women

Sample is same as text Figure 2 and Table S-4. Table shows *RMR* separately for men and women for vaccinated (with indicated number of doses) versus unvaccinated persons, by calendar quarter over 1Q2021-4Q2022 for ages 15-59 and 60+, and 95% CIs. Values for 3Q-4Q 2021 are adjusted for IHIE undercount of vaccinated decedents during August-October 2021.

				**Ages 15-59**				**Ages 60+**	
		**Men**		**Women**		**Men**		**Women**	
**Period**	**Vaccine doses**	***RMR***	**95% CI (%)**	***RMR***	**95% CI (%)**	***RMR***	**95% CI (%)**	***RMR***	**95% CI (%)**
1Q 2021	1 or 2	0.8297	[0.1919, 3.5877]	0.2598	[0.0352, 1.9187]	0.1819***	[0.1290, 0.2565]	0.1984***	**[0.1443, 0.2729]**
2Q2021	1 or 2	0.1915**	[0.0692, 0.5298]	0.6789	[0.3270, 1.4095]	0.2993***	[0.2217, 0.4042]	0.2405***	**[0.1759, 0.3288]**
3Q2021	1 or 2	0.1687***	[0.1003, 0.2840]	0.1663***	[0.0909, 0.3045]	0.2569***	[0.2133, 0.3094]	0.2027***	[0.1634, 0.2515]
4Q2021	1 or 2	0.1381***	[0.0965, 0.1976]	0.1617***	[0.1076, 0.2429]	0.2999***	[0.2642, 0.3405]	0.2664***	[0.2328, 0.3049]
	3					0.0743***	[0.0417, 0.1324]	0.1325***	[0.0821, 0.2136]
1Q2022	1 or 2	0.3083***	[0.2215, 0.4292]	0.3874**	[0.2705, 0.5549]	0.5195***	[0.4562, 0.5916]	0.4371***	[0.3795, 0.5035]
	3	0.0773***	[0.0283, 0.2112]	0.1935***	[0.0879, 0.4259]	0.1562***	[0.1277, 0.1910]	0.1494***	[0.1207, 0.1850]
2Q2022	1 or 2	1.1246	[0.4489, 2.8170]	1.8687	[0.5641, 6.1907]	1.2988	[0.8641, 1.9523]	1.1224	[0.7147, 1.7627]
	3+	0.3202	[0.0417, 2.4576]	1.5396	[0.2941, 8.0592]	0.8162	[0.5400, 1.2336]	0.9056	[0.5857, 1.4002]
3Q2022	1 or 2	0.9693	[0.4452, 2.1103]	0.7776	[0.2915, 2.0746]	0.9790	[0.7042, 1.3612]	0.9955	[0.7088, 1.3982]
	3+	0.1687	[0.0224, 1.2704]	0.7909	[0.2538, 2.4651]	0.8221	[0.6114, 1.1056]	0.8511	[0.6257, 1.1578]
4Q2022	1 or 2	0.7822	[0.2766, 2.2121]	0.8308	[0.3619, 1.9074]	0.9119	[0.6506, 1.2782]	0.8581	[0.6253, 1.1777]
	3+	0.8173	[0.2305, 2.8973]	0.1484*	[0.0195, 1.1298]	1.0424	[0.7870, 1.3806]	0.7571*	[0.5704, 1.0049]

Figure S-1. Sample selection flowchart

Chart shows the process for selecting the sample of Indiana-resident decedents, aged 15+, used in the vaccine effectiveness analysis. After identifying natural deaths in each time periods, we record natural deaths by vaccination status (0, 1, 2, 3, or 4 doses). Table 2 in the text reports the number of COVID-19 deaths and other natural deaths by period, age bin, and vaccination status. Indiana population age 15+ is estimated at 5,459,479 as of July 1, 2020, based on Census data.

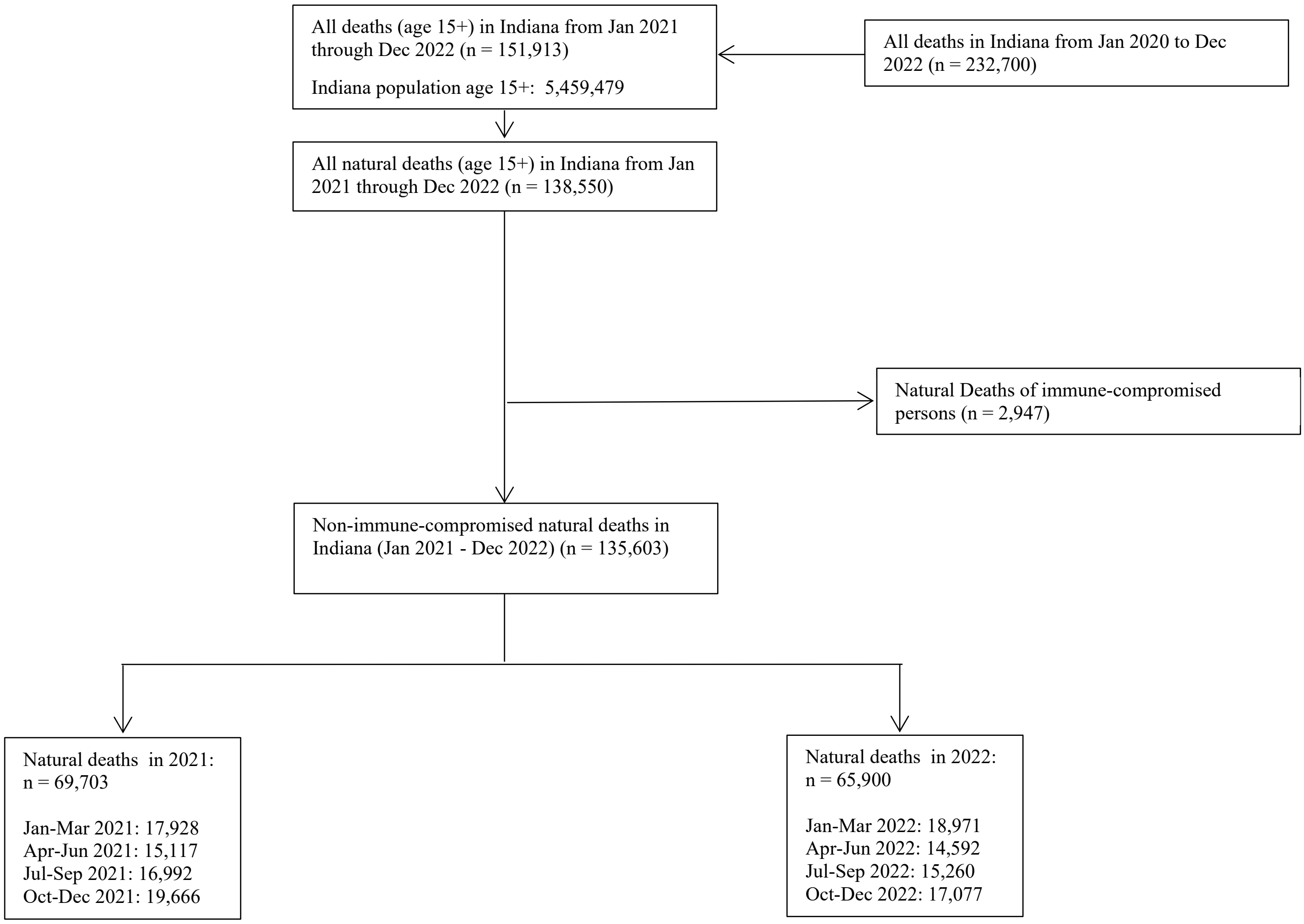

Figure S-2. Correlation between Natural Mortality Rates in 2019 and COVID-19 Mortality Rates in 2020.

Figure is similar to text Figure 1 but shows scatterplot of natural mortality *rates* in Indiana over April-December 2019 against COVID-19 mortality over April-December 2020, for groups defined by age (15-39, 40-49, 50-59, 60-69, 70-79, 80-89, 90+)*gender*race/ethnicity, best-fit regression line, and Pearson correlation coefficient. In contrast to text Figure 1, which provides a scatterplot for mortality *counts*, the population groups are not also divided into SES quintiles due to data limitations in Indiana population.

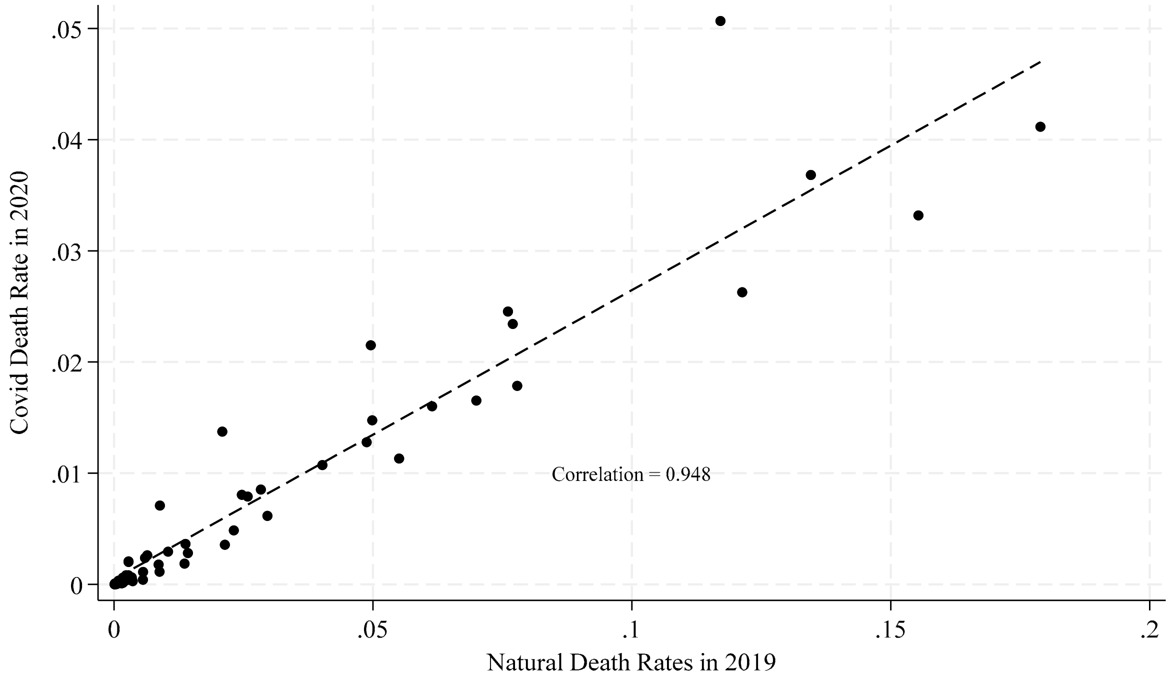

Figure S-3. Indiana Natural Deaths in 2019 vs. Non-COVID Natural Deaths in 2020

Figure shows scatterplot of natural mortality in Indiana over April-December 2019 against Non-Covid natural mortality over April-December 2020, for groups defined by age (18-39, 40-49, 50-59, 60-69, 70-79, 80-89, 90+)*gender*race/ethnicity*area-SES quintile, best-fit regression line, and Pearson correlation coefficient.

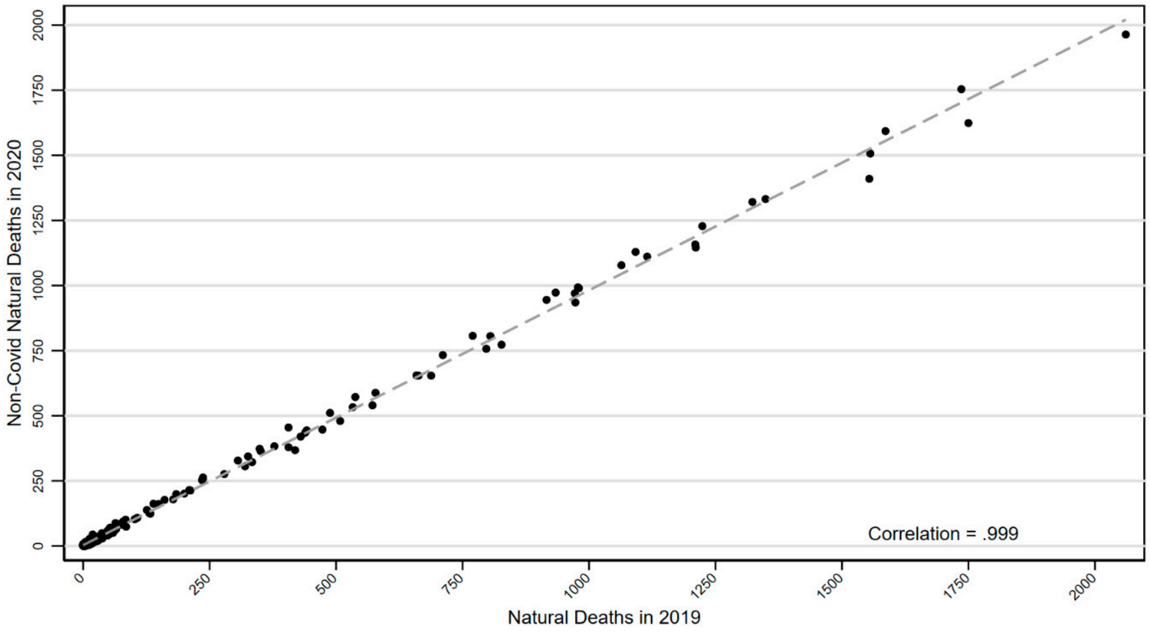

Figure S-4. Vaccination Rates for Adults by Age Group in Indiana

Vaccination rates over time for Indiana residents by age range, as a fraction of 2020 population. Age is measured at time of first dose

Panel A. Full Vaccination Rates

Full vaccination rates. defined as at least 1 J&J dose, or 2 mRNA doses (Pfizer, Moderna, or mixed), as percentage of population.

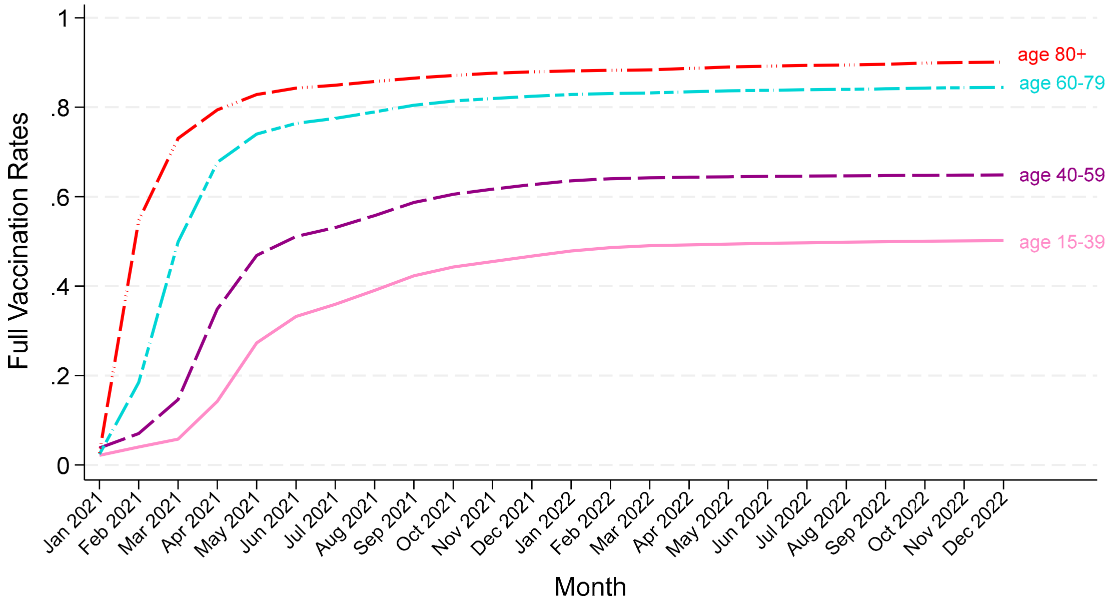

Panel B. At least Three Dose Vaccination Rates

Booster rates as percentage of people receiving full vaccination.

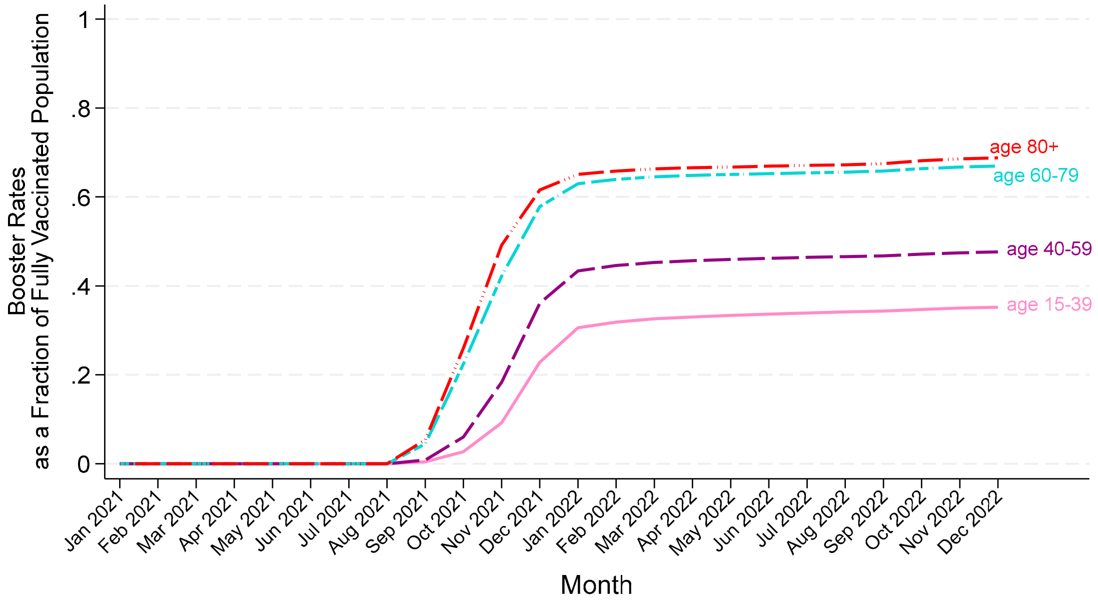

Panel C. At least Four Dose Vaccination Rates

Four-dose vaccination rates as percentage of people receiving full vaccination.

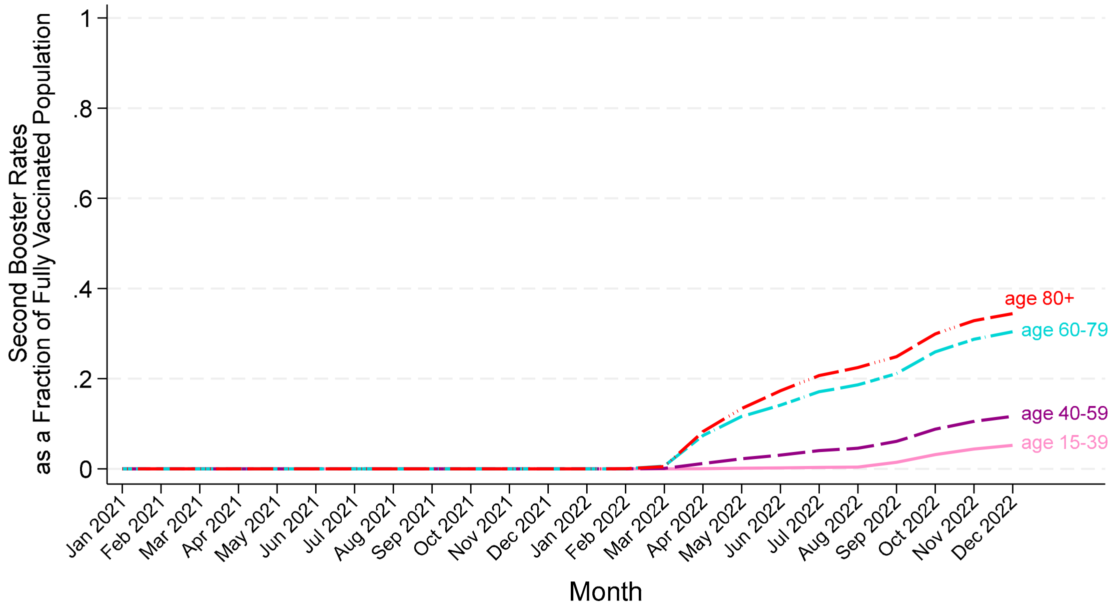

Figure S-5. Actual versus Predicted NC-NMR During Pandemic Period

Panel A. Figure shows monthly data for all natural deaths (including COVID-19 deaths) and natural non-COVID deaths, for Indiana for January 2017–December 2022. For the pandemic period starting March 2020, we show both actual and predicted natural non-COVID deaths. Predicted deaths during the pandemic period are based on mean deaths for the same calendar month during 2017–2019 to the same calendar month during the pandemic period. Natural deaths (including COVID-19 deaths) are shown as solid red line; this shows two large COVID-related peaks in late 2020 and late 2021–early 2022. Natural non-COVID deaths (all natural deaths minus COVID-19 deaths) are shown as solid blue line. Predicted natural non-COVID deaths are shown as dashed green line. **Panel B.** Figure is similar to Panel A, but: (i) is limited to pandemic period; (ii) drops line for all natural deaths; and (iii) adds dotted lines showing upper and lower 95% CIs around predicted non-COVID natural deaths.

Panel A. Actual versus Predicted Non-COVID Natural Mortality

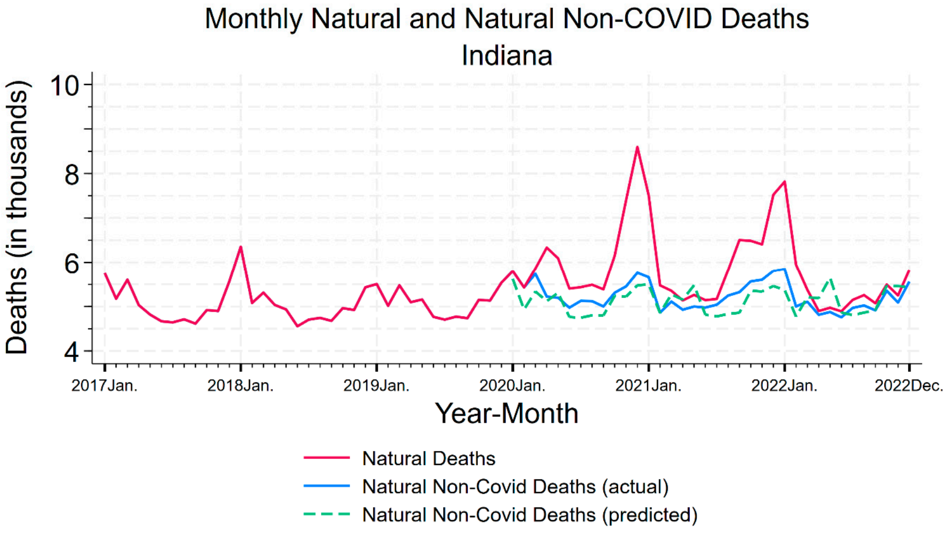

Panel B. Measured and Predicted Non-COVID Natural Deaths, with Confidence Intervals

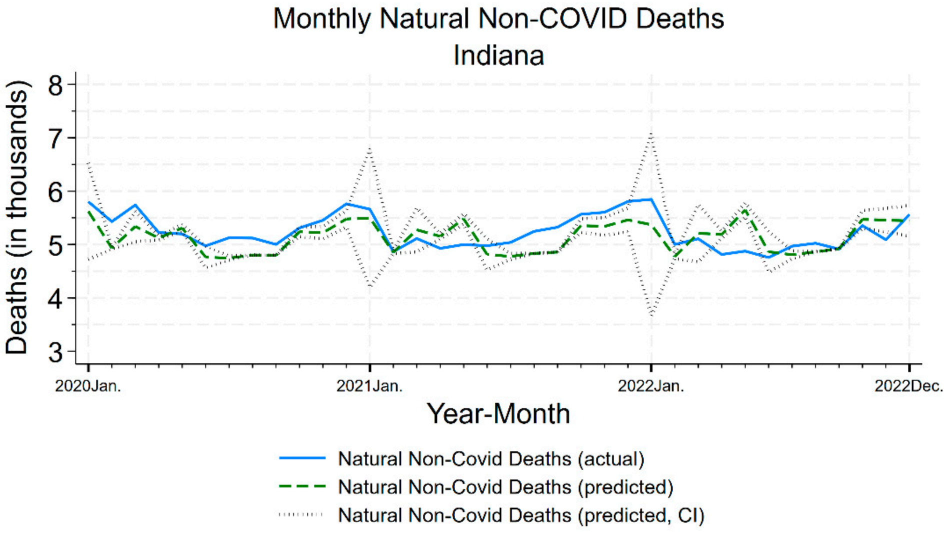

Figure S-6. Raw vs. Interpolated Deaths for Vaccinated Persons

Figure shows raw and interpolated counts of natural deaths of vaccinated decedents (1+ doses) in IHIE linked vaccination and mortality records, for ages 15-59 (**Panel A**), and ages 60+ (**Panel B**), from all natural causes (COVID and non-COVID), excluding immune-compromised persons. **Both panels**. Dashed line shows interpolated number of deaths for August-October 2021. Counts of vaccinated decedents are lagged by 30 days relative to day of first vaccine dose.

Panel A. Ages 15-59

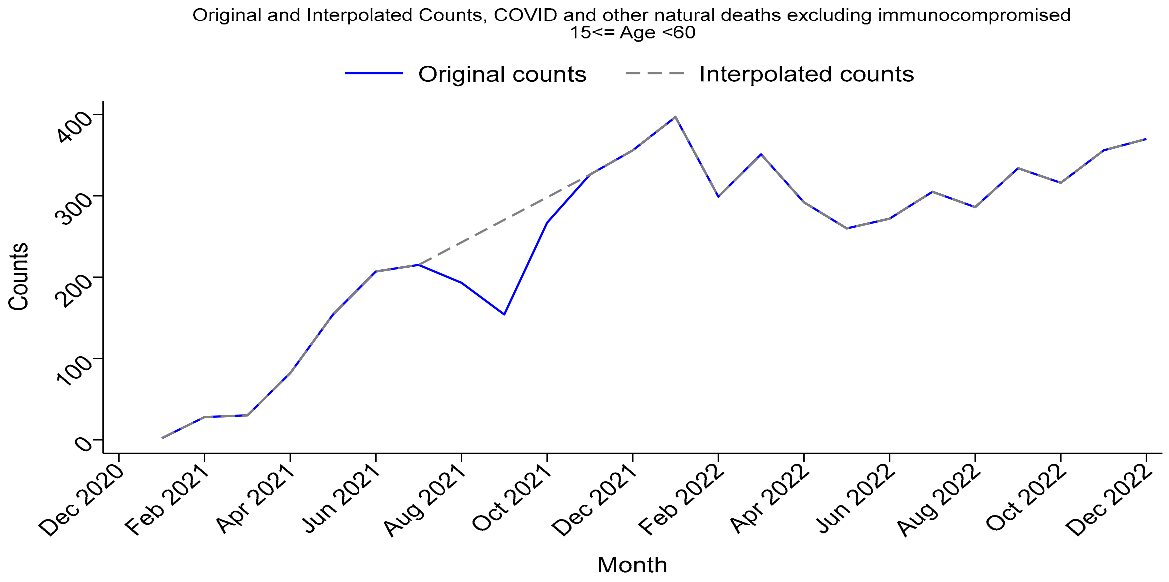

Panel B. Ages 60+

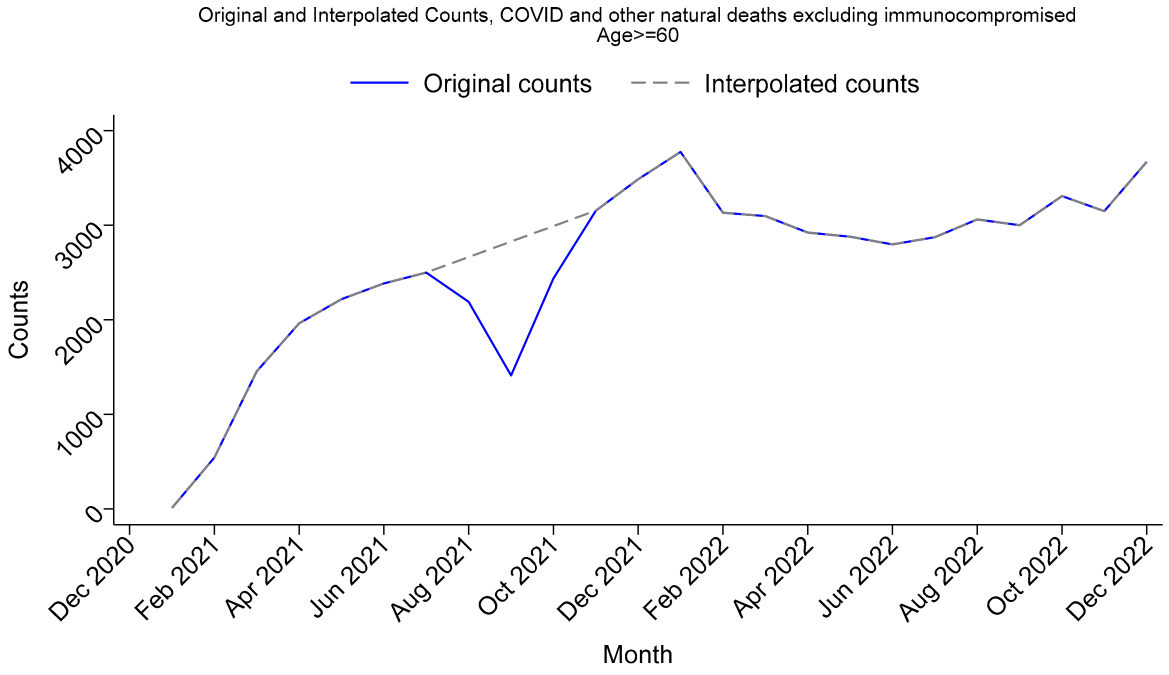

Figure S-7. Raw versus Adjusted *RMR*

Figure shows raw versus adjusted relative mortality risk (*RMR*), based on *CEMP*, by calendar quarter for ages 15-59 (**Panel A**) and 60+ (**Panel B**), for 1Q2021-4Q2022, for vaccinees aged 15+ with indicated number of vaccine doses. **Both panels**. Adjusted estimates for 3Q-4Q2021 are adjusted for IHIE undercount of vaccinated decedents during August-October 2021, based on interpolation shown in preceding figure.

Panel A. Ages 15-59, Note. *RMR*=0: Complete Protection; *RMR*=1: No Protection.

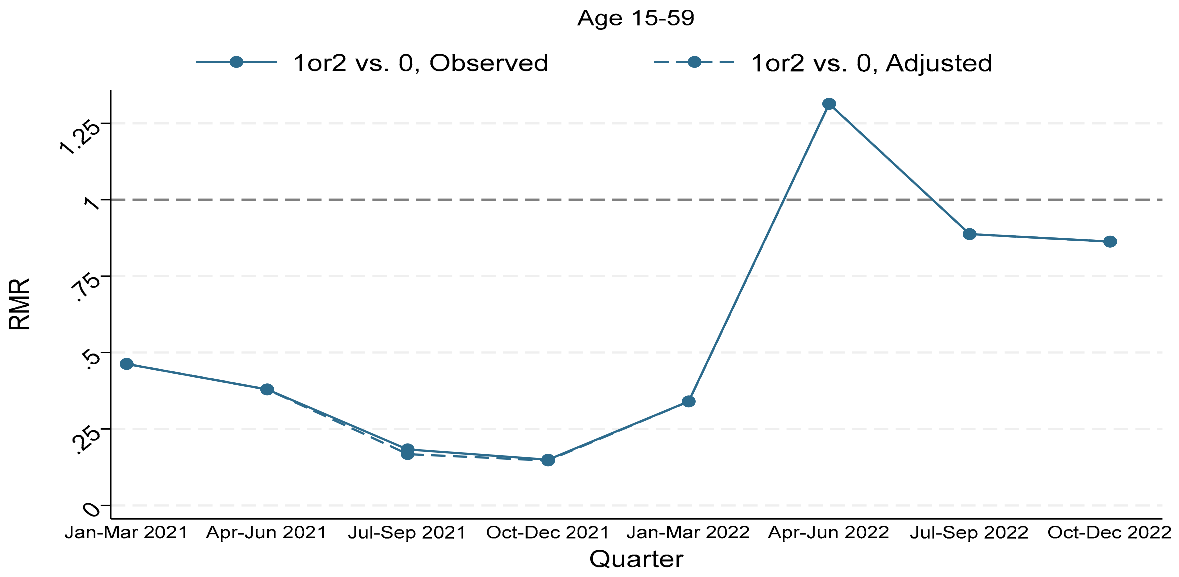

Panel B. Ages 60+, Note. *RMR*=0: Complete Protection; *RMR*=1: No Protection.

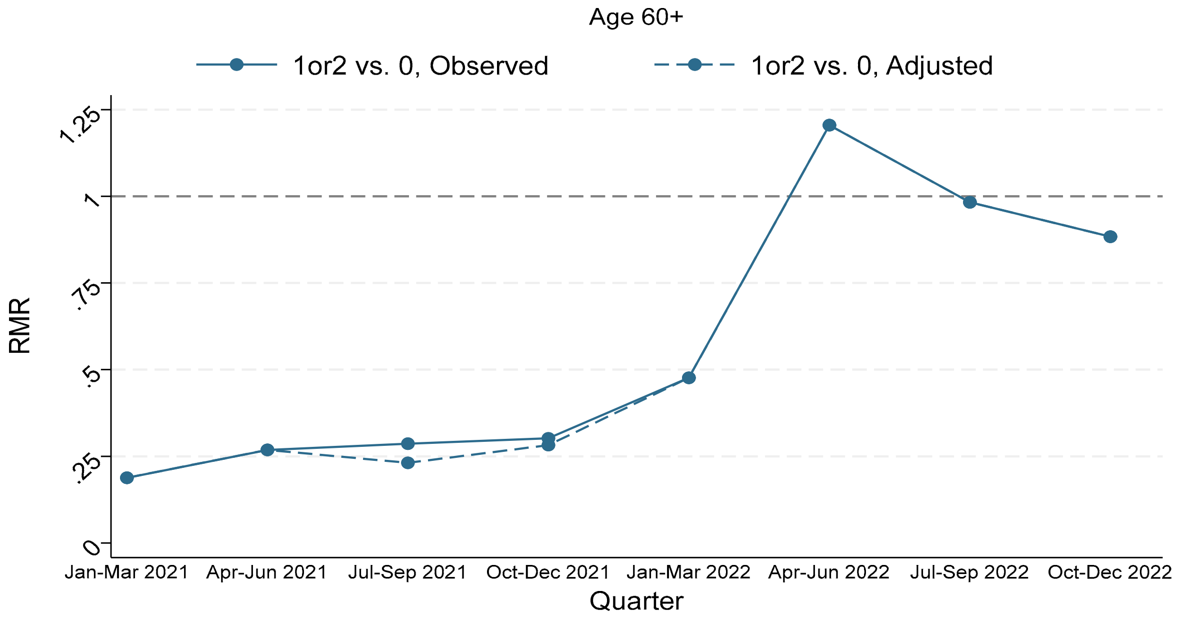

Figure S-8. Healthy Vaccinee Bias for Finer Age Groups

Figure is similar to text Figure 2, but shows healthy vaccinee bias correction factor (HVBFC) by calendar quarter for ages 15-39 (**Panel A**), 40-59 (**Panel B**), 60-79 (**Panel C**), and 80+ (**Panel D**), for 1Q2021-4Q2022, for vaccinees aged 15+ with indicated number of vaccine doses. **All panels**. HVBCF is ratio of non-COVID NMR with indicated numbers of vaccine doses for vaccinated/non-COVID NMR for unvaccinated. Estimates for 3Q-4Q2021 are adjusted for IHIE undercount of vaccinated decedents during August-October 2021. See Table S-4 for underlying data. Sample excludes immune compromised persons. Short vertical lines show 95% confidence intervals. Confidence intervals are especially tight for ages 80+, and sometimes not visible in Panel D. Higher HVBCF indicates less bias.

Panel A. Ages 15-39

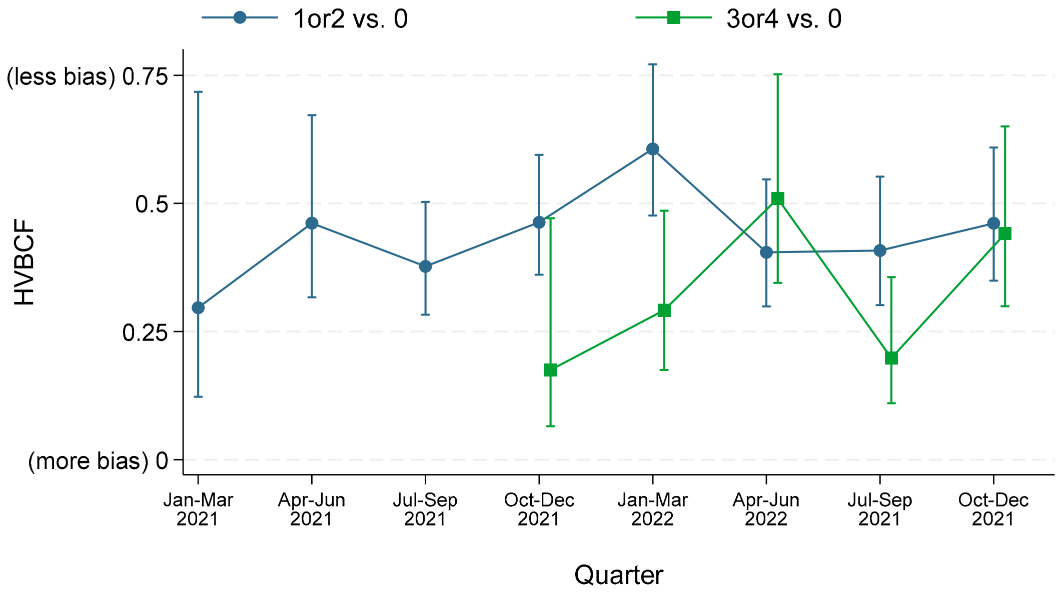

Panel B. Ages 40-59

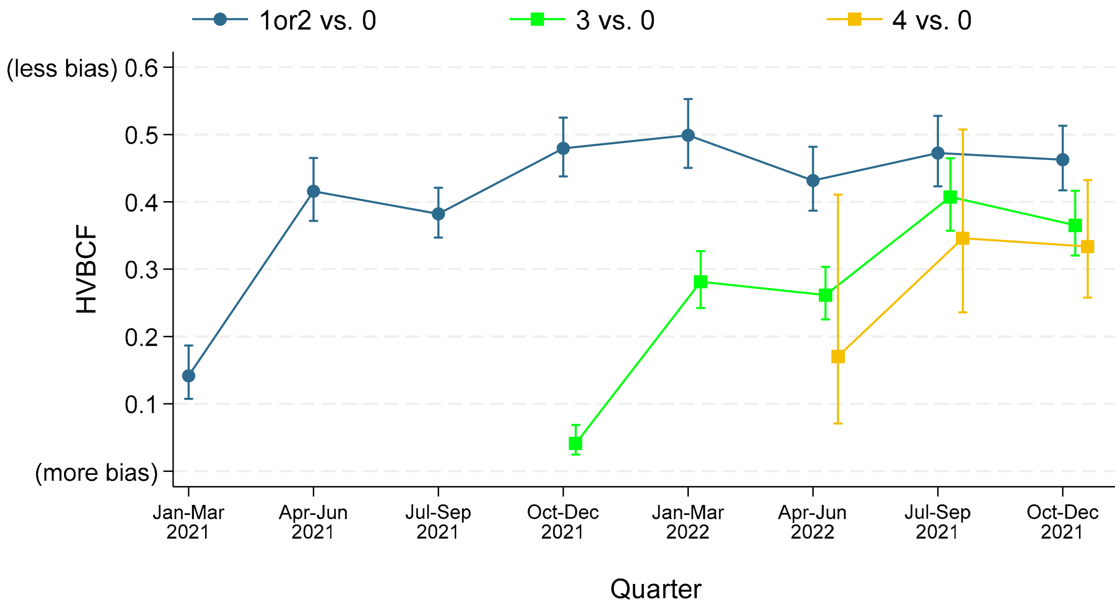

Panel C. Ages 60-79

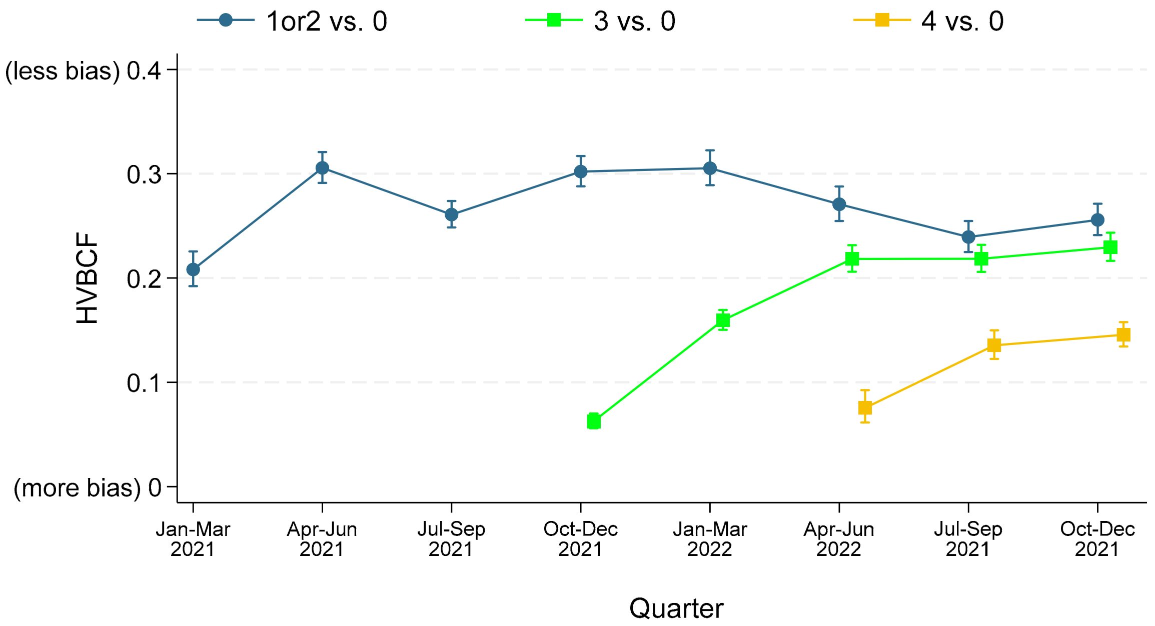

Panel D. Ages 80+

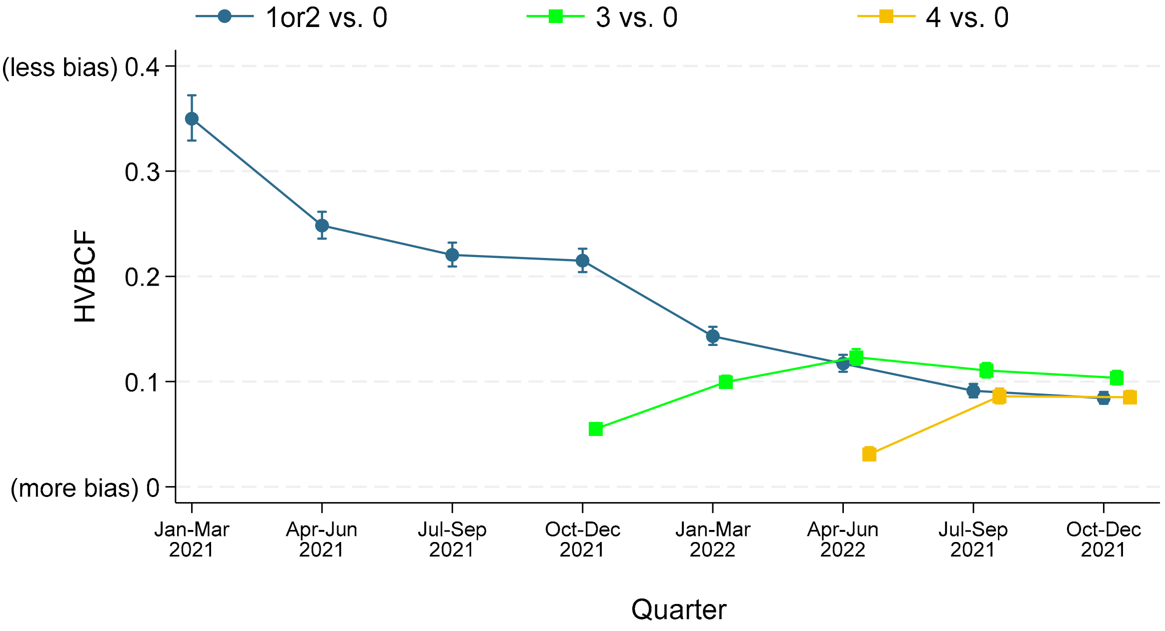

Figure S-9. Relative Mortality Risk for Finer Age Groups

Figure is similar to text Figure 3, but shows relative mortality risk (*RMR*) by calendar quarter for ages 15-39 (**Panel A**), 40-59 (**Panel B**), 60-79 (**Panel C**), and 80+ (**Panel D**), for 1Q2021-4Q2022, for vaccinees aged 15+ with indicated number of vaccine doses. Panels C and D show *RMR* separately for 3-dose and 4-dose recipients. **All panels**. Estimates for 3Q-4Q2021 are adjusted for IHIE undercount of vaccinated decedents during August-October 2021. Vertical lines show 95% confidence intervals (CIs). Upper end of CI is generally truncated at 2.5. CI not shown if number of COVID deaths is zero.

Panel A. Ages 15-39, Note. *RMR*=0: Complete Protection; *RMR*=1: No Protection.

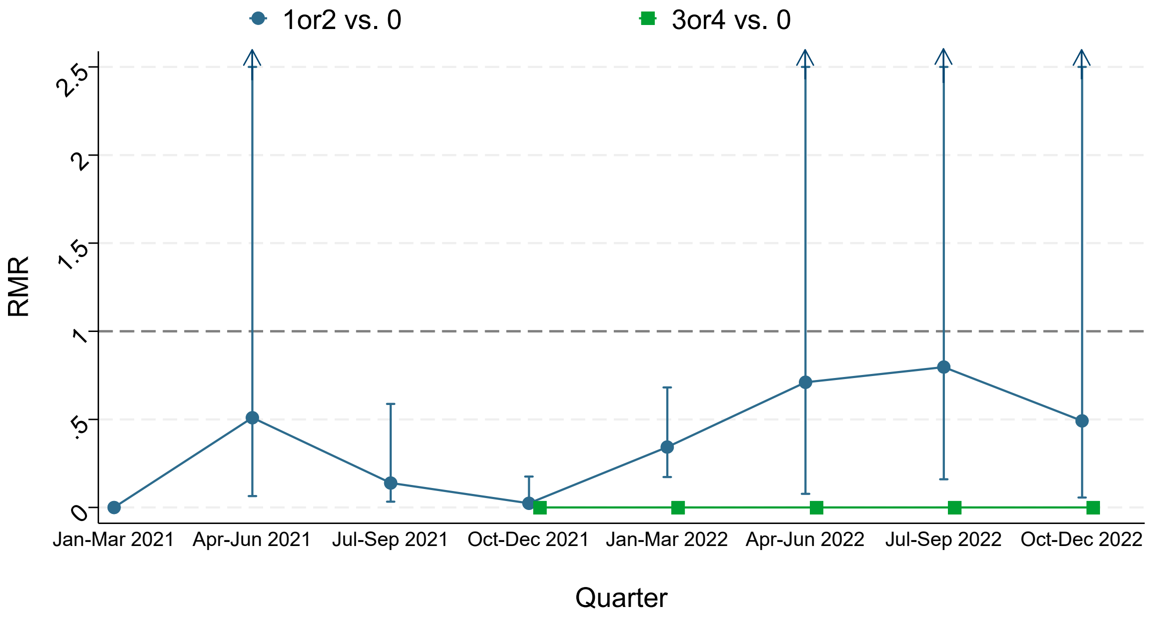

Panel B. Ages 40-59, Note. *RMR*=0: Complete Protection; *RMR*=1: No Protection.

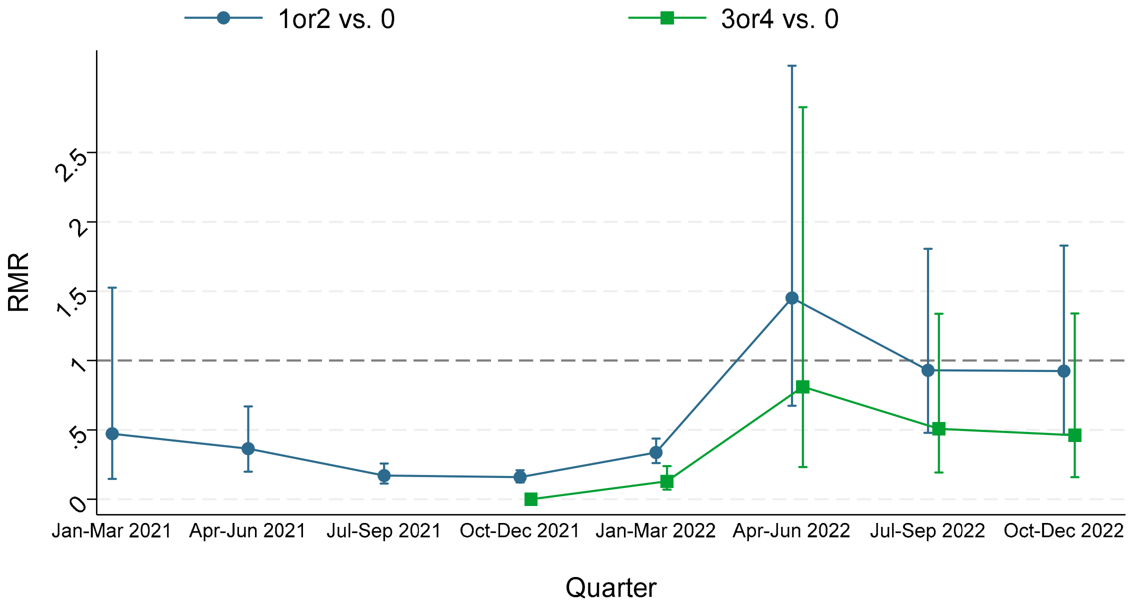

Panel C. Ages 60-79, Note. *RMR*=0: Complete Protection; *RMR*=1: No Protection.

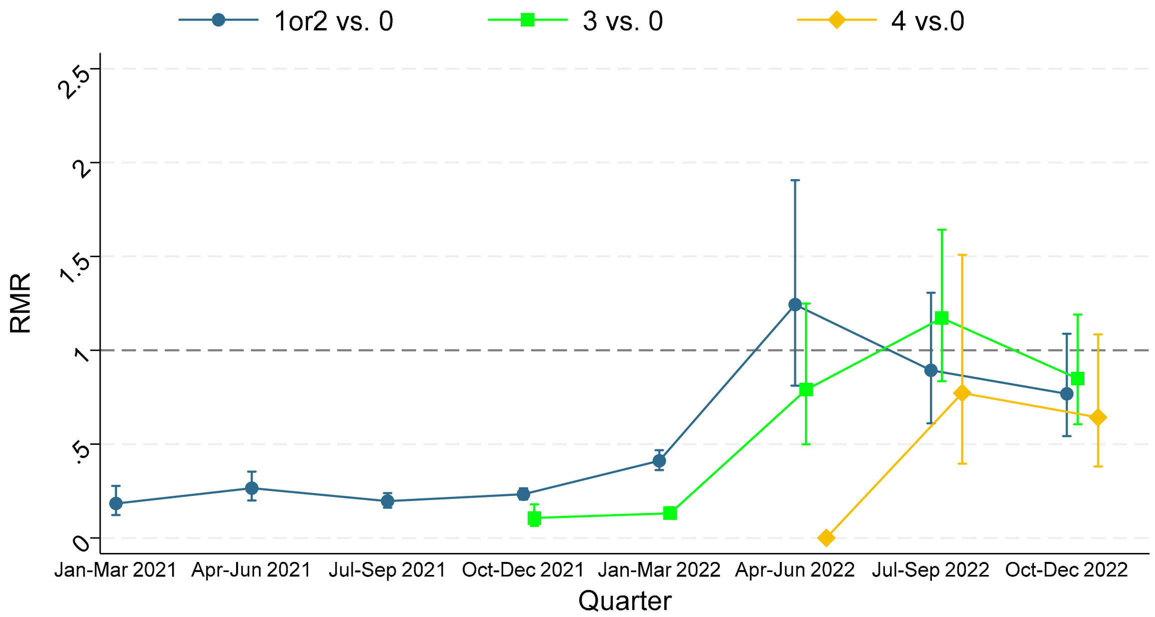

Panel D. Ages 80+, Note. *RMR*=0: Complete Protection; *RMR*=1: No Protection.

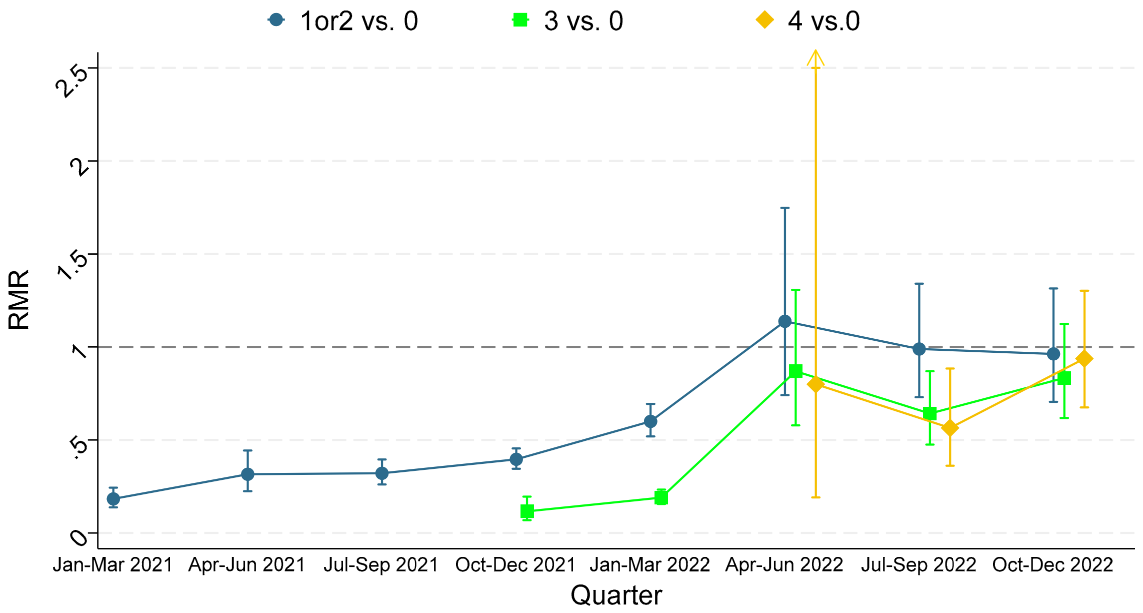

Figure S-10. Plot of Vaccine Effectiveness Instead of *RMR*

Figure shows vaccine effectiveness based on *CEMP* (VE^{CEMP}), by calendar quarter for vaccinees aged 15+ with indicated number of vaccine doses, for 1Q2021-4Q2022. This figure corresponds to text Figure 3, which plots $RMR^{CEMP} = 1 - VE^{CEMP}$. **Left-hand side graph**. Ages 15-59. 3 and 4 doses are combined because very few people in this age range received a fourth dose prior to 4Q 2022. **Right-hand side graph**. Age 60+. **Both graphs**. Estimates for 3Q-4Q2021 are adjusted for IHIE undercount of vaccinated decedents during August-October 2021. Vertical lines show 95% confidence intervals.

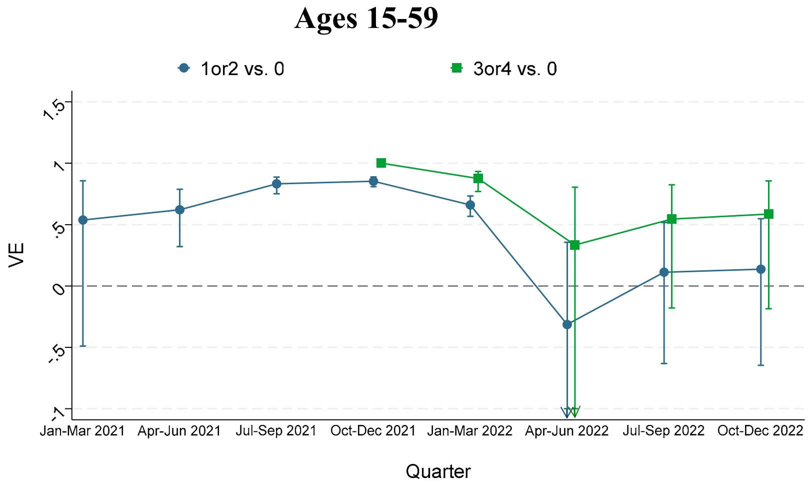

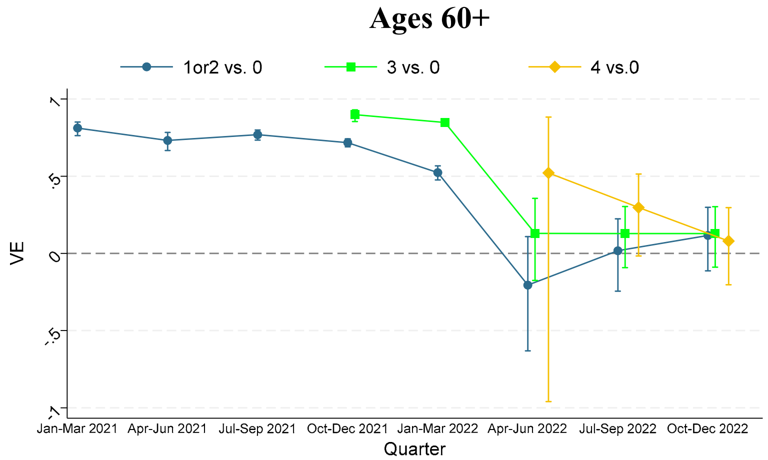

Figure S-11. Relative Mortality Risk for Men vs. Women

Figure shows relative mortality risk (*RMR*) separately for men and women, by calendar quarter for ages 15-59 (**Panel A**) and 60+ (**Panel B**) for 1Q2021-4Q2022, for vaccinees with indicated number of vaccine doses. **Both panels**. Estimates for 3Q-4Q2021 are adjusted for IHIE undercount of vaccinated decedents during August-October 2021. Vertical lines show 95% confidence intervals (CIs). Upper end of CI is generally truncated at 2.5. CI not shown if number of COVID deaths is zero.

Panel A. Ages 15-59

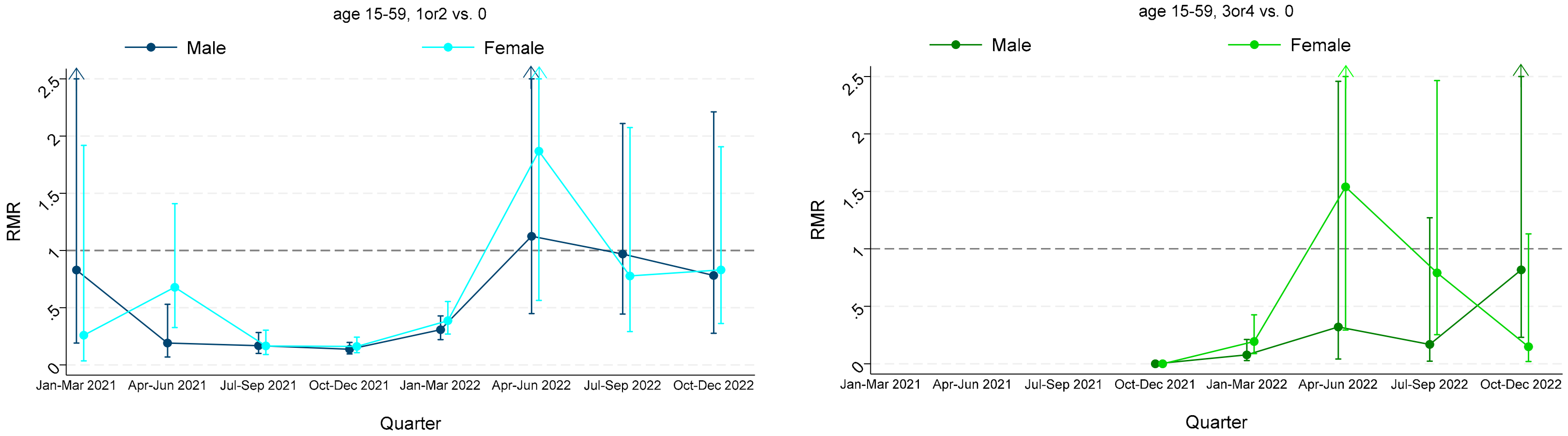

Panel B. Ages 60+

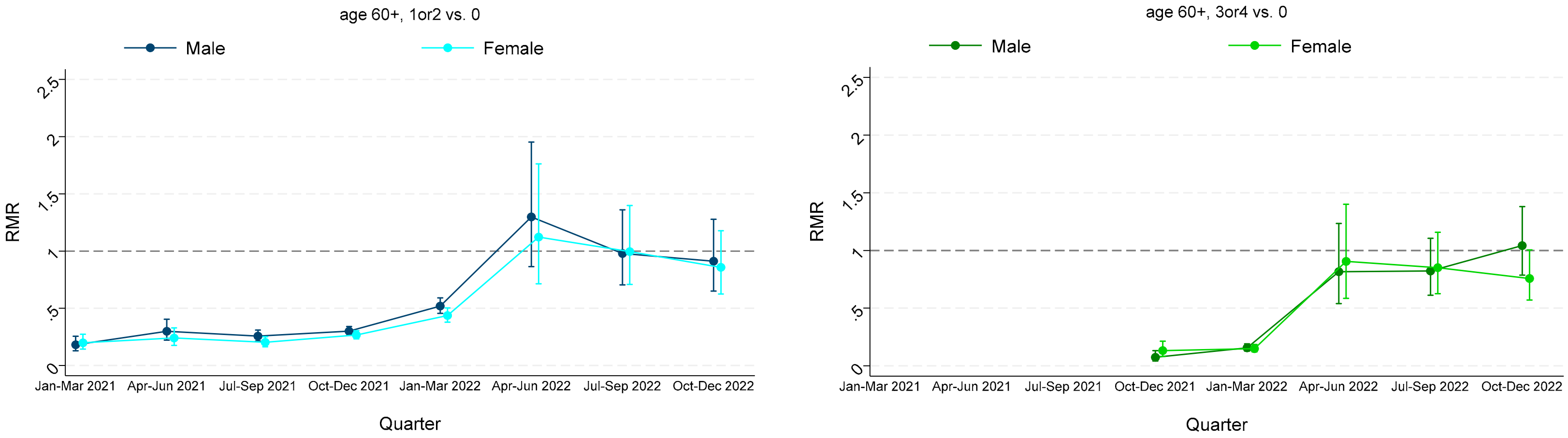

Figure S-12. RMR^{raw} versus RMR^{CEMP}

Figure compares RMR^{CEMP} to RMR^{raw}, for indicated age groups, time periods, and number of vaccine doses. Sample is same as text Figure 2. **Panel A**. 1 or 2 doses. **Panel B**. 3 or 4 doses. **Both panels.** Left-hand graphs are for ages 15-59. Right-hand graphs are for ages 60+. Sample excludes immune-compromised persons. *RMR* for 3Q-4Q 2021 is adjusted for IHIE undercount of vaccinated decedents during August-October 2021.

Panel A. 1 or 2 doses

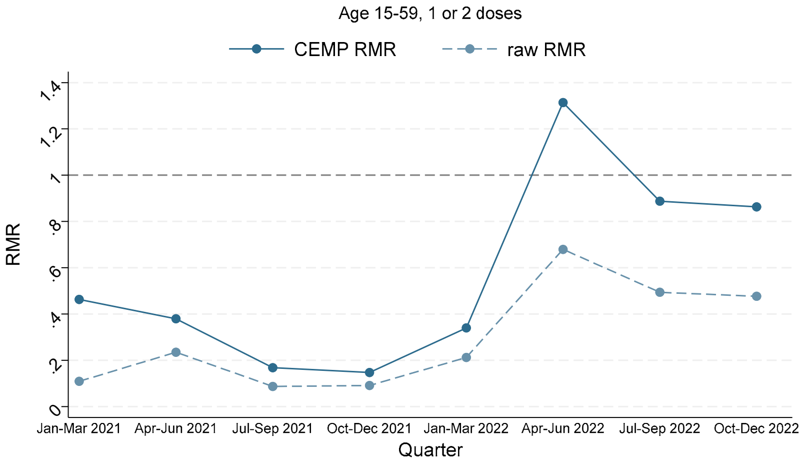

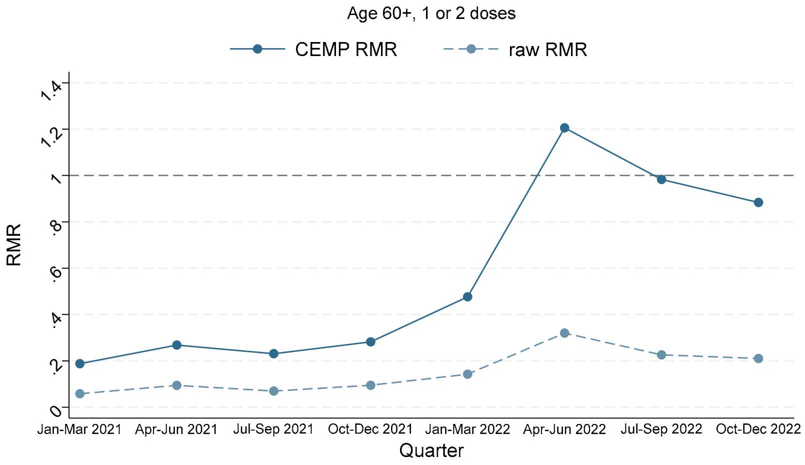

Panel B. 3 or 4 doses

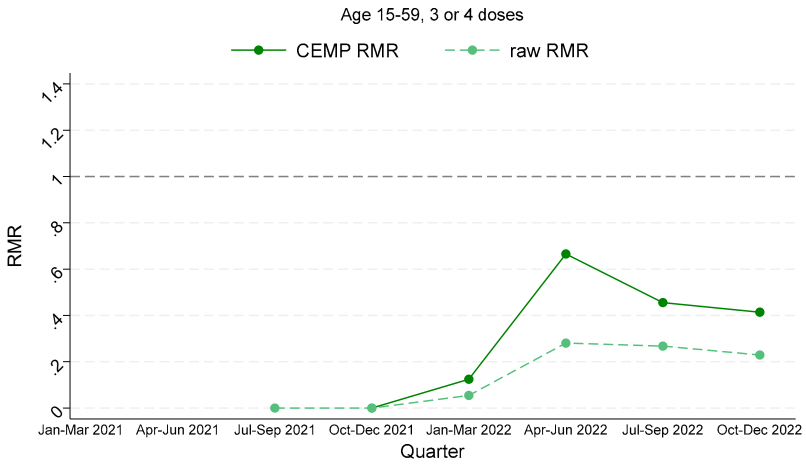

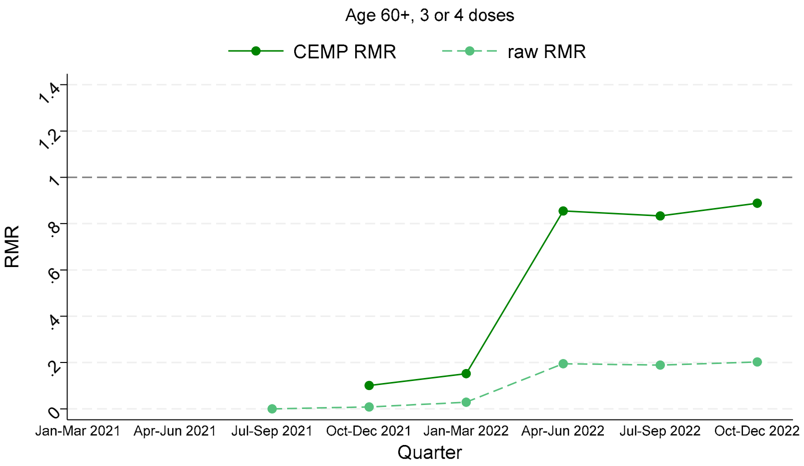

Figure S-13. All-Cause Natural Mortality Relative to Vaccination Date

Figure shows daily number of all-cause natural mortality deaths among persons vaccinate with indicated number of doses, based on days since vaccination. **Panel A**. Days since first dose for all 1-dose recipients. **Panel B**. Days since first dose for all 2-dose recipients. **Panel C**. Days since first dose for all 3-dose recipients. **Panel D**. Days since first dose for all 4-dose recipients. **All panels**. Days are actual days from vaccination, without the 30-day time shift used in the text. Graphs are truncated at 200 days since vaccination for doses 1, 2, and 3; and at 150 days for dose 4. Dashed vertical line shows 30 days after vaccination date.

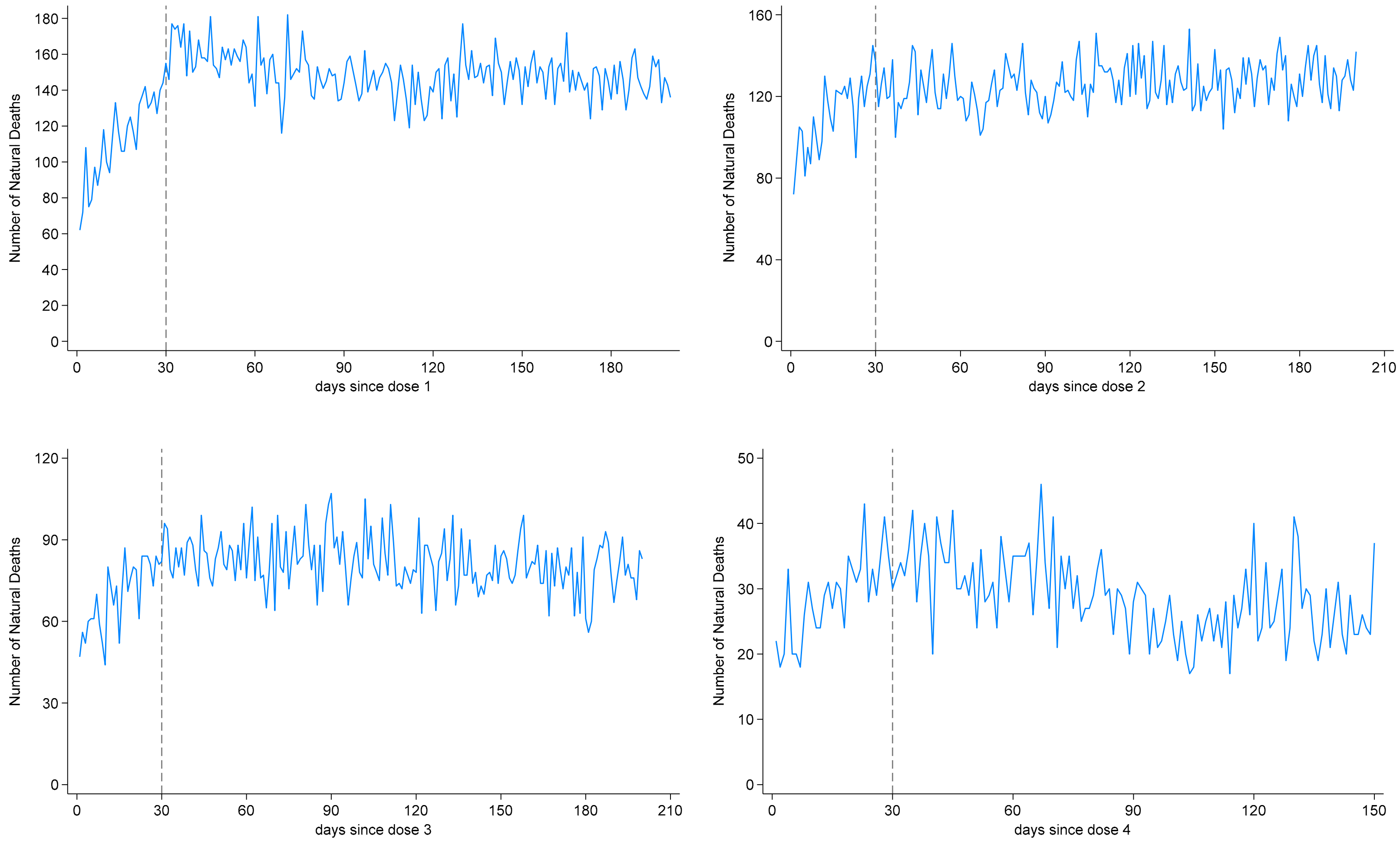

Figure S-14. *RMR* in Indiana vs. Milwaukee County

Figure shows relative mortality risk (*RMR*), based on *CEMP*, by calendar quarter for ages 15-59 (**Panel A**) and 60+ (**Panel B**), for 1Q2021-4Q2022, for vaccinees aged 15+ with indicated number of vaccine doses in Indiana compared to *RMR* for Milwaukee County, Wisconsin (MKE). **Both panels**. Milwaukee data starts at age 18 (vs. 15 in Indiana). Indiana estimates for 3Q-4Q2021 are adjusted for IHIE undercount of vaccinated decedents during August-October 2021. Vertical lines show 95% CIs. CI not shown if number of COVID deaths is zero.

Panel A. *RMR* vaccinated vs unvaccinated: Ages 15-59 (IN) or 18-59 (Milwaukee)

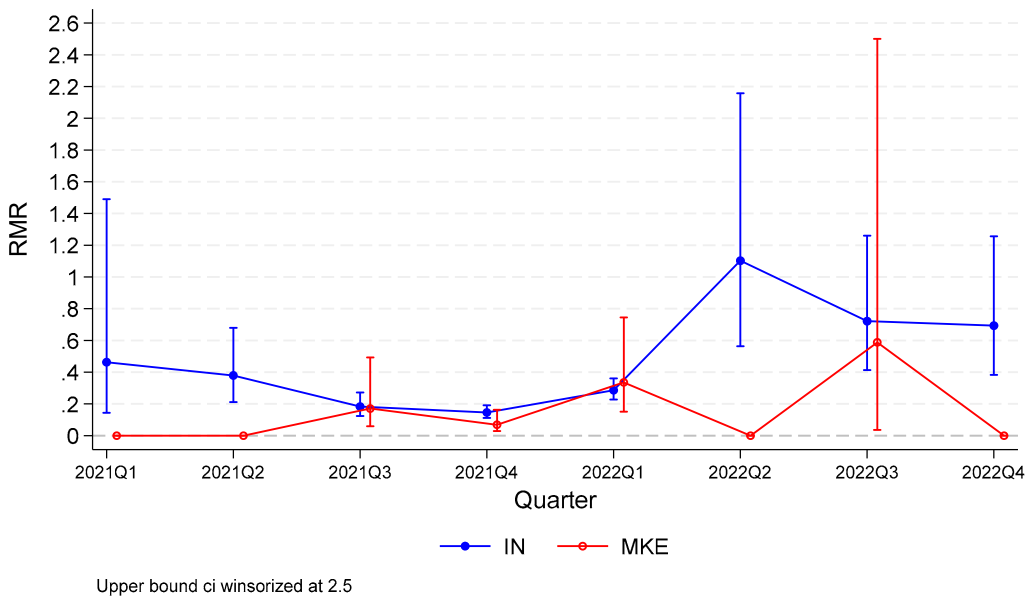

Panel B. *RMR* 1 or 2 doses vs. 0: Ages 60+

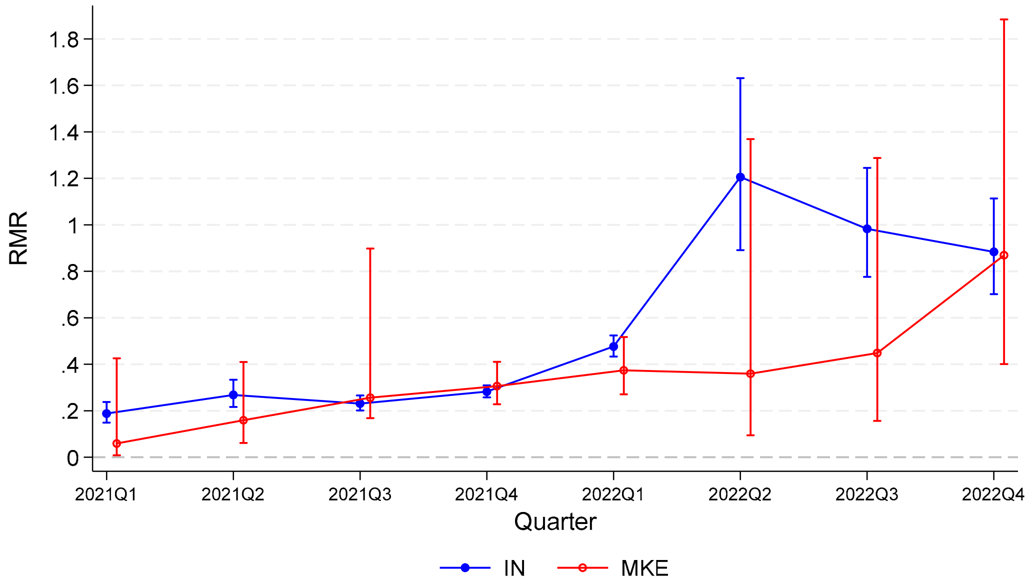

Supplement: Supplementary file 1 [file vaccines-13-01235-s001.zip › vaccines-3999785-supplementary.pdf]
